# Updated knowledge and a proposed nomenclature for nuclear receptors with two DNA binding domains (2DBD-NRs)

**DOI:** 10.1371/journal.pone.0286107

**Published:** 2023-09-12

**Authors:** Wenjie Wu, Philip T. LoVerde

**Affiliations:** Departments of Biochemistry and Structural Biology University of Texas Health, San Antonio, Texas, United States of America; Newcastle University, UNITED KINGDOM

## Abstract

Nuclear receptors (NRs) are important transcriptional modulators in metazoans. Typical NRs possess a conserved DNA binding domain (DBD) and a ligand binding domain (LBD). Since we discovered a type of novel NRs each of them has two DBDs and single LBD (2DBD-NRs) more than decade ago, there has been very few studies about 2DBD-NRs. Recently, 2DBD-NRs have been only reported in Platyhelminths and Mollusca and are thought to be specific NRs to lophotrochozoan. In this study, we searched different databases and identified 2DBD-NRs in different animals from both protostomes and deuterostomes. Phylogenetic analysis shows that at least two ancient 2DBD-NR genes were present in the urbilaterian, a common ancestor of protostomes and deuterostomes. 2DBD-NRs underwent gene duplication and loss after the split of different animal phyla, most of them in a certain animal phylum are paralogues, rather than orthologues, like in other animal phyla. Amino acid sequence analysis shows that the conserved motifs in typical NRs are also present in 2DBD-NRs and they are gene specific. From our phylogenetic analysis of 2DBD-NRs and following the rule of Nomenclature System for the Nuclear Receptors, a nomenclature for 2DBD-NRs is proposed.

## Introduction

**Fig 1:**
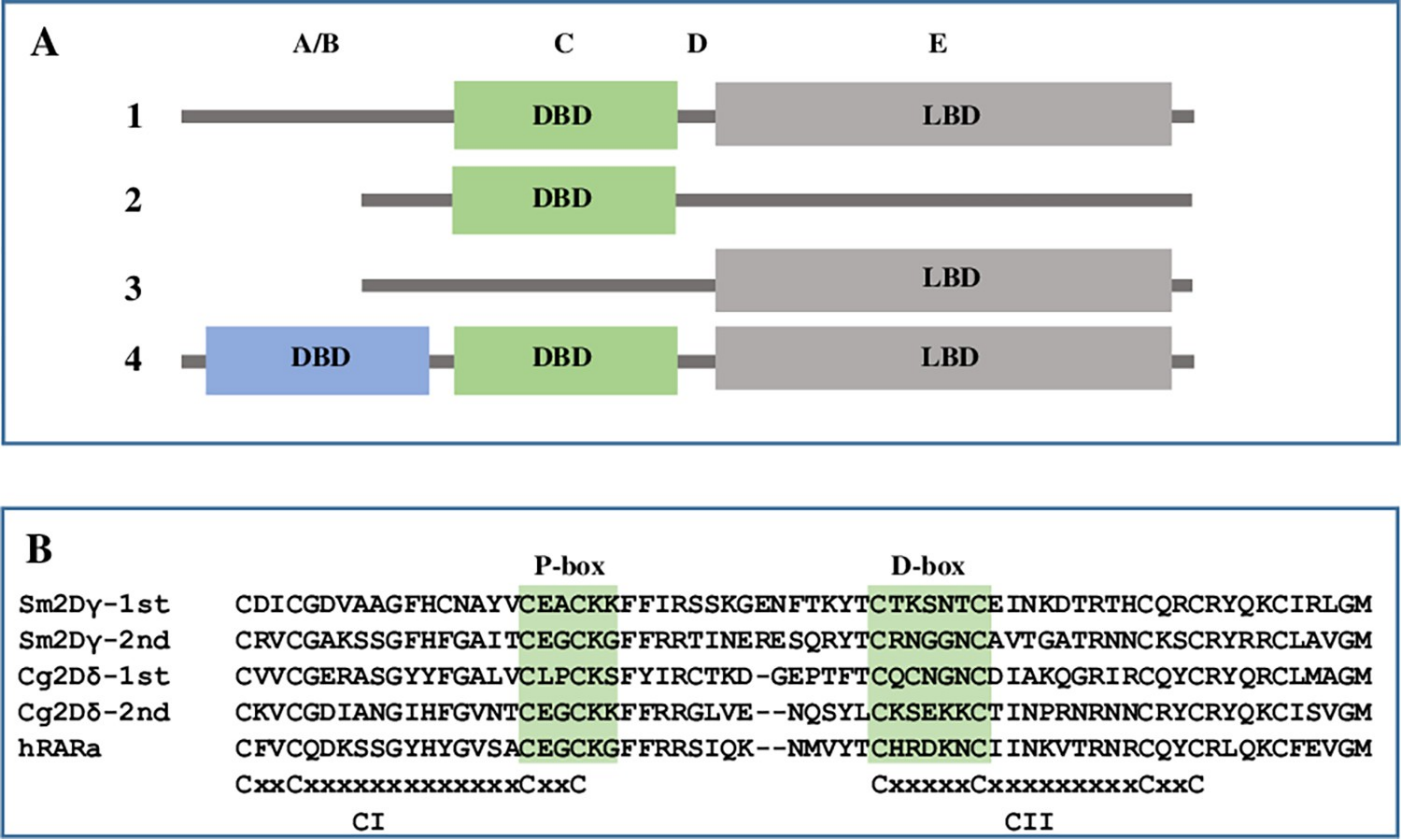
Modular structure of nuclear receptor. **A**. Modular structure of nuclear receptors. 1. Typical NR with single DBD and a LBD, 2. Atypical NR with only a DBD but without LBD, 3. Atypical NR with only a LBD but without DBD, 4. Atypical NR with two DBDs and a LBD. **B**. DBD sequence alignment shows the two zinc fingers and the conserved P-box and D-Box. The first zinc finger (CI) with a conserved motif sequence of C-X2-CX13-C-X2-C; the second zinc finger (CII) with a conserved motif sequence of C-X5-C-X9-C-X2-C. C: cysteine residue, X followed by a number that indicates the number of amino acids between the Cs (Cys). Sm2Dγ-1st: the first DBD of *Schistosoma mansoni* 2DBD-NRγ (Sm2DBD-NRγ, GenBank: AAW88550), Sm2Dγ-2nd: the second DBD of Sm2DBD-NRγ, Cg2Dδ-1st: the first DBD of *Crassostrea gigas* 2DBD-NRδ (Cg2DBD-NRδ, GenBank: XP_011428801), Cg2Dδ-2nd: the second DBD of Cg2DBD-NRδ, hRARα: human RARα (GenBank: AAD05222.1).

Nuclear receptors (NRs) are important transcriptional modulators in metazoans, members of the NR superfamily are characterized by a modular structure: typical NRs contain an N terminal A/B domain, a C domain (DNA binding domain, DBD), a D domain (hinge) and an E domain (ligand binding domain, LBD) (Fig 1A). NRs regulate transcription through binding to the promoter region of their target gene by the DBD and activation or repression of mRNA synthesis through co-regulators bound to the LBD [1–5]. Atypical NRs exist in some animals, NRs with DBD but no LBD are found in arthropods and nematodes [6–9], NRs missing DBD but contain a LBD are present in vertebrates [10, 11] (Fig 1A).

In 2006, we reported our result of identification and isolation of partial cDNAs of three NRs from blood fluke *Schistosoma mansoni*, each of them possesses two tandem DBDs (2DBD-NRs) [12], they were then verified by the *S*. *mansoni* Genome Project [13] and the full length cDNAs were isolated [14]. This was the first time to demonstrate that NR possesses a novel modular structure: A/B-DBD-DBD-hinge-LBD organization [14] (Fig 1A). By an extensive search of whole genomic sequence (WGS) databases, we further demonstrated 2DBD-NRs were present in other animals including Platyhelminths *Schmidtea mediterranea*, *Dugesia japonica* and Mollusca *Lottia gigantean*. Phylogenetic analysis of DBD sequences showed that all of these 2DBD-NRs belonged to a monophyletic group and suggested that 2DBD-NRs originated from a common ancestor gene [14]. Recently, 2DBD-NRs were only identified and/or isolated in Platyhelminths [12, 14–21] and in Mollusca [22, 23]. Until now, there are fewer studies on 2DBD-NRs. Our study showed that Sm2DBD-NRα could form a homodimer but could not form a heterodimer with RXRs [14]. By searching structurally homologous sequences in the protein data bank (PDB), Alvite et al. showed that unsaturated fatty acids were preferred ligands by a *Echinococcus granulosus* 2DBD-NR (Eg2DBDa.1) [15]. Tharp et al. showed that *S*. *mediterranea* 2DBD-NR (nhr-1) was only detected in male and female accessory reproductive organs, and they suggested that *S*. *mediterranea* 2DBD-NR (nhr-1) was required for planarian reproductive maturation [18].

In this study, 2DBD-NRs are mined from different databases and are phylogenetically analyzed. From our phylogenetic analysis of 2DBD-NRs and following the rule of Nomenclature System for the Nuclear Receptors, a nomenclature for 2DBD-NRs is proposed.

## Materials and methods

### 1. Data mining

2DBD-NRs were mined from The National Center for Biotechnology Information (NCBI) protein database (https://www.ncbi.nlm.nih.gov/), the Ensembl Genomes project *Capitella_teleta* database (https://metazoa.ensembl.org/Capitella_teleta/Tools/Blast) [24], *Notospermus geniculatus* database (https://marinegenomics.oist.jp/nge_v2/blast/search?project_id=52) and *Phoronis australis database* (https://marinegenomics.oist.jp/pau_v2/viewer/info?project_id=51) [25]. Amino acid sequences of both DBDs of Sm2DBD-NRα (AH013462) and Cg2DBD-NR (XP_019919868) were used as the query to pblast (with E-value threshold: 1e-1) against all available NCBI protein databases, and tblastn (with E-value threshold: 1e-1) against *C*. *teleta*, *N*. *geniculatus* and *P*. *australis* genome databases. Any sequence that contains a zinc finger structure of the DBD of NRs (Cys-X2-Cys-X13-Cys-X2-Cys or Cys-X5-Cys-X9-Cys-X2-Cys) was retained (Fig 1B). After careful check by eye, all amino acid sequences containing two DBDs, partial two DBDs or highly conserved sequence to 2DBD-NRs were retained.

### 2. Phylogenetic analysis

Phylogenetic trees of 2DBD-NRs were constructed from deduced amino acid sequences of both the first and the second DBDs. The amino acid sequences were aligned with ClustalW [26], phylogenetic analysis of the data set was carried out using Bayesian inference MrBAYES v3.1.1 [27] as in our previous study [21]. Only Bayesian inference was carried out in this study because our previous study demonstrated that Bayesian inference highly supported phylogenetic analysis of NRs more than other methods [21]. The trees were started randomly with a mix amino acid replacement model + gamma rates. Two sets of four simultaneous Markov chains were run for 5 million generations and the trees were sampled every 100 generations. The Bayesian posterior probabilities (BPPs) were calculated using a Markov chain Monte Carlo (MCMC) sampling approach implemented in MrBAYES v3.1.1 and the burn-in value was set at 12,500.

### 3. Amino acid sequence analysis

Amino acid sequences of every 2DBD-NRs including full length or partial sequences were aligned using ClustalW [26] and the conserved sequences were identified by the most common amino acid residue at each position. Sequence Logos were created online (https://weblogo.berkeley.edu/logo.cgi) [28].

## Results and disscussion

### 1. Identification and phylogenetic analysis of 2DBD-NRs

2DBD-NRs are identified in different animal species including those from protostome Spiralia (Rotifera, Brachiopoda, Mollusca, Annelida, Platyhelminths, Brachiopoda, Nemertea and Phoronida) and Ecdysozoa (Nematoda), and from deuterostome Unchordata (Echinodermata and Chordata (Cephalochordata). Previously, 2DBD-NRs are thought to be lophotrochozoan-specific [18], this study shows that 2DBD-NRs broadly exist in protostome and deuterostome species, this further indicates that 2DBD-NR gene was already present in the urbilaterian, a common ancient ancestor of protostomes and deuterostomes.

**Fig 2:**
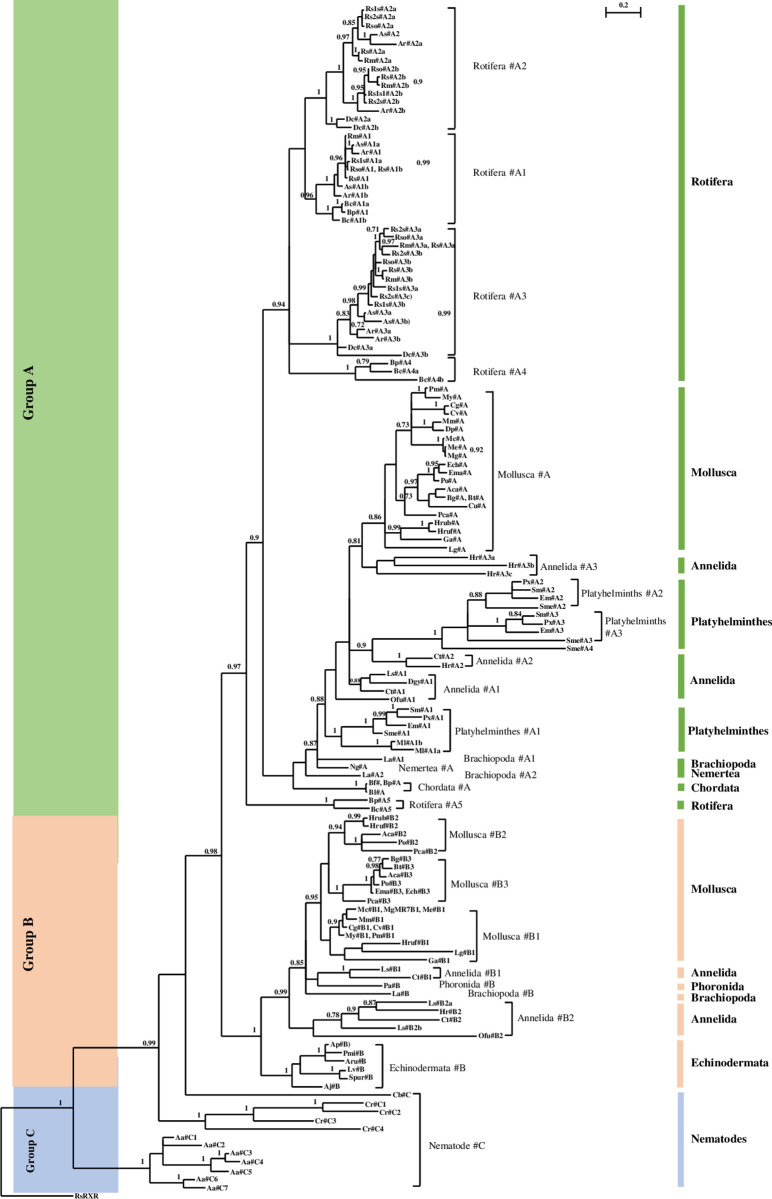
Bayesian phylogenetic analysis of 2DBD-NRs. Bayesian phylogenetic tree is constructed with the amino acid sequence of both the first and second DBD. The BPP values are shown above each branch or after the name of the NR, branches under PPs of 0.5 are shown as polytomies. **Aa**: *Aphelenchus avenae*, **Aca**: *Aplysia californica*, **Aj**: *Anneissia japonica*, **Ap**: *Acanthaster planci*, **Ar**: *Adineta ricciae*, **Aru**: *Asterias rubens*, **As**: *Adineta steineri*, **Bc**: *Brachionus calyciflorus*, **Bf**: *Branchiostoma floridae*, **Bg**: *Biomphalaria glabrata*, **Bl**: *Branchiostoma lanceolatum*, **Bt**: *Bulinus truncatus*, **Bp**: *Brachionus plicatilis*, **Cb**: *Caenorhabditis brenneri*, **Cg**: *Crassostrea gigas*, **Cr**: *Caenorhabditis remanei*, **Cu**: *Candidula unifasciata*, **Cv**: *Crassostrea virginica*, **Ct**: *Capitella teleta*, **Dc**: *Didymodactylos carnosus*, **Dgy**: *Dimorphilus gyrociliatus*, **Dp**: *Dreissena polymorpha*, **Ech**: *Elysia chlorotica*, **Em**: *Echinococcus multilocularis*, **Ema**: *Elysia marginata*, **Ga**: *Gigantopelta aegis*. **Hr**: *Helobdella robusta*, **Hrub**: *Haliotis rubra*, **Hruf**: *Haliotis rufescens*, **La**: *Lingula anatina*, **Lg**: *Lottia gigantea*, **Ls**: *Lamellibrachia satsuma*, **Lv**: *Lytechinus variegatus*, **Mco**: *Mytilus coruscus*, **Me**: *Mytilus edulis*, **Mg**: *Mytilus galloprovincialis*, **Ml**: *Macrostomum lignano*, **Mm**: *Mercenaria mercenaria*, **My**: *Mizuhopecten yessoensis*, **Ofu**: *Owenia fusiformis*, **Pa**: *Phoronis australis*, **Pca**: *Pomacea canaliculata*, **Pm**: *Pecten maximus*, **Pmi**: *Patiria miniata*, **Po**: *Plakobranchus ocellatus*, **Px**: *Protopolystoma xenopodis*, **Rm**: *Rotaria magnacalcarata*, **Rs**: *Rotaria socialis*, **Rso**: *Rotaria sordida*, **Rs1s**: *Rotaria sp*. *Silwood1*, **Rs2s**: *Rotaria sp*. *Silwood2*, **Sm**: *Schistosoma mansoni*, **Sme**: *Schmidtea mediterranea*, **Spur**: *Strongylocentrotus purpuratus*. **#**: 2DBD-NR. The capital letter (A, B or C) after # (2DBD-NR) indicates 2DBD-NR group, Arabic numeral after capital letter indicates individual gene, and a lowercase letter at the end of the gene indicates variant. *: All member of Rotifers 2DBD-NRA3 and 2DBD-NRA5 groups, **: All member of Echinodermata 2DBD-NRB group. GenBank Accession number of analyzed NRs see Table 6.

Phylogenetic analysis of identified 2DBD-NRs using amino acid sequence of both the first and the second DBD was carried out by Bayesian inference. The result shows that all of the 2DBD-NRs from protostome Spiralia and deuterostomes are clustered together forming a Spiralia/deuterostomes group (Bayesian posterior probabilities (BPPs) = 0.98), while all Ecdysozoa Nematoda 2DBD-NRs are clustered outside of the Spiralia/deuterostomes group. This result suggests that protostome Spiralia and deuterostomes share a close common ancestor 2DBD-NR gene (Fig 2). In protostome spiralia/deuterostomes group, 2DBD-NRs are clustered in two groups: 2DBD-NRA and 2DBD-NRB with BBP = 0.97 and 1, respectively (Fig 2). Both 2DBD-NRA and 2DBD-NRB groups contain members from protostome and deuterostome species, this result suggests that two ancient 2DBD-NR genes (*2DBD-NRA* and *2DBD-NRB*) were present in a common ancestor of protostomes and deuterostomes. Phylogenetic analysis further shows that most 2DBD-NR genes underwent duplications after the split of different animal phyla. Thus, 2DBD-NR in a certain animal phylum may be a paralogue, rather than an orthologue, of that in another animal phylum (Fig 2). For example, Rotifera 2DBD-NRs are clustered into five subgroups in the 2DBD-NRA group, and another four subgroups are clustered together without members from any other Phylum.

### 2. 2DBD-NRs in different animals

Members from both 2DBD-NRA and 2DBD-NRB groups are identified in Mollusca, Annelida and Brachiopoda; while 2DBD-NRA is not found in Phoronida and Echinodermata, 2DBD-NRB is not identified in Platyhelminths, Nemertea, Rotifera and Chordata. Since most 2DBD-NRs are paralogues among different animal phylum, we present our findings below by animal phyla.

#### 1) 2DBD-NRs in Mollusca

2DBD-NRs are identified in Mollusca species from Class Bivalvia and Class Gastropoda (Table 1). Phylogenetic analysis shows that Mollusca 2DBD-NRs are clustered in both 2DBD-NRA and 2DBD-NRB groups. 2DBD-NRA group contains only one member from each analyzed Mollusca species, it suggests that one 2DBD-NRA is present in Mollusca species from Class Bivalvia and Class Gastropoda. Mollusca 2DBD-NRB group contains three subgroups (2DBD-NRB1, 2DBD-NRB2 and 2DBD-NRB3), it suggests that three 2DBD-NRBs are present in analyzed Mollusca species (Fig 2). All of the three 2DBD-NRBs (2DBD-NRB1, 2DBD-NRB2 and 2DBD-NRB3) are found in species from Gastropoda, but only one 2DBD-NRB (2DBD-NRB1) is identified in species from Class Bivalvia. This result suggests that four 2DBD-NRs (2DBD-NRA, 2DBD-NRB1, 2DBD-NRB2 and2DBD-NRB3) are present in Gastropoda and two 2DBD-NRs (2DBD-NRA and 2DBD-NRB1) are present in Bivalvia. Since Mollusca 2DBD-NRB2 and 2DBD-NRB3 subgroups share a shallower node, it suggests that 2DBD-NRB2 and 2DBD-NRB3 were formed by recent gene duplication (Fig 2 and Table 1).

**Table 1:** 2DBD-NRs identified in Mollusca.

Class	Species	2DBD-NRA	2DBD-NRB1	2DBD-NRB2	2DBD-NRB3	Total
Bivalvia	*Crassostrea virginica*, *C*. *gigas*	1	1			2
	*Pecten maximus*	1	1			2
	*Mytilus coruscus*, *M*. *edulis*, *M*. *galloprovincialis*	1	1			2
	*Dreissena polymorpha*	1	1			2
	*Mercenaria mercenaria*	1	1			2
	*Mizuhopecten yessoensis*	1	1			2
Gastropoda	*Batillaria attramentaria*	1	1			2
	*Lottia gigantea*	1	1			2
	*Gigantopelta aegis*	1	1			2
	*Haliotis rufescens*	1	1	1		3
	*Haliotis rubra*	1		1		2
	*Plakobranchus ocellatus*	1		1	1	3
	*Aplysia californica*	1		1	1	3
	*Pomacea canaliculata*	1		1	1	3
	*Elysia chlorotica*, *E*. *marginata*,	1			1	2
	*Biomphalaria glabrata*	1			1	2
	*Bulinus truncatus*	1			1	2
	*Candidula unifasciata*	1				1

Mining genome database of Mollusca *Crassostrea gigas*, Vogeler et al. [23] identified two *C*. *gigas* 2DBD-NRs (Cg2DBDγ and Cg2DBDδ). They showed that Cg2DBDγ contained the same P-box sequences, CEACKK, as *S*. *mansoni* 2DBD-NRs in the first DBD, but Cg2DBDδ contained a different P-box sequence, CLPCKS, in the first DBD, this P-box sequence was not found in any other nuclear receptor. Amino acid sequence alignment shows that 2DBD-NRBs from Annelida, Brachiopoda and Phoronida and Mollusca possess the same P-box sequence CLPCKS in their first DBD with few members demonstrating divergent sequences (S1 File).

#### 2) 2DBD-NRs in Brachiopoda

Three members were identified in Brachiopoda *Lingula anatine*. MrBayes inference shows that two of them belong to 2DBD-NRA group (La2DBD-NRA1 and La2DBD-NRA2) and one is clustered in 2DBD-NRB group (La2DBD-NRB) (Fig 2).

#### 3) 2DBD-NRs in Phoronida

One member is identified in Phoronida *Phoronis australis* that belongs to 2DBD-NRB group (Pa2DBD-NRB) (Fig 2).

#### 4) 2DBD-NRs in Nemertea

One member is identified in Nemertea *Notospermus geniculatus* and it is clustered in 2DBD-NRA group (Ng2DBD-NRA) (Fig 2).

#### 5) 2DBD-NRs in Annelida

2BDB-NRs are identified in Annelida species from Class Polychaeta and Class Clitellata, they are clustered in five subgroups. Three subgroups are clustered in 2DBD-NRA group and two subgroups are in the 2DBD-NRB group. This result suggests that at least five 2DBD-NRs exist in Annelida. 2DBD-NRA2 and 2DBD-NRB2 are identified in both Class Polychaeta and Clitellata, 2DBD-NRA1 and 2DBD-NRB1 are identified only in Class Polychaeta, while 2DBD-NRA3 is only identified in Class Clitellata (Fig 2, Table 2).

**Table 2:** 2DBD-NRs in Annelida.

Class	Species	2DBD-NRA1	2DBD-NRA2	2DBD-NRA3	2DBD-NRB1	2DBD-NRB2	Total
Polychaeta	*Owenia fusiformis*	1				1	2
	*Capitella teleta*	1	1		1	1	4
	*Lamellibrachia satsuma*	1			1	2	4
	*Dimorphilus gyrociliatus*	1					1
Clitellata	Helobdella robusta		1	3		1	5

#### 6) 2DBD-NRs in Rotifera

NRs were found genome-wide in Rotifera in different species of *Brachionus*, no 2DBD-NR were identified from Rotifera in a previous study [29]. In this study, 2DBD-NRs were identified in ten species from two subclasses (Bdelloidea and Monogononta) of Class Eurotatoria (Table 3) in Rotifera. These 2DBD-NRs are clustered in five subgroups in 2DBD-NRA group. This result suggests that five 2DBD-NRAs are present in Rotifera Eurotatoria Class. 2DBD-NRA1 are identified in both subclasses, 2DBD-NRA2 and 2DBD-NRA3 are only identified in subclass Bdelloidea, while 2DBD-NRA4 and 2DBD-NRA5 are only identified in subclass Monogononta. The significant character of Rotifera 2DBD-NRs is that two or more divergent copies of every 2DBD-NR gene are present, this is consistent with the previous study of the genome of Bdelloidea [30, 31] (Fig 2 and Table 3).

**Table 3:** 2DBD-NRs in Rotifera.

Class	Subclass	Species	2DBD-NRA1	2DBD-NRA2	2DBD-NRA3	2DBD-NRA4	2DBD-NRA5	Total
Eurotatoria	Bdelloidea	*Rotaria magnacalcarata*	1	2	2			5
		*Adineta steineri*	2	1	2			5
		*Adineta ricciae*	2	2	2			6
		*Rotaria sp*. *Silwood1*	1	3	2			6
		*Rotaria sp*. *Silwood2*		2	3			5
		*Rotaria sordida*	1	2	2			5
		*Rotaria socialis*	1	2	2			5
		*Didymodactylos carnosus*		2	2			4
	Monogononta	*Brachionus calyciflorus*	2			2	1	5
		*Brachionus plicatilis*	1			1	1	3

#### 7) 2DBD-NRs in Platyhelminths

Our previous study showed that two 2DBD-NRs were present in Rhabditophora *Macrostomum lignano* [16, 21] and four in *Schmidtea mediterranea*. Three members were present in parasitic species from Platyhelminths including Class Monogenea, Cestoda and Trematoda, respectively [12, 14, 15, 17–21]. Phylogenetic analysis of Platyhelminths 2DBD-NRs in this study shows that all Platyhelminths 2DBD-NRs are clustered in 2DBD-NRA group, which suggests that 2DBD-NRB gene is missing in Platyhelminths. All parasitic 2DBD-NRs are clustered in three subgroups: 2DBD-NRA1 (*Schistosoma mansoni* 2DBD-NRγ orthologues), 2DBD-NRA2 (*S*. *mansoni* 2DBD-NRα orthologues) and 2DBD-NRA3 (*S*. *mansoni* 2DBD-NRβ orthologues), each subgroup contains members from species of each Class of Monogenea, Cestoda and Trematoda. For free-living flatworms, both *M*. *lignano* 2DBD-NRs are clustered in Platyhelminths 2DBD-NRA1 subgroup. For the four *S*. *mediterranea* 2DBD-NRs, one is clustered in Platyhelminths 2DBD-NRA1 subgroup, one is in Platyhelminths 2DBD-NRA2 subgroup and one is clustered in Platyhelminths 2DBD-NRA3 subgroup. The fourth *S*. *mediterranea* 2DBD-NR (Sme2DBD-NRA4) is on the base of 2DBD-NRA2 and 2DBD-NRA3 subgroups (Fig 2). This result suggests that 2DBD-NR underwent another round of duplication in a common ancestor of *S*. *mediterranea* and parasitic Platyhelminths and then one 2DBD-NR was lost in a common ancestor of parasitic Platyhelminths. This result is consistent with our previous study [21]. In this study, 2DBD-NRs are identified in more parasitic Platyhelminths including the lung flukes *Paragonimus westermani*, *P*. *skrjabini miyazakii* and *P*. *heterotremus*, liver fluke *Fasciola gigantica*, giant intestinal fluke *Fasciolopsis buski* and tapeworm *Sparganum proliferum*. Sequence alignment shows that all of them are highly conserved with known Platyhelminths 2DBD-NRs.

#### 8) 2DBD-NRs in Echinodermata

One member was identified in each analyzed species of Echinodermata including those from Class Asteroidea, Class Echinoidea and Class Crinoidea. MrBayes inference analysis shows that all Echinodermata 2DBD-NRs form a monophyletic subgroup in 2DBD-NRB group (Fig 2 and Table 4). Previously, 33 NRs were identified in the genome database of *Strongylocentrotus purpuratus*, but until this report no 2DBD-NR was reported [32].

**Table 4:** 2DBD-NRs in deuterostome.

Phyla	Class	Species	2DBD-NRA	2DBD-NRB
Echinodermata	Asteroidea	*Acanthaster planci*		1
		*Asterias rubens*		1
		*Patiria miniata*		1
	Echinoidea	*Lytechinus variegatus*		1
		*Strongylocentrotus purpuratus*		1
	Crinoidea	*Anneissia japonica*		1
Chordata	Leptocardii	*Branchiostoma belcheri*	1	
		*Branchiostoma floridae*	1	
		*Branchiostoma lanceolatum*	1	

#### 9) 2DBD-NRs in Chordata

One member is identified in each species of analyzed Chordata Amphioxi including *Branchiostoma belcheri*, *B*. *floridae* and *B*. *lanceolatum*. MrBayes inference analysis shows that all of them form a monophyletic subgroup in 2DBD-NRA group (Fig 2 and Table 4). Though 33 NRs were identified in *B*. *floridae* previously, but until this report no 2DBD-NR was reported in Amphioxi [33].

#### 10) 2DBD-NRs in Nematoda

2DBD-NRs are present in nematode species *Aphelenchus avenae*, *Caenorhabditis brenneri* and *Caenorhabditis remanei*. Four 2DBD-NRs are found in *C*. *remanei*, one in *C*. *brenneri* and seven in *A*. *avenae*. Phylogenetic analysis shows that all these 2DBD-NRs are clustered outside of Spiralia/deuterostomes 2DBD-NR group (Fig 2). Sequence alignment of DBDs shows that the P-box sequence is highly divergent in Nematoda 2DBD-NRs (S1 File). These results suggests that Nematode 2DBD-NRs underwent extensive divergence as found in other nematode NRs [34–37].

See Table 5 for a summary of the numbers of 2DBD-NRs identified in different animal species.

**Table 5:** Numbers of 2DBD-NRs identified in different animals.

Animal	Class	2DBD-NRA	2DBD-NRB	Total
Rotifera	*Eurotatoria*	5		5
Mollusca	*Bivalvia*	1	1	2
	*Gastropoda*	1	3	4
Annelida	*Clitellata*	3	1	4
	*Polychaeta*	2	3	5
Platyhelminthes	Rhabditophora	4		4
	Monogenea	3		3
	Cestoda	3		3
	Trematoda	3		3
Brachiopoda	Lingulata	2	1	3
Phoronida			1	1
Nemertea	Pilidiophora	1		1
Echinodermata	Asteroidea		1	1
	Echinoidea		1	1
	Crinoidea		1	1
Chordata	Leptocardii	1		1

### 3. Amino acid sequence analysis of 2DBD-NRs

#### 1) AB domain

**Fig 3:**
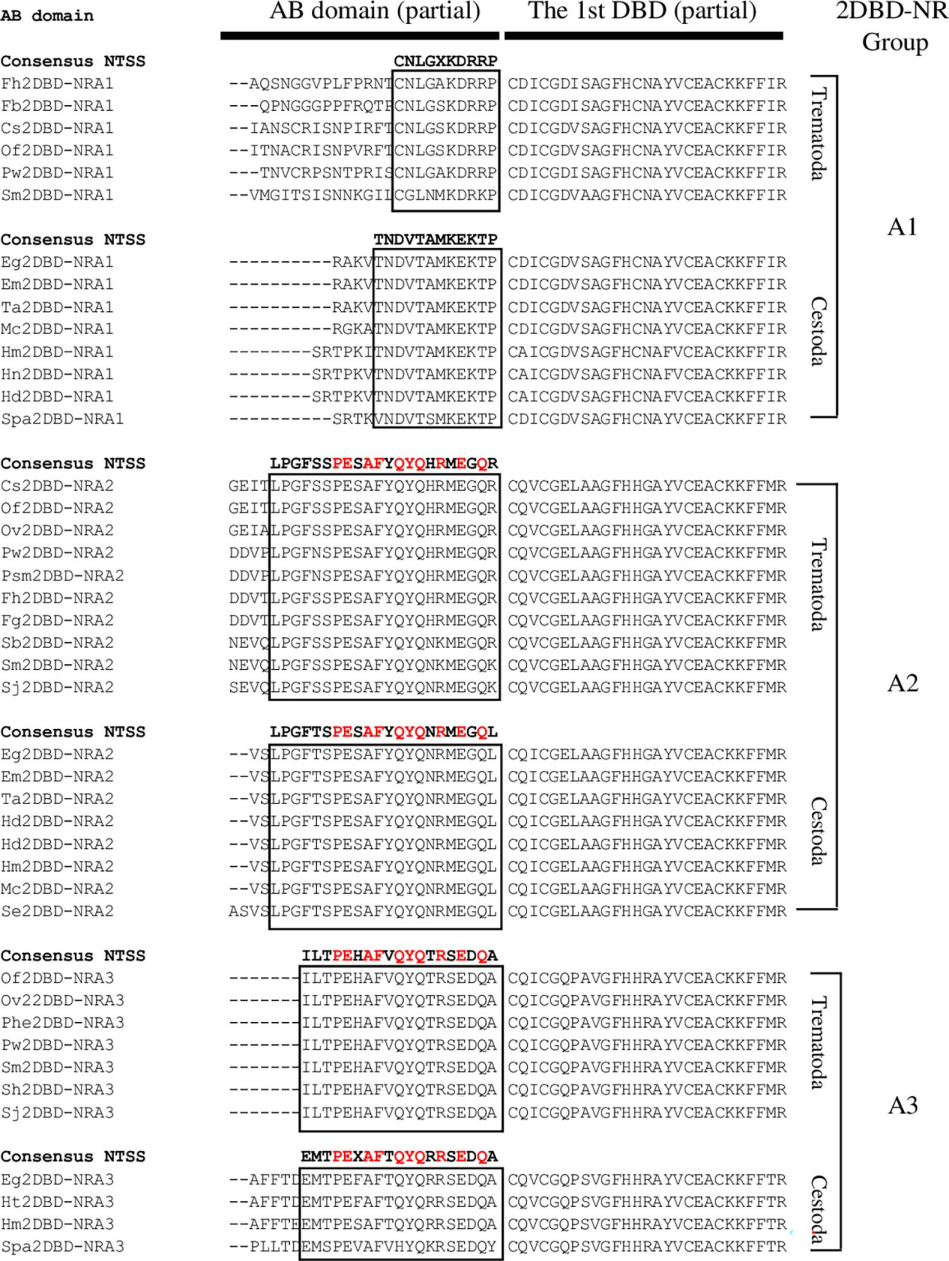
Amino acid sequence alignment shows the conserved NTSS in parasitic Platyhelminths 2DBD-NRs. **Cs**: *Clonorchis sinensis*, **Eg**: *Echinococcus granulosus*, **Em**: *Echinococcus multilocularis*, **Fb**: *Fasciolopsis buski*, **Fh**: *Fasciola hepatica*, **Fg**: *Fasciola gigantica*, **Hd**: *Hymenolepis diminuta*, **Hm**: *Hymenolepis microstoma*, **Hn**: *Hymenolepis nana*, **Ht**: *Hydatigera taeniaeformis*, **Of**: *Opisthorchis felineus*, **Ov**: *Opisthorchis viverrini*, **Phe**: *Paragonimus heterotremus*, **Mc**: *Mesocestoides corti*, **Pw**: *Paragonimus westermani*, **Psm**: *Paragonimus skrjabini miyazakii*, **Se**: *Spirometra erinaceieuropaei*, **Sb**: *Schistosoma bovis*, **Sh**: *Schistosoma haematobium*, **Sj**: *Schistosoma japonicum*, **Spa**: *Sparganum proliferum*, **Sm**: *Schistosoma mansoni*, **Ta**: *Taenia asiatica*. The red letters indicate the amino acid residues that are conserved in parasitic Platyhelminths 2DBD-NRA2 and 2DBD-NRA3, it suggests that parasitic Platyhelminths 2DBD-NRA2 and 2DBD-NRA3 underwent recent duplication.

A highly conserved ’N-terminal signature sequence’ (NTSS) [38] is found at the 3’end of the A/B domain of parasitic Platyhelminths 2DBD-NRs. A NTSS of CNLGXKDRRP is present in Platyhelminth Trematode 2DBD-NRA1s, and a NTSS of TNDVTAMKEKTP is present in Cestoda 2DBD-NRA1s. A NTSS of (S/T)PEXAFXQYQXR(M/S)EGQX represents both Platyhelminths 2DBD-NRA2s and 2DBD-NRA3s (Fig 3). The fact that Platyhelminth 2DBD-NRA2 and 2DBD-NRA3 members share a conserved NTSS further supports our phylogenetic analysis.

#### 2) DBDs

DBD is the most conserved region in NRs, this region contains two highly conserved C4 type zinc fingers with a module of C-X2-C-X13-C-X2-C for the first Zinc finger (CI) and C-X5-C-X9-C-X2-C for the second Zinc finger (CII), where C represents cysteine, X represents any variable and the next number indicates the number of amino acids between the cysteines [39]. Amino acid alignment of all identified 2DBD-NRs in this study shows that both DBDs of most of the 2DBD-NRs contain the conserved zinc finger modules as above with very little exception (Fig 1B and S2 File). Recently, we reported a novel zinc finger CHC2 motif (C-X6-C-X9-H-X2-C) in DBD of parasitic Platyhelminth NRs [21], this motif is not identified in any 2DBD-NRs.

**Fig 4:**
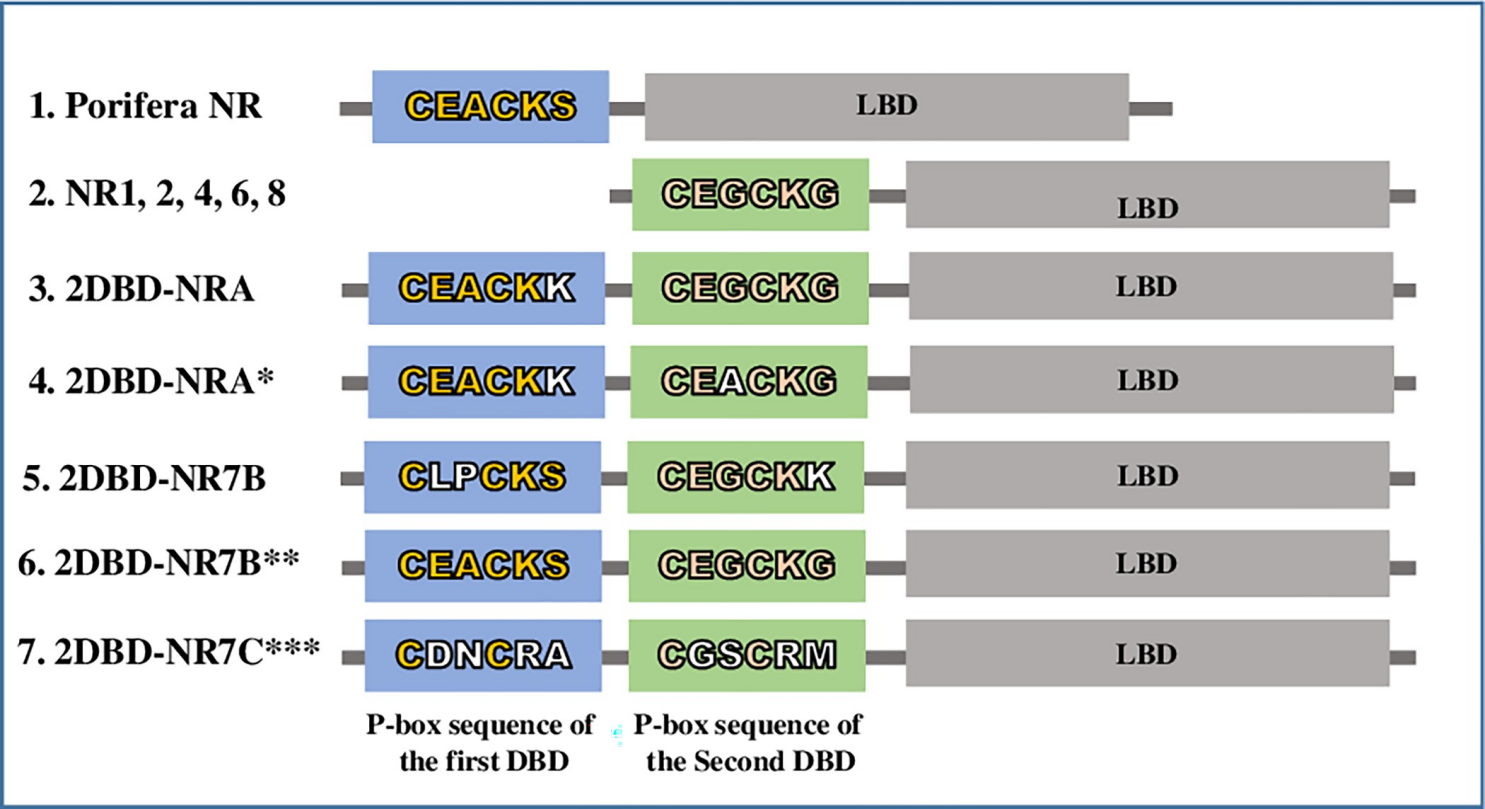
P-P module of 2DBD-NRs (the P-box sequence in the first DBD and the second DBD of 2DBD-NRs). 1. Shows the P-box sequence of a Porifera NRs (*Suberites domuncula* RXR, SdRXR, GenBank: CAD57002.1) that is the same as the first DBD of Echinodermata 2DBD-NRB. 2. Shows the P-box sequence of NRs in subfamily 1, 2 4, 6 and 8 that is the same as the P-box of the second DBD of 2DBD-BRA. 3. P-P module of 2DBD-NRA (CEACKK-CEGCKG) which is found in most of 2DBD-NRA except members of Rotifers NR7A3 and NR7A5 groups. 4. P-P module of 2DBD-NRA (CEACKK-CEACKG) which is only found in the members of Rotifers NR7A3 and NR7A5 groups. 5. P-P module of 2DBD-NRB (CLPCKS-CEGCKK) which is found in most of 2DBD-NRB except members of Echinodermata 2DBD-NRB. 6. P-P module of 2DBD-NRB (CEACKS-CEGCKG) which is only found in Echinodermata 2DBD-NRB. 7. P-P module of the member of 2DBD-NRC group is highly variable. An example (Cr2DBD-NRC1) is shown in the picture. *: P-P module sequences only found in the members of Rotifers 2DBD-NRA3 and 2DBD-NRA5. **: P-P module sequences only found in the members of Echinodermata 2DBD-NRB. ***: P-P module of the member of 2DBD-NRC group is highly variable, here is only an example (Cr2DBD-NRC1). Letters in yellow color in the P-box sequence indicate the conserved amino acids and the letters in white color in the P-box sequence indicate the divergent amino acids.

In 1989, [40] identified two conserved motifs in DBD of NRs. One motif was defined as a proximal box (P-box) that follows the third Cysteine of zinc finger I (CI) including five amino acids. The other motif was a distal box (D-box) which was located between the fifth and sixth Cysteine in zinc finger II (CII) of DBD (Fig 1B). The P-box is critical for identifying the primary nucleotide sequence of the half-sites and the D-box is important for protein dimerization. Previously, we demonstrated that 2DBD-NRs possess a P-box sequence of EACKK in the first DBD, which is similar but different from the P-box of ERRs (EACKA). The P-box sequence of the second DBD (EGCKG) followed by the amino acid sequence FFRR (EGCKGFFRR) is identical to that of most members in NR subfamily 1 (NR1) [12, 14]. Recently, Vogeler et al. [23] showed that a different P-box sequence (LPCKS) was present in the first DBD of a 2DBD-NR in Mollusca *C*. *gigas* [23]. In this study, various different P-box sequences are found in 2DBD-NRs. The P-box sequence in the first DBD and the second DBD of 2DBD-NRs (**P-P module**) was found to be different among 2DBD-NRAs, 2DBD-NRBs and 2DBD-NRCs (Fig 4 and S1 and S2 Files). The P-P module of 2DBD-NRA is EACKK-EGCKG (Fig 4(3) and 4(4)) with a few divergence sequences in the second DBD (S1 and S2 Files). Two types of P-P modules are found in 2DBD-NRB, one is LPCKS-EGCKK (Fig 4(5), and it is present in 2DBD-NRBs of Mollusca, Annelida, Brachiopoda and Phoronida, with a divergent sequence in the P-box of the first DBD in Annelida and Brachiopoda 2DBD-NRB (S1 and S2 Files). The P-box sequence of LPCKS was first identified in Cg2DBDδ [23]. The P-P module EACKS-EGCKG is only found in Echinodermata 2DBD-NRBs (Fig 4(6)). Interestingly, the P-box sequence of the first DBD of Echinodermata 2DBD-NRBs (EACKS) is identical to that of basal metazoans Porifera NRs (SdRXR and RsNR1) (Fig 4(1)) and the P-box sequence of the second DBD is identical to that of most members of 2DBD-NRA, and some members from NR subfamily 1, 2, 4, 6 and 8 (Fig 4(2)). The P-P module of 2DBD-NRCs is highly variable, not only from 2DBD-NRA and 2DBD-NRB, but also variable from the members in 2DBD-NRC group (Fig 4(7), S1 and S2 Files). The different P-P module among 2DBD-NRs suggests that the mechanism of DNA binding may be different in 2DBD-NRAs, 2DBD-NRBs and 2DBD-NRCs and it suggests that 2DBD-NRAs, 2DBD-NRBs or 2DBD-NRCs recognize and regulate different kinds of target genes.

The D-box of NR is located between the fifth and sixth Cysteine in zinc finger II (CII) of DBD with five amino acid residue between the two Cysteines (C-X5-C) [40]. Amino acid sequence alignment of 2DBD-NRs shows that most of 2DBD-NRs have a conserved D box with C-X5-C in both DBDs (S2 File).

#### 3) Amino acid sequence between the first and second DBDs

**Fig 5:**
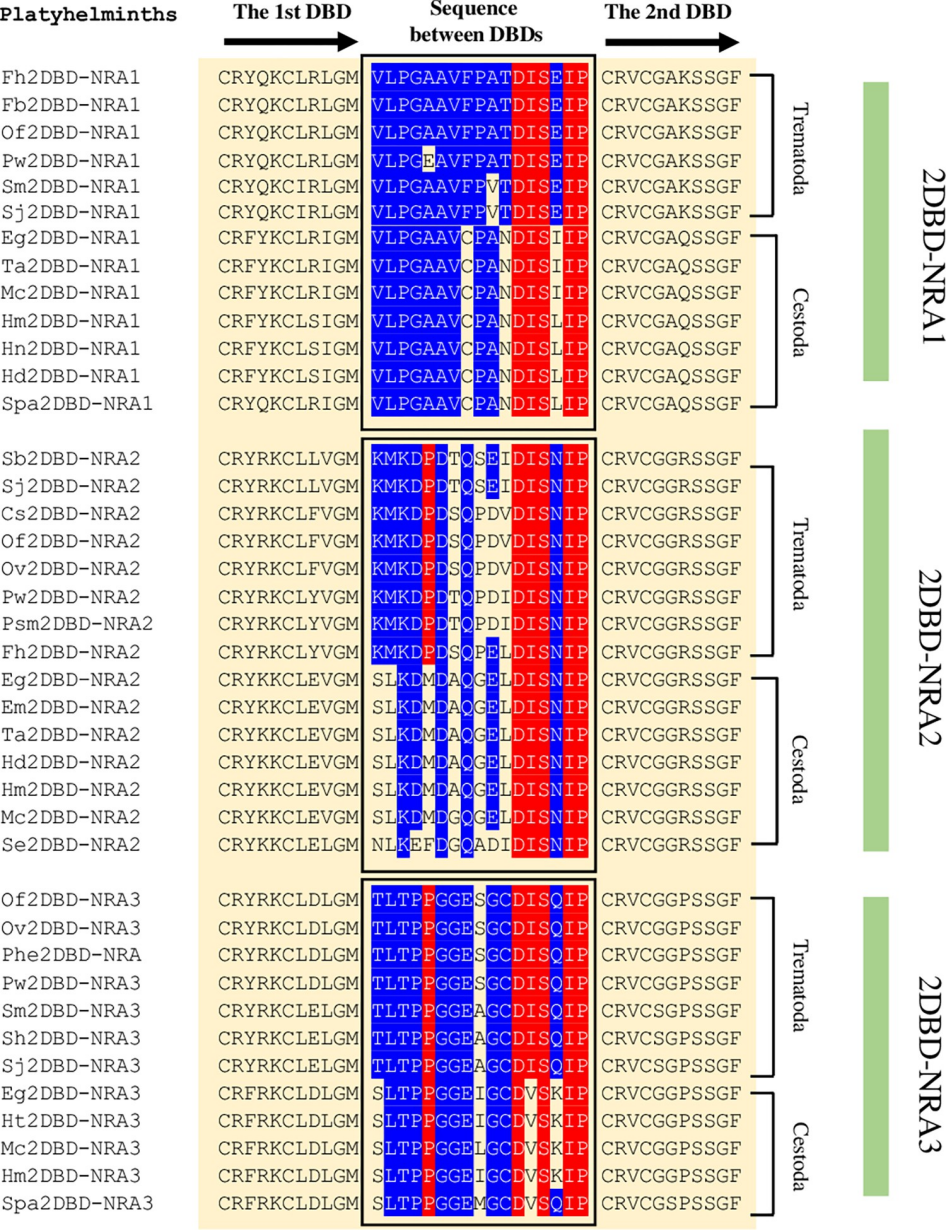
Amino acid sequence between the first and second DBDs in Platyhelminth 2DBD-NRs. Cs: *Clonorchis sinensis*, Eg: *Echinococcus granulosus*, Em: *Echinococcus multilocularis*, Fb: *Fasciolopsis buski*, Fh: *Fasciola hepatica*, Hd: *Hymenolepis diminuta*, Hm: *Hymenolepis microstoma*, Hn: *Hymenolepis nana*, Ht: *Hydatigera taeniaeformis*, Mc: *Mesocestoides corti*, Of: *Opisthorchis felineus*, Ov: *Opisthorchis viverrini*, Pw: *Paragonimus westermani*, Psm: *Paragonimus skrjabini miyazakii*, Sj: *Schistosoma japonicum*, Se: *Spirometra erinaceieuropaei*, Sh: *Schistosoma haematobium*, Sm: *Schistosoma mansoni*, Spa: *Sparganum proliferum*, Ta: *Taenia asiatica*. Blue highlighted letters indicate the conserved amino acid residues in each subgroup and red highlighted letters indicate the conserved amino acid residues among subgroups.

**Fig 6:**
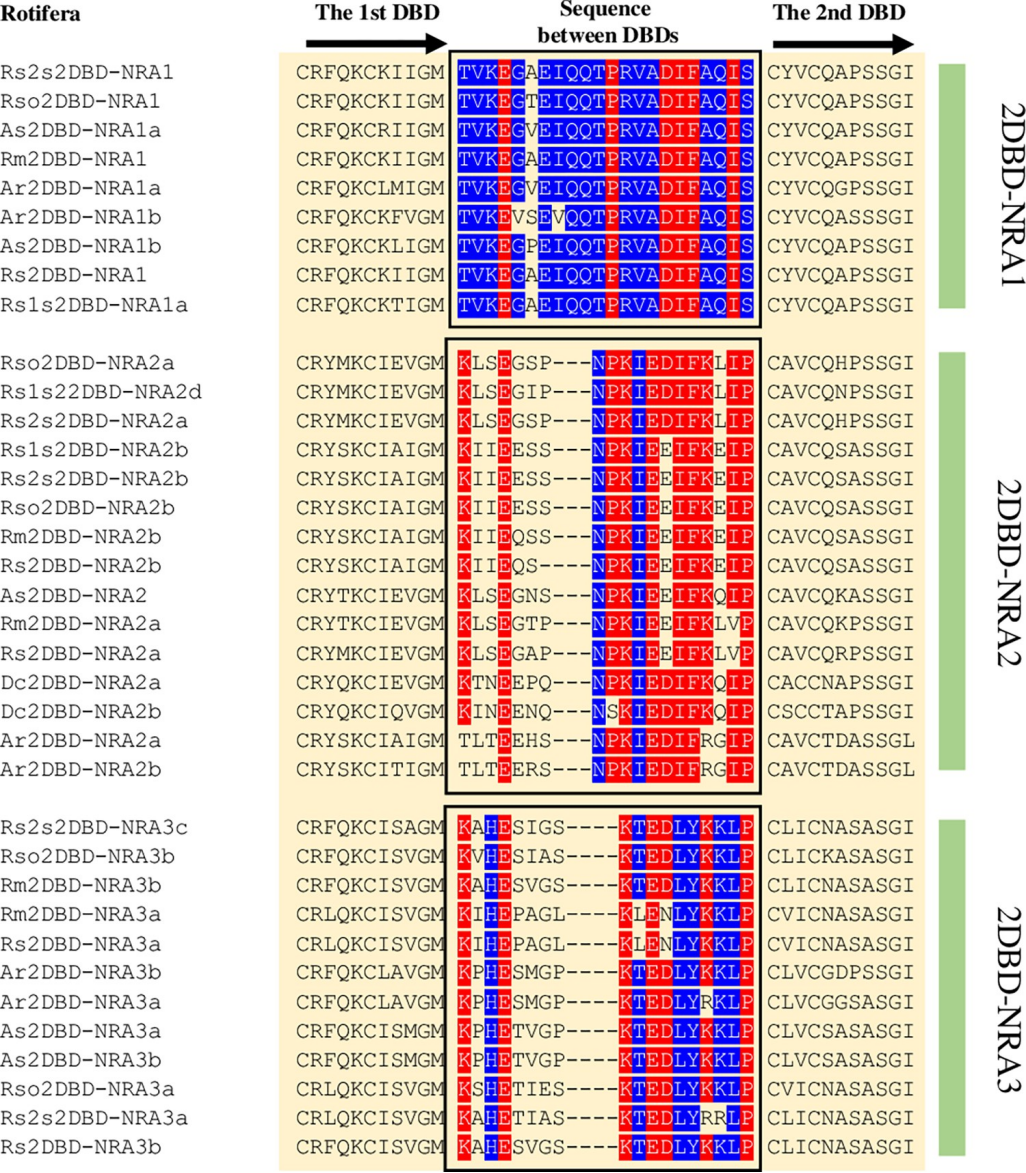
Amino acid sequence between the first and second DBDs in Rotifera 2DBD-NRs. Ar: *Adineta ricciae*, As: *Adineta steineri*, *Rotaria magnacalcarata*, Dc: *Didymodactylos carnosus*, Rm: *Rotaria magnacalcarata*, Rs: *Rotaria socialis*, Rs1s: *Rotaria sp*. *Silwood1*, Rs2s: *Rotaria sp*. *Silwood2*, Rso: *Rotaria sordida*, Rs1s: *Rotaria sp*. *Silwood1*. Blue highlighted letters indicate the conserved amino acid residues in each subgroup and red highlighted letters indicate the conserved amino acid residues among subgroups.

**Fig 7:**
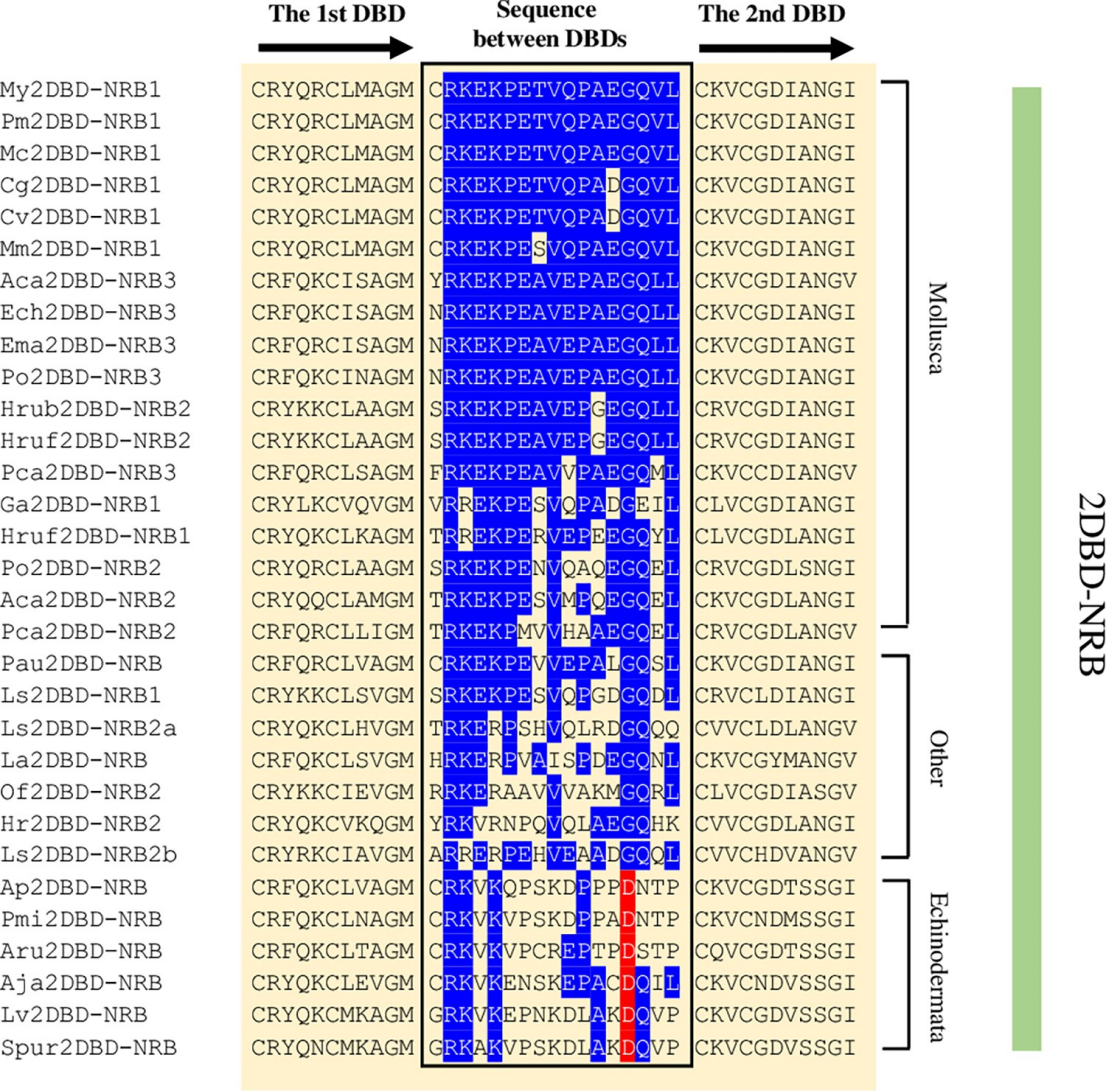
Amino acid sequence between the first and second DBDs in 2DBD-NRBs. Aca: *Aplysia californica*, Aj: *Anneissia japonica*, Ap: *Acanthaster planci*, Aru: *Asterias rubens*, Cg: *Crassostrea gigas*, Cv: *Crassostrea virginica*, Ech: *Elysia chlorotica*, Ema: *Elysia marginata*, Hr: *Helobdella robusta*, Hrub: *Haliotis rubra*, Hruf: *Haliotis rufescens*, La: *Lingula anatina*, Ls: *Lamellibrachia satsuma*, Lv: *Lytechinus variegatus*, Mco: *Mytilus coruscus*, Mm: *Mercenaria mercenaria*, My: *Mizuhopecten yessoensis*, Ofu: *Owenia fusiformis*, Pa: *Phoronis australis*, Pca: *Pomacea canaliculata*, Pm: *Pecten maximus*, Pmi: *Patiria miniata*, Po: *Plakobranchus ocellatus*, Spur: *Strongylocentrotus purpuratus*. Blue highlighted letters indicate the conserved amino acid residues in members of 2DBD-NRB group and red highlighted letters indicate the conserved amino acid residues in Echinodermata 2DBD-NRBs.

Amino acid sequence alignment shows that most members of the 2DBD-NRA and 2DBD-NRB possess 17–22 amino acids between two DBDs. Highly conserved sequences in this region were found in parasitic Platyhelminths 2DBD-NRAs (Fig 5), Rotifera 2DBD-NRAs (Fig 6) and in 2DBD-NRBs (Fig 7), respectively.

#### 4) The C-terminal Extension (CTE)

**Fig 8:**
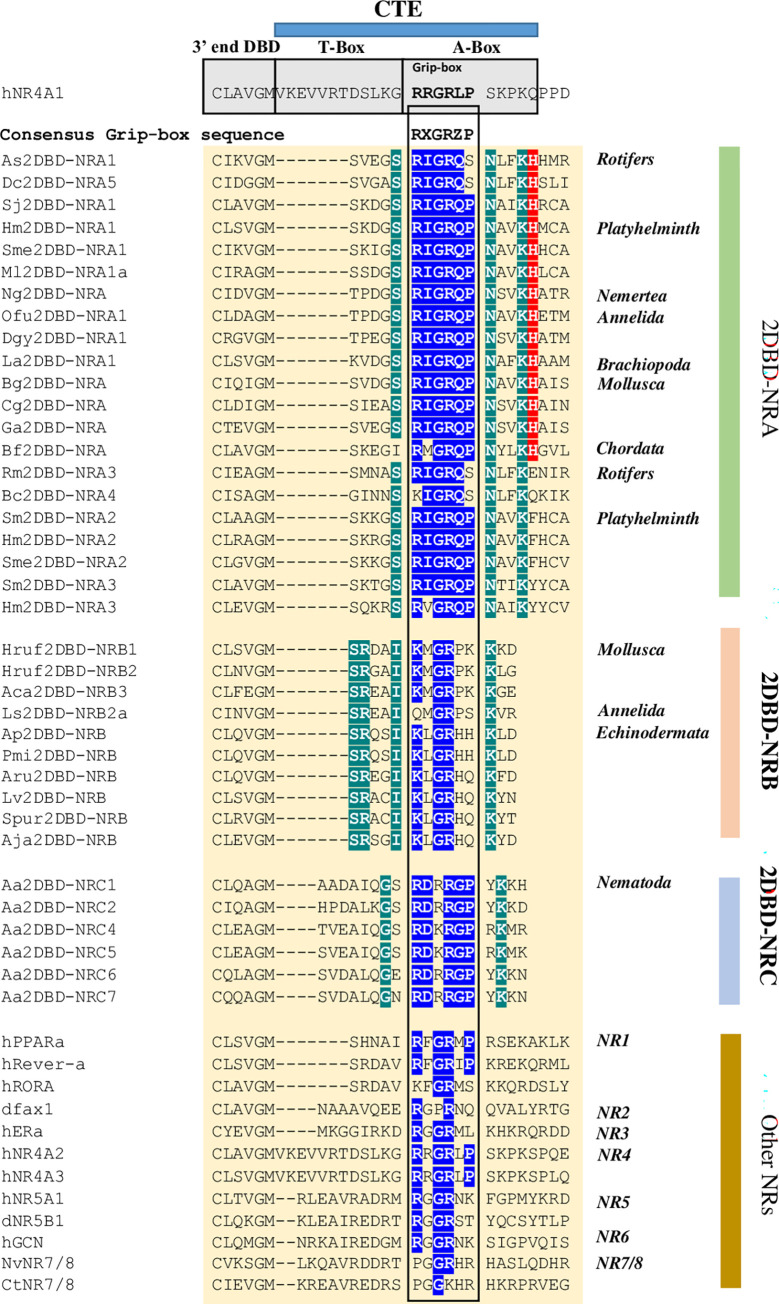
Amino acid sequence alignment of the C-terminal Extension (CTE) of 2DBD-NRs. Amino acid sequence alignment shows conserved G-box sequence in 2DBD-NRs. The deep green highlighted letters indicate 2DBD-NR gene specific amino acid residues in CTE, the blue highlighted letters indicate conserved amino acid residues in the G-box of 2DBD-NRs and other NRs, the red highlighted letters indicate conserved amino acid residues amino acid (H) after the G-box. They are ancient signal amino acids among 2DBD-NRAs.

The C-terminal Extension (CTE) of DBD is important for DNA sequence recognition and binding. In 1992, two boxes in CTE of human NGFI-B (hNR4A1) were identified [41]. One box, termed T-Box, consisted of 12 amino acids that determined binding to tandem repeats of the half-site. The adjacent C-terminal seven amino acids, termed A-box, was required for recognition of the DNA binding element [41]. In 1998, a subunit, termed Grip Box (G-box)in the CTE of human RevErbA-α was identified [42]. The G-box formed a significant minor groove in the DNA binding surface [42]. They further showed that the G-box sequence was conserved in different orphan receptors despite the length of the pre-Grip sequences was different. The consensus G-box sequence they identified as RXGRZP (where X is a F, R, or G and Z usually contains a hydrophobic side chain). In this study, amino sequence alignment showed that all 2DBD-NRs contain a conserved G-box with the consensus sequence of RXGRQ(P/S) in 2DBD-NRAs, KXGR(P/H) in 2DBD-NRBs and RDRRGP in Nematoda *A*. *avenae* 2DBD-NRCs (Fig 8). The different conserved G-box sequence among 2DBD-NRA, 2DBD-NRB and 2DBD-NRC may represent the different DNA binding ability of these NRs.

Amino acid sequence alignment shows that the fifth amino acid (H) after the G-box are conserved in most 2DBD-NRAs including members from Nemertea, Annelida, Brachiopoda, Mollusca and Chordata, members from Rotifer 2DBD-NRA1 and 2DBD-NRA2 subgroup and members from Platyhelminthes 2DBD-NRA1 subgroup. However, the members from Rotifer 2DBD-NRA3 and 2DBD-NRA4 subgroups and the members from Platyhelminth 2DBD-NRA2 and 2DBD-NRA3 subgroups possess a different amino acid at this position. This suggests that the fifth amino acid residue (H) after the G-box are ancient signal amino acids among 2DBD-NRAs. The G-box sequence is also conserved in human NGFI-B that is localized in the 5’ end of the A-box of NGFI-B (Fig 8), the pre-Grip sequences represents the T-box of NGFI-B which contains 12 residues [41]. The pre-Grip sequences in 2DBD-NR only contains five amino acids in 2DBD-NRA and 2DBD-NRBs, and eight amino acids in 2DBD-NRC, this data suggests that no conserved T-box is present in 2DBD-NRs.

#### 5) LBD

**Fig 9:**
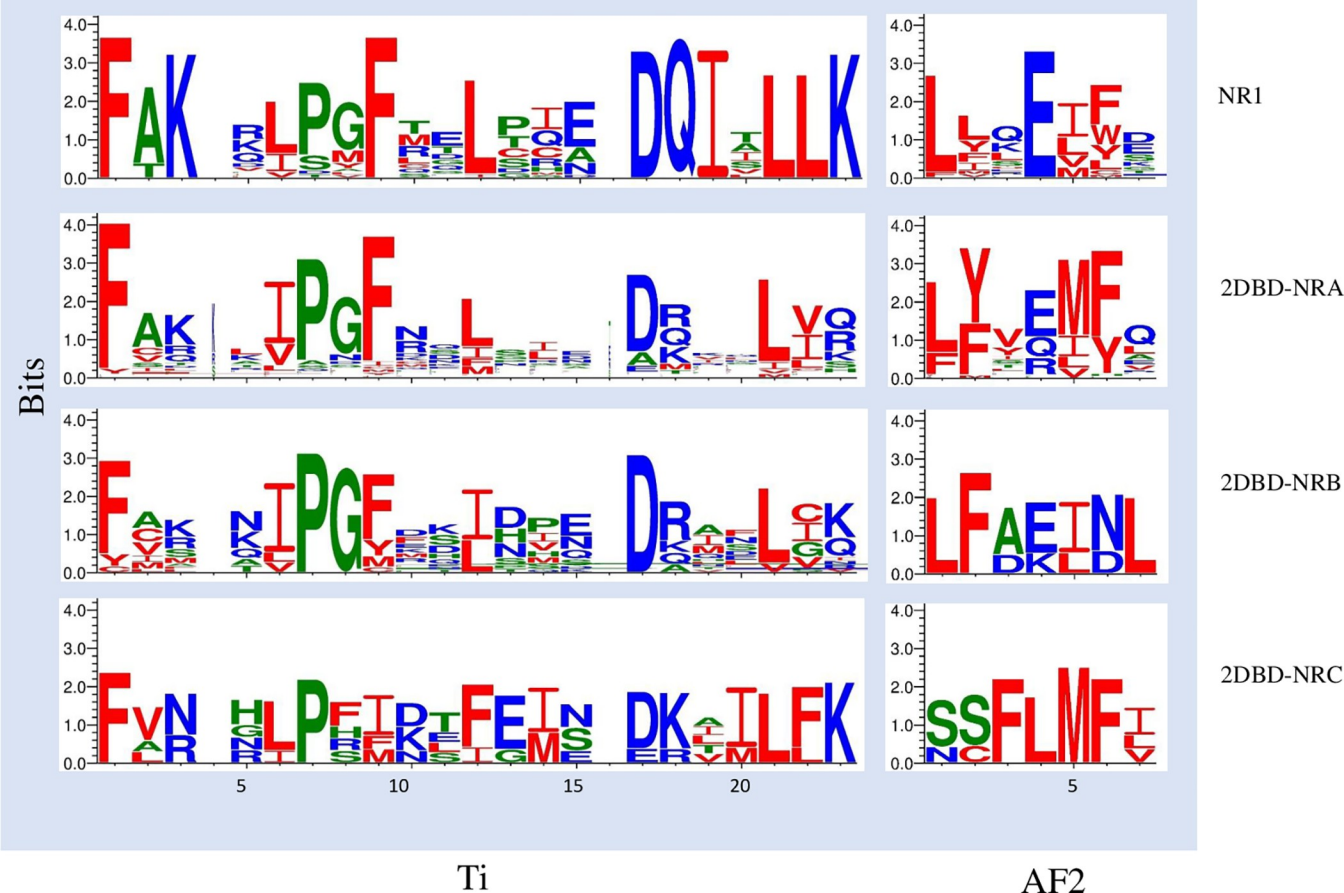
Conserved amino acid sequence of LBD specific signature (Ti) and activation function TAF-2 (AF2) in 2DBD-NRs. Sequence logo shows that both Ti and AF2 are conserved in 2DBD-NRs, and the pattern is similar to that of NR subfamily 1 (NR1). For the sequences used for generation of the logo see S3 and S4 Files. Blue letter indicates a hydrophilic residue (RKDENQ), green letter indicates a neutral residue (SGHTAP) and red letter indicates a Hydrophobic residue (YVMCLFIW).

LBD of NRs contain 12 helices and has two conserved regions, one region is known as “signature”, it contains 34 amino acid residues between the C terminus of H3 and the middle of H5. Within this region, a motif containing 20 amino acid residues was defined as an LBD specific signature (Ti) for the NR superfamily, it’s consensus sequence is ((F,W,Y)(A,S,I) (K,R,E,G)xxxx(F,L)xx(L,V,I)xxx(D,S)(Q,K)xx(L,V)(L,I,F)) [43]. In the C-terminus of LBD, there is an inducible transcription activation function TAF-2 (AF2) [44, 45]. The amino sequence of AF-2 region was conserved among many nuclear hormone receptors, the common consensus AF2-AD core structure is ФФxEФФ, where Ф denotes a hydrophobic residue [46–48]. Amino acid sequence alignment of 2DBD-NRs shows that Ti is conserved in 2DBD-NR (Fig 9 and S3 File). AF2-AD core sequence is identified in most of 2DBD-NRs, but it is missing in the nematode *Aphelenchus avenae* 2DBD-NRs. Sequence alignment shows that AF2-AD core sequence is conserved among 2DBD-NRs with ФФx(E,Q,R)ФФ in 2DBD-NRAs, ФФx(E,K)Фh (where h denotes a Hydrophilic residue) in 2DBD-NRBs and xxФФФФ in *Caenorhabditis brenneri* 2DBD-NRCs (Fig 9 and S4 File).

**Fig 10:**
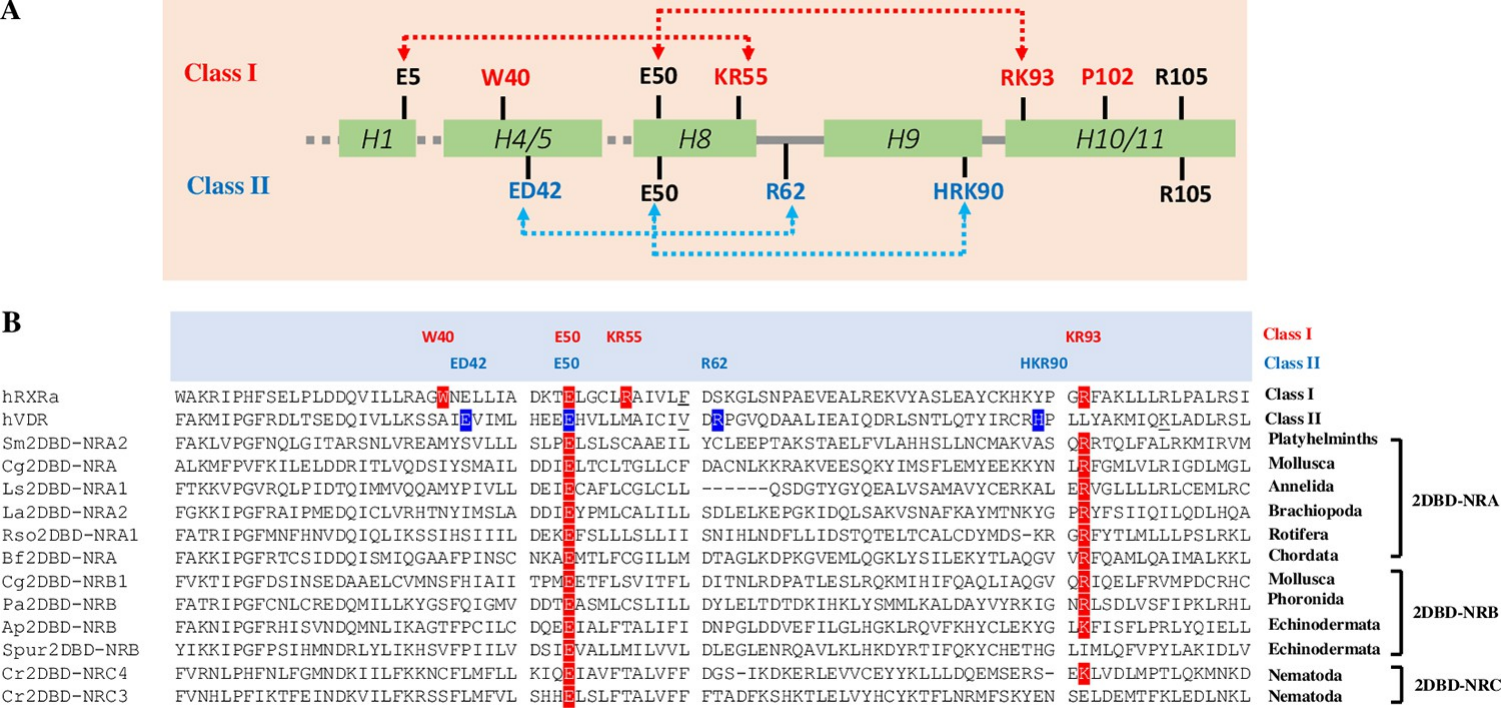
Identification of amino acids in LBD of 2DBD-NRs that are conserved in class I NRs. **A.** Secondary structure diagram showing NR class-specific features [50]. The red letters indicate conserved amino acids in class I NRs, the blue letters indicate conserved amino acids in class II NRs, and the black letters indicate no class-specific amino acids. Arrows indicate the salt bridges in the 3D structures. B. Sequence alignment of LBD of 2DBD-NRs shows the conserved amino acids according to class I and class II NRs described in [50]. The red or red highlighted letters indicate conserved amino acids found in class I NRs, the blue or blue highlighted letters indicate conserved amino acids found in class II NRs. Blocks indicate the variable inserts are deleted according to [50].

The LBD of NRs are required for NR homodimerization and/or heterodimerization ([43, 49]). In 2004, Brelivet et. al. proposed two functional classes of NRs (class I and class II) based on the conservation of amino acids in the region of LBD [50], they showed that LBD of NRs in class I could form homodimers, while LBD of members in Class II could form heterodimers with RXR. The main features of class I NRs was that their LBD possess conserved amino acids E5, E50, KR55 and RK93, these conserved amino acids formed two salt bridges, the first salt bridge was formed by amino acids E5 and KR55, and the second bridge was formed by E50 and KR93. While class II NRs possess conserved amino acids ED42, E50, R62 and HRK90, the first salt bridge was formed by amino acid ED42 and R62, and the second bridge was formed by E50 and HKR90. They further showed that RK93 was strictly conserved in class I NRs, R62 was strictly conserved in class II NRs, and E50 was conserved in both classes of NRs (Fig 10A). Our previous study showed that LBD of Sm2DBD-NRα (Sm2DBD-NRA2) could form homodimers but not heterodimers with RXR ([14]),a similar result was reported for *Echinococcus granulosus* 2DBD-NRα1 (Eg2DBDα1, Eg2DBD-NRA2) [51]. Paper [51] was published while the present paper was under review. The fact that Sm2DBD-NRα or Eg2DBDα1 could form homodimers but not heterodimers with RXRs suggested that 2DBD-NRs belongs to NR class I. Analysis of the amino acid sequence of all available 2DBD-NRs showed that all 2DBD-NRs possessed a conserved amino acid E50 and most of them possessed a strictly conserved amino acid of class I KR93. No conserved amino acids for class II NR were identified in 2DBD-NRs (Fig 10B and S5 File). This result suggested that 2DBD-NRs belong to class I NRs. Although 2DBD-NRs could form homodimers, the conserved amino acid KR55 was missing in 2DBD-NRs. KR55 was known to form the first salt bridge with E5. The absence of KR55 in 2DBD-NRs suggests that the first class I salt bridge is absent from 2DBD-NRs. The first salt bridge of class I NRs was missing in *Branchiostoma lanceolatum* NR7 (of note, NR7 was recently used to describe another group of NRs other than 2DBD-NRs, see next section) [52]. Experiments showed that 2DBD-NRs could form homodimers in a DNAindependent way [14, 51], which suggests that other factors may be involved in regulation of homodimerization of 2DBD-NRs. The formation of homodimers of 2DBD-NRs may result in four DBDs binding to a specific DNA element, the short region between two DBDs (17–22 amino acids in this region) may constraint the flexibility of their DBDs and then limit them to bind DNA in a certain region of the target gene. It is not clear whether 2DBD-NRs possessed the first salt bridge and lost it, or other class I NRs gained this salt bridge after their common ancestor split from the ancient 2DBD-NR gene.

### 4. A proposed nomenclature for 2DBD-NRs

A nomenclature for the NR superfamily was proposed, typical NRs (with both DBD and LBD) include 6 subfamilies (I-VI), and the NRs that are missing either DBD or LBD are placed in subfamily 0, irrespective of their evolutionary origin [53]. Yet the 2DBD-NRs are not included in this classification system. According to our phylogenetic analysis of 2DBD-NRs, we propose a nomenclature for 2DBD-NRs following the rule of Nomenclature System for Nuclear Receptors [53]:

#### 1) NR subfamilies are designated by Arabic numerals (NR7, 2DBD-NR)

Previously, we suggested placing 2DBD-NRs in a new NR subfamily (NR subfamily 7, NR7) [12, 14] and this was accepted by some other studies [16, 54, 55]. However, NR7 was recently used to describe NRs [52] previously known as NR8 members [55] and amphioxus NR1Hs [33, 56]. To avoid confusion of 2DBD-NRs with other NRs named as NR7, we propose to designate2DBD-NRs as a subfamily instead of using NR7.

#### 2) Groups are designated by capital letters (2DBD-NRA, 2DBD-NRB and 2DBD-NRC)

Phylogenetic analysis shows that 2DBD-NRs contain three groups: 2DBD-NRA, 2DBD-NRB and 2DBD-NRC (Fig 2). Both 2DBD-NRA and 2DBD-NRB groups contain members from protostome Spiralia and deuterostomes, while 2DBD-NRC only contains members from Nematoda.

#### 3) Individual genes are designated by Arabic numerals

For example, three 2DBD-NRs are identified in Mollusca *Aplysia californica*. One of them clustered in 2DBD-NRA group and is defined as *A*. *californica* 2DBD-NRA gene (Ac2DBD-NRA); two of *A*. *californica* 2DBD-NRs are clustered in 2DBD-NRB group, one is in Mollusca 2DBD-NRB2 subgroup 2, and it is defined as *A*. *californica* 2DBD-NRB2 (Ac2DBD-NRB2). The other one is clustered in Mollusca mono-phylogenetic 3 subgroup and it is defined as *A*. *californica* 2DBD-NRB3 (Ac2DBD-NRB3) (Fig 2).

#### 4) A lowercase letter is added at the end of the gene to designate variants

In Rotifera, 2DBD-NR genes underwent rounds of gene duplications and gave birth to different gene variations, thus, a lowercase letter is added at the end of the gene to designate variants. For example, two variations of *Brachionus calyciflorus* 2DBD-NRs are clustered in Rotifera 2DBD-NRA1 subgroup, thus, they are defined as *B*. *calyciflorus* 2DBD-NRA1a (Bc2DBD-NRA1a) and *B*. *calyciflorus* 2DBD-NRA1b (2DBD-NRA1b), respectively. All identified 2DBD-NRs, their name in this nomenclature and their GenBank Accession number are listed in Table 6.

**Table 6:** A proposed nomenclature for 2DBD-NRs.

Group	Animal	Class	Species	NR/Gene	Trivial Names	Accession Number
7A	Rotifera	*Eurotatoria*	*Rotaria magnacalcarata (Rm)*	Rm2DBD-NRA1		CAF5052617
				Rm2DBD-NRA2a		CAF1635244
				Rm2DBD-NRA2b		CAF2064338
				Rm2DBD-NRA3a		CAF1325921
				Rm2DBD-NRA3b		CAF2083731
			*Adineta steineri (As)*	As2DBD-NRA1a		CAF1578566
				As2DBD-NRA1b		CAF1211849
				As2DBD-NRA2		CAF0905228
				As2DBD-NRA3a		CAF3771316
				As2DBD-NRA3b		CAF1331931
			*A*. *ricciae (Ar)*	Ar2DBD-NRA1a		CAF1025086
				Ar2DBD-NRA1b		CAF1622127
				Ar2DBD-NRA2a		CAF0828206
				Ar2DBD-NRA2b		CAF0844309
				Ar2DBD-NRA3a		CAF1274457
				Ar2DBD-NRA3b		CAF1396991
			*Rotaria sp*. *Silwood1 (Rs1s)*	Rs1s2DBD-NRA1a		CAF3409410
				Rs1s2DBD-NRA2d		CAF0869039
				Rs1s2DBD-NRA2a		CAF0869039
				Rs1s2DBD-NRA2b		CAF3389986
				Rs1s2DBD-NRA3a		CAF3637304
				Rs1s2DBD-NRA3b		CAF4677071
			*Rotaria sp*. *Silwood2*	Rs2s2DBD-NRA1		CAF2401455
				Rs2s2DBD-NRA2a		CAF275803
			*(Rs2s)*	Rs2s2DBD-NRA3a		CAF2547262
				Rs2s2DBD-NRA3b		CAF4430748
				Rs2s2DBD-NRA3c		CAF2976618
				Rs2s2DBD-NRA2b		CAF2373862
			*R*. *sordida (Rso)*	Rso 2DBD-NRA1		CAF0748647
				Rso2DBD-NRA2a		CAF0805499
				Rso2DBD-NRA2b		CAF1284752
				Rso2DBD-NRA3a		CAF1379682
				Rso2DBD-NRA3b		CAF1365057
			*R*. *socialis (Rs)*	Rs 2DBD-NRA1		CAF4632909
				Rs2DBD-NRA2a		CAF3303110
				Rs2DBD-NRA2b		CAF4354886
				Rs2DBD-NRA3a		CAF3522866
				Rs2DBD-NRA3b		CAF3629551
			*Didymodactylos carnosus (Dc)*	Dc2DBD-NRA2a		CAF0923356
				Dc2DBD-NRA2b		CAF0904316
				Dc2DBD-NRA3a		CAF1101057
				Dc2DBD-NRA3b		CAF1014202
			*Brachionus calyciflorus*	Bc2DBD-NRA1a		CAF0776353
				Bc2DBD-NRA1b		CAF0776371
				Bc2DBD-NRA4a		CAF0820254
				Bc2DBD-NRA4b		CAF1066507
				Bc2DBD-NRA5		CAF0986382
			*B*. *plicatilis*	Bp2DBD-NRA1		RNA38473
				Bp2DBD-NRA4		RNA21328
				Bp2DBD-NRA5		RNA34403
7A	Mollusca	Bivalvia	*Pecten maximus*	Pm2DBD-NRA		XP_033734716
			*Mizuhopecten yessoensis*	My2DBD-NRA		XP_021367015
			*Crassostrea gigas*	Cg2DBD-NRA	Cg2DBDγ	XP_019919868
			*Crassostrea virginica*	Cv2DBD-NRA		XP_022331995
			*Mercenaria mercenaria*	Mm2DBD-NRA		XP_045168098
			*Dreissena polymorpha*	Dp2DBD-NRA		KAH3855004
			*Mytilus coruscus*	Mco2DBD-NRA		CAC5379089
			*Mytilus edulis*	Me2DBD-NRA		CAG2205142
			*Mytilus galloprovincialis*	Mg2DBD-NRA		VDI76762
		Gastropoda	*Elysia chlorotica*	Ech2DBD-NRA		RUS87101
			*Elysia marginata*	Ema2DBD-NRA		GFR71482
			*Plakobranchus ocellatus*	Po2DBD-NRA		GFN82368
			*Aplysia californica*	Aca2DBD-NRA		XP_005103360
			*Biomphalaria glabrata*	Bg2DBD-NRA		XP_013065455
			*Bulinus truncatus*	BT2DBD-NRA		KAH9507289
			*Candidula unifasciata*	Cu2DBD-NRA		CAG5125116
			*Pomacea canaliculata*	Pca2DBD-NRA		XP_025103483
			*Haliotis rubra*	Hrub2DBD-NRA		XP_046571180
			*Haliotis rufescens*	Hruf2DBD-NRA		XP_046364309
			*Gigantopelta aegis*	Ga2DBD-NRA		XP_041351218
			*Lottia gigantea*	Lg2DBD-NRA	Lg2DBD-NRγ	XP_009064814
	Annelida	Clitellata	*Helobdella robusta*	Hr2DBD-NRA3a		XP_009022576
				Hr2DBD-NRA3b		XM_009022049
				Hr2DBD-NRA3c		XP_009022817
				Hr2DBD-NRA2		XM_009018880
		Polychaeta	*Capitella teleta*	Ct2DBD-NRA2		ELU14753
				Ct2DBD-NRA1		ELU03757
			*Lamellibrachia satsuma*	Ls2DBD-NRA		KAI0243018
			*Dimorphilus gyrociliatus*	Dgy2DBD-NRA1		CAD5121447
			*Owenia fusiformis*	Ofu2DBD-NRA1		CAC9635804
7A	Platyhelminthes	Rhabditophora	*Macrostomum lignano*	Ml2DBD-NRA1		PAA89302
				Ml2DBD-NRA2		PAA53885
			*Schmidtea mediterranea*	Sme2DBD-NRA1	nhr-1	
				Sme2DBD-NRA2	nhr-2	
				Sme2DBD-NRA3a	nhr-3	
				Sme2DBD-NRA3b	nhr-6	
		Monogenea	*Gyrodactylus salaris*	Gs2DBD-NRA1		
				Gs2DBD-NRA2		
				Gs2DBD-NRA3		
			*Protopolystoma xenopodis*	Px2DBD-NRA1		
				Px2DBD-NRA2		VEL20704, partial DBD
				Px2DBD-NRA3		VEL22304, partial DBD
		Cestoda	*Dibothriocephalus latus*	Dl2DBD-NRA1		VDK35285, 2^nd^ DBD)
				Dl2DBD-NRA2		
				Dl2DBD-NRA3		
			*Echinococcus Canadensis*	Ec2DBD-NRA1		
				Ec2DBD-NRA2		
				Ec2DBD-NRA3		
			*Echinococcus granulosus*	Eg2DBD-NRA1	Eg2DBDg	KAH9284012
				Eg2DBD-NRA2	Eg2DBDα	AZM65758
				Eg2DBD-NRA3	Eg2DBDb	KAH9280969.1
			*Echinococcus multilocularis*	Em2DBD-NRA1		CDI98537
				Em2DBD-NRA2		CDS36659
				Em2DBD-NRA3		CDS37339
			*Echinostoma caproni*	Eca2DBD-NRA1		VDP70475.1, Partial DBD
				Eca2DBD-NRA2		VDP81705
				Eca2DBD-NRA3		
			*Hydatigera taeniaeformis*	Ht2DBD-NRA1		VDM16170, Partial
				Ht2DBD-NRA2		VDM26556, Partial
				Ht2DBD-NRA3		VDM33972
			*Hymenolepis diminuta*	Hd2DBD-NRA1		VDL20127
				Hd2DBD-NRA2		VDL59771
				Hd2DBD-NRA3		VUZ44963
			*Hymenolepis microstoma*	Hm2DBD-NRA1		CDS27504
				Hm2DBD-NRA2		CUU99503
				Hm2DBD-NRA3		CDS31978
			*Hymenolepis nana*	Hn2DBD-NRA1		VDO05171
			(Rodentolepis nana)	Hn2DBD-NRA2		VDO08914
				Hn2DBD-NRA3		VDN97648
			*Mesocestoides corti*	Mc2DBD-NRA1		VDD74272
				Mc2DBD-NRA2		VDD74096
				Mc2DBD-NRA3		VDD77213
			*Schistocephalus solidus*	Ss2DBD-NRA1		VDL94711, 2nd DBD
				Ss2DBD-NRA2		VDL97469, 2nd DBD
				Ss2DBD-NRA3		VDL91072, partial DBD
			*Sparganum proliferum*	Spa2DBD-NRA1		VZI04907
				Spa2DBD-NRA2		VZI51357, VZI24038, VZI24041
				Spa2DBD-NRA3		VZI32835
			*Spirometra erinaceieuropae* ***i***	Se2DBD-NRA1		VDM16170
				Se2DBD-NRA2		VZI28417
				Se2DBD-NRA3		VZI27737
7A	Platyhelminthes	Cestoda	*Taenia asiatica*	Ta2DBD-NRA1		VDK37619
				Ta2DBD-NRA2		VUZ40124
				Ta2DBD-NRA3		VDK20940
			*Taenia multiceps*	Tm2DBD-NRA1		
				Tm2DBD-NRA2		
				Tm2DBD-NRA3		
			*Taenia saginata*	Ts2DBD-NRA1		
				Ts2DBD-NRA2		
				Ts2DBD-NRA3		
			*Taenia solium*	Tso2DBD-NRA1		
				Tso2DBD-NRA2		
				Tso2DBD-NRA3		
		Trematoda	*Clonorchis sinensis*	Cs2DBD-NRA1		KAG5448022
				Cs2DBD-NRA2		GAA27896
				Cs2DBD-NRA3		GAA48469
			*Fasciolopsis buski*	Fb2DBD-NRA1		KAA0185392
				Fb2DBD-NRA2		KAA0191852, partial
				Fb2DBD-NRA3		KAA0198729, partial
			*Fasciola gigantica*	Fg2DBD-NRA1		TPP59537, partial
				Fg2DBD-NRA2		TPP61208
				Fg2DBD-NRA3		TPP62972, TPP57451
			*Fasciola hepatica*	Fh2DBD-NRA1		THD25668
				Fh2DBD-NRA2		THD25920
				Fh2DBD-NRA3		HD26972
			*Opisthorchis felineus*	Of2DBD-NRA1		TGZ59947
				Of2DBD-NRA2		TGZ60667
				Of2DBD-NRA3		TGZ71895
			*Opisthorchis viverrini*	Ov2DBD-NRA1		OON22325, 2nd DBD
				Ov2DBD-NRA2		XP_009167226
				Ov2DBD-NRA3		XP_009175932
			*Paragonimus heterotremus*	Phe2DBD-NRA1		KAF5404614
				Phe2DBD-NRA2		KAF5404035
				Phe2DBD-NRA3		KAF5405364
			*Paragonimus skrjabini miyazakii*	Psm2DBD-NRA1		KAF7262107, KAF7262108
				Psm 2DBD-NRA2		KAF7261790
				Psm 2DBD-NRA3		KAF7232516
			*Paragonimus westermani*	Pw2DBD-NRA1		KAF8569986
				Pw2DBD-NRA2		KAA3679217
				Pw2DBD-NRA3		KAF8566917
			*Schistosoma bovis*	Sb2DBD-NRA1		RTG85541, partial DBD
				Sb2DBD-NRA2		RTG87273
				Sb2DBD-NRA3		RTG84925, partial DBD
			*Schistosoma curassoni*	Sc2DBD-NRA1		VDO64665
				Sc2DBD-NRA2		VDP76164, VDP65226
				Sc2DBD-NRA3		VDP48464
			*Schistosoma haematobium*	Sh2DBD-NRA1		KAH9594422
				Sh2DBD-NRA2		XP_035585440, XP_012799787
				Sh2DBD-NRA3		KAH9592582
			*Schistosoma japonicum*	Sj2DBD-NRA1		TNN12920
				Sj2DBD-NRA2		KAH8855859
				Sj2DBD-NRA3		TNN09743
			*Schistosoma margrebowiei*	Sma2DBD-NRA1		VDO62366
				Sma2DBD-NRA2		VDO64249, VDP52470
				Sma2DBD-NRA3		VDP26109
			*Schistosoma rodhaini*	Sr2DBD-NRA1		
				Sr2DBD-NRA2		
				Sr2DBD-NRA3		
			*Schistosoma mattheei*	Smt2DBD-NRA1		VDP64780, partial DBD
				Smt2DBD-NRA2		VDP67915, VDP82804
				Smt2DBD-NRA3		VDP61516, VDP04037
			*Schistosoma mansoni*	Sm2DBD-NRA1	Sm2DBDγ	AY698061
				Sm2DBD-NRA2	Sm2DBDα	AH013462
				Sm2DBD-NRA3	Sm2DBDβ	AY688251
			*Trichobilharzia regenti*	Tr2DBD-NRA1		
				Tr2DBD-NRA2		VDQ08843, partial
				Tr2DBD-NRA3		VDP99859, VDQ10779
	Brachiopoda	Lingulata	*Lingula anatina*	La2DBD-NRA1		XP_013400324
				La2DBD-NRA2		XP_013400322
	Nemertea	Pilidiophora	*Notospermus geniculatus*	Ng2DBD-NRA		
	Chordata	Leptocardii	*Branchiostoma belcheri*	Bb2DBD-NRA		XM_019778353
			*Branchiostoma floridae*	Bf2DBD-NRA		XM_035818392
			*Branchiostoma lanceolatum*	Bl2DBD-NRA		CAH1264236
7B	Mollusca	Bivalvia	*Crassostrea gigas*	Cg2DBD-NRB1	Cg2DBD-NRδ	XP_011428801
			*Crassostrea virginica*	Cv2DBD-NRB1		XP_022329720
			*Pecten maximus*	Pm2DBD-NRB1		XP_033734800
			*Mytilus coruscus*	Mco2DBD-NRB1		CAC5405050
			*Mytilus edulis*	Me2DBD-NRB1		CAG2230085
			*Mytilus galloprovincialis*	Mg2DBD-NRB1		VDI78403
			*Mercenaria mercenaria*	Mm2DBD-NRB1		XP_045175060
			*Mizuhopecten yessoensis*	My2DBD-NRB1		XP_021378725
		Gastropoda	*Aplysia californica*	Aca2DBD-NRB3		XP_005112947
			*Biomphalaria glabrata*	Bg2DBD-NRB3		XP_013069617
			*Bulinus truncatus*	Btd2DBD-NRB3		KAH9507198
			*Haliotis rufescens*	Hruf2DBD-NRB1		XP_046369823
			*Lottia gigantea*	Lg2DBD-NRB1	Lg2DBD-NRα/β	XP_009049651
			*Gigantopelta aegis*	Ga2DBD-NRB1		XP_041351328
			*Aplysia californica*	Aca2DBD-NRB2		XP_005095909
			*Haliotis rubra*	Hrub2DBD-NRB2		XP_046577572
			*Haliotis rufescens*	Hruf2DBD-NRB2		XP_046379417
			*Pomacea canaliculata*	Pca2DBD-NRB2		PVD26031
			*Plakobranchus ocellatus*	Po2DBD-NRB2		GFO27501
			*Elysia marginata*	Ema2DBD-NRB3		GFS22057
			*Elysia chlorotica*	Ech2DBD-NRB3		RUS84791
			*Plakobranchus ocellatus*	Pod2DBD-NRB3		GFO07362
			*Pomacea canaliculata*	Pca2DBD-NRB3		PVD26170
	Annelida	*Clitellata*	*Helobdella robusta*	Hr2DBD-NRB2		XP_009031176
		Polychaeta	*Lamellibrachia satsuma*	Ls2DBD-NRB1		KAI0214066
				Ls2DBD-NRB2a		KAI0207292
				Ls2DBD-NRB2b		KAI0207833
			*Capitella teleta*	Ct2DBD-NRB1		ELT90493, 2nd DBD
				Ct2DBD-NRB2		
			*Owenia fusiformis*	Ofu2DBD-NRB2		CAH1791723
	Brachiopoda	Lingulata	*Lingula anatina*	La2DBD-NRB		XM_024075933
	Phoronida		*Phoronis australis*	Pa2DBD-NRB		
	Echinodermata	Asteroidea	*Acanthaster planci*	Ap2DBD-NRB		XM_022235619
			*Asterias rubens*	Aru2DBD-NRB		XM_033772834
			*Patiria miniata*	Pmi2DBD-NRB		XM_038217445
		Echinoidea	*Lytechinus variegatus*	Lv2DBD-NRB		XM_041613378
			*Strongylocentrotus purpuratus*	Spur2DBD-NRB		XM_030998347
		Crinoidea	*Anneissia japonica*	Aja2DBD-NRB		XM_033246620
7C	Nematoda	Chromadorea	*Aphelenchus avenae*	Aa2DBD-NRC1		KAH7714729
				Aa2DBD-NRC2		KAH7714119
				Aa2DBD-NRC3		KAH7695147
				Aa2DBD-NRC4		KAH7714341
				Aa2DBD-NRC5		KAH7721403
				Aa2DBD-NRC6		KAH7705661
				Aa2DBD-NRC7		KAH7711540
			*Caenorhabditis brenneri*	Cb2DBD-NRC		EGT43828
			*Caenorhabditis remanei*	Cr2DBD-NRC1		KAF1767687
				Cr2DBD-NRC2		XM_003107303
				Cr2DBD-NRC3		KAF1767743
				Cr2DBD-NRC4		KAF1767675

## Conclusion

In this study, 2DBD-NRs were identified in both protostomes and deuterostomes. Phylogenetic analysis shows that 2DBD-NRs consist of three groups, two groups are present in both protostomes and deuterostomes and the members of the third group are only found in Nematoda. Members of 2DBD-NRA and 2DBD-NRB are identified in both protostomes and deuterostomes, this result suggests that at least two 2DBD-NR genes were present in a common ancestor of the protostomes and deuterostomes. Phylogenetic analysis shows that 2DBD-NRs underwent gene duplication after the split of the different animal phyla. Thus, most of 2DBD-NR genes in a certain animal phylum are paralogues, rather than orthologues like in other animal phyla. 2DBD-NR gene losses occurred in different animal phyla, for example, 2DBD-NRA was missing in Phoronida and Echinodermata, and 2DBD-NRB was not identified in Nemertea, Rotifera, Platyhelminthes and Chordata. Sequence analysis shows that 2DBD-NRs possess highly conserved regions similar to that of typical NRs. The different P-P module **(**amino acid sequence of P-box in the first DBD and the second DBD) in different 2DBD-NR groups may affect their DBD binding abilities. Since very few studies have been carried out about 2DBD-NRs, little is known about their function. This study demonstrates that 2DBD-NR genes are widely distributed in both protostomes and deuterostomes, their role in regulation of the animal development awaits be revealed.

## Supporting information

S1 File P-box sequence in 2DBD-NRs. (PDF)

S2 File Sequence alignment of both DBDs of 2DBD-NRs. (PDF)

S3 File Ti sequence for sequence logo. (PDF)

S4 File AF2 sequence (yellow highlighted) for sequence logo. (NCBI accession number of 2DBD-NRs see Table 6, accession number of other NR is in the bracket after each NR name).
(PDF)

S5 File LBD sequence alignment to identify the conserved amino acids in class I or class II NRs. LBD sequence alignment of all 2DBD-NRs to identify the conserved amino acids in class I or class II NRs according to [50] (Signature of the oligomeric behavior of nuclear receptors at the sequence and structural level. Blocks indicate the variable inserts are deleted according to [50]).
(PDF)

## Abbreviations

2DBD-NR: nuclear receptor with two DBDs
DBD: DNA binding domain
LBD: ligand binding domain
NTSS: N-terminal signature sequence
P-P module: the P-box sequence in the first DBD and the second DBD of 2DBD-NRs
