# Supplementary material for: Updated knowledge and a proposed nomenclature for nuclear receptors with two DNA binding domains (2DBD-NRs)
Source: PLoS One. 2023 Sep 12;18(9):e0286107. doi: 10.1371/journal.pone.0286107 (PMC10497141; doi:10.1371/journal.pone.0286107)
Supplement: S1 File — (PDF) [file pone.0286107.s001.pdf]

Supplemental material 1. P-box sequence in 2DBD-NRs

**2DBD-NR**	**P-box sequence**	
	**The first DBD**	**The second DBD**
**2DBD-NRA**	**EACKK**	**EGCKG**
Rotifera 2DBDs		
Rotifera 2DBD-NRA3 group	EACKK	EACKG
Ar2DBD-NRA2b	EACKK	ESCKG
Hr2DBD-NRA3b	EACKK	ERCKG
Dc2DBD-NRA3b	EACKK	EACKC
**2DBD-NRB**	**LPCKS**	**EGCKK**
Ct2DBD-NRB2	IPCKA	EGCKK
Hr2DBD-NRB2, Ls2DBD-NRB2a	VPCKT	EGCKK
Ls2DBD-NRB2b	LPCKT	EGCKK
La2DBD-NRB	LACKS	EGCKK
Of2DBD	WTCKT	LGCTK
**Echinodermata 2DBD-NRB**	**EACKS**	**EGCKG**
**2DBD-NRC**		
Cb2DBD-NRC	DSCKC	DSCKW
Cr2DBD-NRC1	DNCRA	GSCRM
Cr2DBD-NRC2	DGCRT	EGCRV
Cr2DBD-NRC3	EGCRS	EGCKG
Cr2DBD-NRC4	EGCKK	EACKT
Aa2DBD-NRC1	TGCKT	LGCKA
Aa2DBD-NRC2	AGCRI	FGCKT
Aa2DBD-NRC3, Aa2DBD-NRC4	NGCKT	HGCKA
Aa2DBD-NRC5	NGCKT	HGCKS
Aa2DBD-NRC6	TACWM	LGCKG
Aa2DBD-NRC7	TACWM	HACKT
