# Supplementary material for: Updated knowledge and a proposed nomenclature for nuclear receptors with two DNA binding domains (2DBD-NRs)
Source: PLoS One. 2023 Sep 12;18(9):e0286107. doi: 10.1371/journal.pone.0286107 (PMC10497141; doi:10.1371/journal.pone.0286107)
Supplement: S2 File — (PDF) [file pone.0286107.s002.pdf]

Supplemental material 2. Sequence alignment of both DBDs of 2DBD-NRs

	The 1st DBD					The 2nd DBD				
		P-box		D-Box			P-box		D-Box	
	CxxCxxx xxxxxxxxxxC	xxC		CxxxxxC	xxxxxxxxxCxxC	CxxCxxxxxxxxxxxxxC	xxC		CxxxxxC	xxxxxxxxxCxxC
**Ct2DBD-NRA1**	CQVCGDV-AAGFHCGAYVC	EACKK	FFVRCLKQDA------FV	CPRNNQC	DITKDTRTQCQRCRYRKCLALGM	CSVCGAPSSGFHFGAITC	EGCKG	FFRRT-IK--ERDI-LKYQ--	CSREGTC	IITTATRNICKFCRYQKCLDAGM
**Ofu2DBD-NRA1**	CQVCNDM-AAGFHCGAYVC	EACKK	FFLRCTKQD-------YM	CGKQGSC	IITRETRTQCQSCRYEKCLFLGM	CRVCGARSSGYHFGAITC	EGCKG	FFRRT-IK--ERTA-DQYL--	CSRVGNC	EITLESRNSCKYCRYKKCLDAGM
**Dgy2DBD-NRA1**	CQVCNDV-AAGFHCGAYVC	EACKK	FFVRCQKQQ-------FA	CQRNRDC	VVTKETRTQCQSCRYQKCIQLGM	CKVCGAPSSGYHFGAITC	EGCKG	FFRRT-VR--ERDS-CKYV--	CARNGRC	TITTATRNMCKFCRYQKCRGVGM
**Ls2DBD-NRA1**	CQVCNDI-AAGFHCGAYVC	EACKK	FFVRCLKQDT------FM	CPRKKDC	DITKETRTQCQYCRYQKCLAIGM	CKVCGAPSSGYHFGAITC	EGCKG	FFRRT-VK--ERDS-LKYQ--	CSHFGNC	QITTQTRNVCKFCRYQKCLEVGM
**Hr2DBD-NRA2**	CQVCGDV-AAGFHCGAYVC	EACKK	FFLRCLKTGRGL---MFL	CPKNSNC	DITKETRSHCQRCRYQRCIALSM	CKVCKAPSSGFHFGAITC	EGCKG	FFRRM-LK--EQVH-RKFI--	CSNDERC	NIDSANRTTCKSCRFNKCIKIGM
**Ct2DBD-NRA2**	CQVCGDI-AAGFHCGAYVC	EACKK	FYLRCLKSQANT---NFV	CPKNSTC	VVTKETRSHCQYCRFNKCIQLGM	CKVCTAPSSGFHFGAITC	EGCKG	FFRRM-LK--EQLH-RKFQ--	CSGNDQC	DITSATRTTCKACRFSKCISVGM
Hr2DBD-NRA3c	CDVCADV-SSGFYCGAFVC	EACKK	FFVRCLQMKEKFR---AV	CTKDGKC	VINKTTRTQCSKCRYEKCISLNM	CKVCGAPSSGFHFGVTTC	EGCKG	FYRRC-VK--KYDT-AKFH--	CTNDNNC	DVTWKTRNVCKFCRYQKCQDVGM
Hr2DBD-NRA3b	CQICGDS-AAGFYCGAYVC	EACKK	FFIRSLRSKISRDF--NP	CPSDGQC	HVTKETRTQCPQCRYNKCLSFNM	CKVCDFPSSGFHYGAITC	ERCKG	FFRRC-IL--DRNI-ELLG--	CM????C	RLLKKKRNMCKRCRYLKCLAVGM
**Hr2DBD-NRA3a**	CQVCTDP-AAAFYCGAQVC	EACKK	FFIRSWKNSTENN---YV	CLQDRKC	VLTKESRKHCAYCRYDRCLQLKM	CRICGAPSSGFHFGVITC	EGCKG	FFRRR-CH--DNRF-DKFK--	CNENNCC	VISAANRSMCRACRLRKCLDSGM
**Em2DBD-NRA1**	CDICGDV-SAGFHCNAYVC	EACKK	FFIRSSKNENY-----YS	CSKVNAC	EVNKDTRTHCQRCRFYKCLRIGM	CRVCGAQSSGFHFGAITC	EGCKG	FFRRT-IS--ERDN-QRYT--	CRNGGTC	LITLATRNNCKSCRYRKCLSVGM
**Sm2DBD-NRA1**	CDICGDV-AAGFHCNAYVC	EACKK	FFIRSSKGENF-----YT	CTKSNTC	EINKDTRTHCQRCRYQKCIRLGM	CRVCGAKSSGFHFGAITC	EGCKG	FFRRT-IN--ERES-QRYT--	CRNGGNC	AVTGATRNNCKSCRYRRCLAVGM
**Px2DBD-NRA1**	CDICGDV-SAGYHCNAYVC	EACKK	FFIRSSKGRSY-----YT	CTKASNC	DIKKDTRTHCQYCRFQKCLHIGM	CRVCGAKSSGFHFGAITC	EGCKG	FFRRT-VN--ERDG-HRYT--	CRNGGNC	SVTGSTRNNCKSCRYQRCLLVGM
**Sme2DBD-NRA1**	CDVCGDV-SAGFHCSAFVC	EACKK	FFIRSSKGDSY-----YS	CTKNNNC	EIVKDTRTHCQYCRFQKCLHLGM	CRVCGAQSSGFHFGAITC	EGCKG	FFRRT-IN--EREN-QKYT--	CRNGGNC	VINLATRNNCKSCRYKKCLAMGM
**Ml2DBD-NRA1a**	CDVCGDT-AAGFHCGAFVC	EACKK	FFIRSTRAGMQ-----YS	CSKVGRC	DVTKETRTHCQHCRYKKCLMLNM	CRVCGASSSGFHFGAITC	EGCKG	FFRRT-IK--ERDA-GKYI--	CSKGGGC	EINQQSRNACKSCRFAKCIRAGM
**Ml2DBD-NRA1b**	CDVCGDT-AAGFHCGAFVC	EACKK	FFIRSSRAEQQ-----YA	CSKSGSC	DITKETRTHCQFCRYKKCLALRM	CRVCGASSSGFHFGAITC	EGCKG	FFRRT-IK--ERDS-GKYV--	CSKGGGC	EINKSSRNACKSCRFTKCIRAGM
**Sm2DBD-NRA2**	CQVCGEL-AAGFHHGAYVC	EACKK	FFMRHSMADTKP---TNV	CPTGGNC	IVAKGSRGKCQICRYRKCLLVGM	CRVCGGRSSGFHFGALTC	EGCKG	FFRRT-EG--SS---NSLV--	CVGQNAC	TITPRSRNACKSCRFRRCLAAGM
**Px2DBD-NRA2**	CQVCGEL-AAGFHHGAYVC	EACKK	FFMRHSLNDSKP---TNS	CPSAGNC	VVAKGSRGKCQQCRYRKCLDVGM	CRVCGGRSSGFHFGALTC	EGCKG	FFRRT-EG--TS---QRLA--	CVGQDNC	AITPRSRNACKGCRFRRCLTAGM
**Em2DBD-NRA2**	CQICGEL-AAGFHHGAYVC	EACKK	FFMRHSLANGRS---TTT	CPSGGNC	VIAKSSRGRCQQCRYKKCLEVGM	CRVCGGRSSGFHFGALTC	EGCKG	FFRRT-EE--SA---SRLV--	CVGKDNC	TITPRSRNVCKACRFRRCLRAGM
**Sme2DBD-NRA2**	CQVCRES-AAGFHHGAYVC	EACKK	FFMRHNLNSTKF---TIP	CPTGGQC	AQLKTGRIKCQSCRFKKCVSIGM	CRVCGGKSSGFHFGALTC	EGCKG	FFRRT-ES--TK---QHLA--	CIGNNNC	CLTGSSRNLCKSCRFNRCLGVGM
**Px2DBD-NRA3**	CQVCGQP-AVGFHHRAYVC	EACKK	FFTRHASARLRSA-SSMA	CPMGGGC	RVEGPGRGKCPHCRFKKCLDLGM	CRVCSGPSSGFHFGALTC	EGCKG	FFRRT-VL--SG---VHLE--	CLNNNDC	PVTPSNRNMCKACRFNRCLAVGM
**Sm2DBD-NRA3**	CQICGQP-AVGFHHRAYVC	EACKK	FFMRHTAARLRNS----I	CPMGGRC	RVEGPGRGKCPHCRYRKCLELGM	CRVCSGPSSGFHFGALTC	EGCKG	FFRRT-VL--SN---VRLE--	CPGNNDC	PITPANRNMCKSCRFQRCLAVGM
**Em2DBD-NRA3**	CQVCGQP-SVGFHHRAYVC	EACKK	FFTRHLTNRIKKEKSSVV	CPMGGNC	KIEGPGRGKCPHCRFRKCLDLGM	CRVCGGPSSGFHFGALTC	EGCKG	FFRRT-VH--SN---SIPQ--	CLGNQTC	RITPSNRNMCKSCRFKKCLEVGM
**Sme2DBD-NRA3**	CQVCNCL-AAGFHHGAYVC	EACKK	FFKRYSTNQLNSK---VV	CKK--NC	DLEKIGRRKCSFCRYQKCLNVGM	CRVCGGKSSGFHFGSLTC	EGCKG	FFRRA-EP--LK---DKLE--	CVGKLNC	VIDSSHKMPCKWCRYHRCIKVGM
**Sme2DBD-NRA4**	CQICTKN-SVGFHYGAYVC	EACKK	FFVRHASGVCRLY---GS	CQEKGKC	DLNIDGRGKCQHCRYQRCNEIGM	CQVCGAKSSGLHFGCITC	EGCKG	FFRRM-IK--FK---GSLV--	CFNDNKC	KLDLKNRSTCKSCRMNRCLSVGM
**Ng2DBD-NRA**	CKVCGDM-SSGFHCGAYVC	EACKK	FFIRSMKHSS------YV	CSKNKTC	RITKESRTQCQYCRYQKCLSSGM	CKVCGAPSSGFHFGAITC	EGCKG	FFRRT-IK--ERDS-YRYA--	CSKGGNC	SITLSTRNACKYCRFKQCIDVGM
**La2DBD-NRA1**	CQVCGDL-SSGFYCGAFVC	EACKK	FYLRSLK-QNSK----YS	CLKKGQC	QVSKETRTVCQYCRFRKCIAIGM	CRVCGAPSSGFHFGAVTC	EGCKG	FFRRT-VR--ERD--NYYS--	CSKRQQC	SITETTRNICKYCRYKKCLSVGM
**La2DBD-NRA2**	CSVCGET-SSGFHCGAITC	EACKK	FFIRSCQGESNK----YV	CTKNNNC	EIMRATRTQCQRCRYEKCLAVGM	CSVCKAPASGFHFGAVTC	EGCKG	FFRRS-VK--EKNGLDYY---	CEQGGNC	PITETTRNTCRYCRYKKCLDVGM
**Bl2DBD-NRA_**	CQVCGDV-SVGFHCGACVC	EACKK	FFIRSTKDGGDR--TKFK	CSKQQKC	DITKQTRSQCQYCRLQKCLAVGM	CKVCGAESSGFHFGVDTC	EGCKG	FFRRS-QL--KTEY-ESYR--	CSKSKDC	AINVVTRNLCRYCRYAKCLTVGM
**Bf2DBD-NRA**	CQVCGDV-SVGFHCGACVC	EACKK	FFIRSTKDGGDR--TKFK	CSKQQKC	DITKQTRSQCQYCRLQKCLAVGM	CKVCGAESSGFHFGVDTC	EGCKG	FFRRS-QL--KTEY-ESYR--	CSKSKDC	AINVVTRNLCRYCRYAKCLAVGM
**Cv2DBD-NRA**	CQVCGDK-AAGFYCGAFVC	EACKK	FFMRASRQQKIK----YT	CLKAKKC	RITKESRVQCQYCRFQKCLSLNM	CQVCNAPSSGFHFGALTC	EGCKG	FFRRI-AK--EKGS-KPYR--	CSKSQNC	VVTTGTRNLCRACRYQKCLDIGM
**Cg2DBD-NRA**	CQVCGDK-AAGFYCGAFVC	EACKK	FFMRASRQQKVK----YT	CLKAKNC	KITKESRVQCQYCRFQKCLSLNM	CQVCNAPSSGFHFGALTC	EGCKG	FFRRI-AK--EKGN-KQYR--	CSKSQNC	EVTTGTRNLCRACRYQKCLDIGM
**Pm2DBD-NRA**	CQVCGDK-AAGFYCGAFIC	EACKK	FFMRASKLEKIK----YV	CLRVQKC	VITKESRVQCQYCRFQKCLSLKM	CRVCSAPSSGFHFGALTC	EGCKG	FFRRM-AK--EREC-QRYK--	CSKNGNC	DVNTVTRNLCKACRYRKCLESGM
**My2DBD-NRA**	CQVCGDK-AAGFYCGAFIC	EACKK	FFMRANKLEKLK----YV	CLRVGRC	VITKESRVQCQYCRYQKCVSLKM	CRVCSAPSSGFHFGALTC	EGCKG	FFRRM-AK--EREC-QRYK--	CSKNGSC	EVNTITRNLCKACRYRKCVECGM
**Dp2DBD-NRA**	CQVCGDV-AAGFYCGAYIC	EACKK	FYMRAYKQDKLK----YV	CLRDKKC	VITKESRVQCQYCRFQKCVQLNM	CRVCSAPSSGFHFGAITC	EGCKG	FFRRM-AK--EREA-EKYQ--	CNKSGDC	EINMVTRNLCKACRYRACIKAGM
**Mm2DBD-NRA**	CQVCGDM-AAGFYCGAFIC	EACKK	FYMRAYKQDKLK----YV	CLRDKKC	MITKESRVQCQYCRFQKCVTLNM	CRVCSAPSSGFHFGAITC	EGCKG	FFRRM-AK--EREP-EKYQ--	CNKKGRC	EINMVTRNLCKACRYRSCREAGM
**Mg2DBD-NRA**	CQVCGDK-AAGFYCGAFAC	EACKK	FFMRSCRNDKTK----YV	CLRDKKC	QMTAESRVQCQYCRLQKCLSLNM	CRVCGAPSSGFHFGALTC	EGCKG	FFRRM-AK--EREL-GKYR--	CSKTGSC	EINTSTRNLCKSCRYKKCVDAGM
**Me2DBD-NRA**	CQVCGDK-AAGFYCGAFAC	EACKK	FFMRSCRNDKTK----YV	CLRDKKC	QMTAESRVQCQYCRLQKCLSLNM	CRVCGAPSSGFHFGALTC	EGCKG	FFRRM-AK--EREL-GKYR--	CSKTGSC	EINTSTRNLCKSCRYKKCVDAGM
**Mc2DBD-NRA**	CQVCGDK-AAGFYCGAFAC	EACKK	FFMRSCRNDKTK----YV	CLRDKKC	QMTAESRVQCQYCRLQKCLSLNM	CRVCSAPSSGFHFGALTC	EGCKG	FFRRM-AK--EREL-GKYR--	CSKTGSC	EINTSTRNLCKSCRYKKCVDAGM
**Ech2DBD-NRA** 1	CQVCGDQ-AAGFYCGAYIC	EACKK	FFIRASKLKQMK----YV	CLKQKNC	NITKETRVHCQFCRYQKCLSLNM	CRVCSAPSSGFHFGALTC	EGCKG	FFRRM-VN--EREP-GTYS--	CGRGGNC	EVNSMTRNMCKACRYAKCLQIGM
**Ema2DBD-NRA**	CQVCGDQ-AAGFYCGAYIC	EACKK	FFIRASKLKQMK----YI	CLKQKNC	KITKESRVHCQYCRYQKCLSLDM	CRVCSAPSSGFHFGALTC	EGCKG	FFRRM-VN--EREP-GTYT--	CGKGGKC	EVNSMTRNMCKACRYAKCLMIGM
**Po2DBD-NRA**	CQVCGDH-AAGFYCGAYIC	EACKK	FFIRAAKLKQIK----YV	CLKQKNC	IITKESRVHCQYCRYQKCLSLNM	CRVCSAPSSGFHFGALTC	EGCKG	FFRRM-VN--EREP-GTYK--	CGKGGNC	KVNSMTRNMCKACRYAKCLQIGM
**Bg2DBD-NRA**	CQVCGDL-AAGFYCGAYIC	EACKK	FFIRASKLERPR----YV	CLRQKDC	VITKESRVHCQYCRYQKCLQLNM	CRVCAAPSSGFHFGALTC	EGCKG	FFRRM-VK--EREA-YNYK--	CSKSGTC	EVNAMTRNMCKACRYNKCIQIGM
**Aca2DBD-NRA**	CQVCDDL-AAGFYCGAYIC	EACKK	FFIRASKLECPR----YV	CLRQKNC	IITKESRVHCQFCRYQKCLQLNM	CRVCSAPSSGFHFGALTC	EGCKG	FFRRM-VK--EREA-CAYK--	CSKDGNC	EVNAMTRNMCKACRYHKCLQIGM
**Cu2DBD-NRA**	CQVCEDA-AAGFYCGAYIC	EACKK	FFIRASKQNELR----YQ	CPRSRSC	PITRESRVHCQYCRYQKCLRLSM	CRVCSAPSSGFHFGALTC	EGCKG	FFRRM-VK--ERDS-YSYK--	CGRNGHC	EINATTRNMCKACRYNKCLQIGM
**Pca2DBD-NRA**	CQVCGDI-AAGFYCGAFIC	EACKK	FFIRASKQEKHK----FV	CLRQGLC	KITKESRVQCQFCRYQKCLDLQM	CRVCSAPSSGFHFGALTC	EGCKG	FFRRM-VK--EREP-GSYR--	CSAGGTC	EISAQTRNMCKACRYQKCVDIGM
**Hruf2DBD-NRA**	CQVCGDV-AAGFYCGAYIC	EACKK	FFVRASRQEKVK----FV	CHKSEDC	VITKETRVRCQHCRYQKCLTLHM	CRVCGGASSGFHFGALTC	EGCKG	FFRRM-AK--EREW-HSYK--	CSKEDRC	EISMLTRNVCKACRYRKCLDAGM
**Hrub2DBD-NRA**	CQVCGDV-AAGFYCGAYIC	EACKK	FFVRASRQEKVK----FV	CHKSEDC	VITRETRVRCQHCRYQKCLSLDM	CRVCGGSSSGFHFGALTC	EGCKG	FFRRM-AK--EREW-HSYK--	CSKDDKC	EISMLTRNVCKACRYRKCLEAGM
**Ga2DBD-NRA**	CQVCGDV-AAGFYCGAFIC	EACKK	FFVRTCKQDKVK----YA	CHKNQCC	KITKENRIRCQYCRYQKCLSVNM	CRVCGGPSSGFHFGALTC	EGCKG	FFRRM-VK--ERSP-SMYQ--	CLKDNDC	NINMFTRNICKSCRYKKCTEVGM
**Lg2DBD-NRA**	CDVCGDK-AAGFYCGAFVC	EACKK	FFIRAAKQGEVK----YK	CLRDGNC	TITKVNRIQCQFCRYQKCVALNM	CQVCGAQSSGFHFGALTC	EGCKG	FFRRM-AK--ERSS-NSYI--	CSKGNMC	LVSISTRNSCKACRYQKCILVGM
**Rs1s2DBD-NRA2a**	CQVCGEN-ASGWHCGSITC	EACKK	FFLRSVNEEYR-----YK	CMKDKKC	IITRTTRTQCQYCRYMKCIEVGM	CAVCQNPSSGIHFGATTC	EGCKG	FFRRT-MK--ERTP-QRYK--	CPEKNNC	EVNTSTRSACRACRFQKCLTAGM
**Rs2s2DBD-NRA2**	CQVCGEN-ASGWHCGSITC	EACKK	FFLRSVNEEYR-----YK	CMKDKKC	IITRTTRTQCQYCRYMKCIEVGM	CAVCQHPSSGIHFGATTC	EGCKG	FFRRT-MK--ERTP-QRYK--	CPEKNNC	EVNTSTRSACRACRFQKCLTAGM
**Rso2DBD-NRA2a**	CQVCGEN-ASGWHCGSITC	EACKK	FFLRSVNEEYR-----YR	CMKDKKC	IITRTTRTQCQYCRYMKCIEVGM	CAVCQHPSSGIHFGATTC	EGCKG	FFRRT-MK--ERTP-QRYK--	CPEKNNC	EVNTSTRSACRACRFQKCLTAGM
**As2DBD-NRA2**	CQVCGEN-ASGWHCGSITC	EACKK	FFLRSVNDEYR-----YK	CIKDKKC	PITRTTRTQCQYCRYTKCIEVGM	CAVCQKASSGIHFGATTC	EGCKG	FFRRT-IK--ERIP-QRYK--	CPEKNNC	EINYLTRSACRACRFQKCLTVGM
**Ar2DBD-NRA2a**	CQVCGEN-ASGWHCGSITC	EACKK	FFLRSVNDEYR-----YK	CMKDKNC	LITRTTRTQCQYCRYAKCIQVGM	CAVCHHASSGIHFGATTC	EGCKG	FFRRT-MR--ERIP-PRYK--	CAENNTC	VIGYLTRNSCRACRFEKCLRAGM
**Rs2DBD-NRA2a**	CQVCGEN-ASGWHCGSITC	EACKK	FFLRSVNEEYR-----YK	CIRDKKC	IITRTTRTQCQYCRYMKCIEVGM	CAVCQRPSSGIHFGATTC	EGCKG	FFRRT-IK--ERTP-QRYK--	CPENYNC	EVNSSTRSACRACRFQKCLAAGM
**Rm2DBD-NRA2a**	CQVCGEN-ASGWHCGSITC	EACKK	FFLRSVNEEYR-----YK	CIRDKKC	IITRTTRTQCQYCRYTKCIEVGM	CAVCQKPSSGIHFGATTC	EGCKG	FFRRT-IK--ERTP-QRYK--	CPEDYNC	EVNSSTRSACRACRFQKCLAAGM
**Rso2DBD-NRA2b**	CQVCGEN-ASGWHCGSITC	EACKK	FFLRSVNGEYL-----YK	CIRDKKC	IITRTTRTQCQYCRYSKCIAIGM	CAVCQSASSGIHFGATTC	EGCKG	FFRRM-IK--ERIP-QHYK--	CSERNNC	EVNALTRNMCRACRFRKCLMAGM
**Rs2DBD-NRA2b**	CQVCGEN-ASGWHCGSITC	EACKK	FFLRSVNGEYL-----YK	CIRDKKC	IITRATRTQCQCCRYSKCIAIGM	CAVCQSASSGIHFGATTC	EGCKG	FFRRM-TK--ENTP-QNYK--	CSERNNC	EINALTRNMCRACRFRKCLMAGM
**Rm2DBD-NRA2b**	CQVCGEN-ASGWHCGSITC	EACKK	FFLRSVNGEYL-----YK	CIRDKKC	IITRTTRTQCQCCRYSKCIAIGM	CAVCQSASSGIHFGATTC	EGCKG	FFRRM-TK--ENTP-QNYK--	CSERSNC	EINALTRNMCRACRFRKCLMAGM
**Rs1s2DBD-NRA2b**	CQVCGEN-ASGWHCGSITC	EACKK	FFLRSVNGEYL-----YK	CIRDKKC	IITRTTRTQCQYCRYSKCIAIGM	CAVCQSASSGIHFGATTC	EGCKG	FFRRM-IK--ERIP-QHYK--	CSEKNNC	EVNAITRNMCRACRFRKCLVAGM
**Rs1s2DBD-NRA2c**	CQVCGEN-ASGWHCGSITC	EACKK	FFLRSVNGEYL-----YK	CIRDKKC	IIARTTRTQCQYCRYSKCIAIGM	CAVCQSASSGIHFGATTC	EGCKG	FFRRM-IK--ERIP-QHYK--	CSEKNNC	EVNAITRNMCRACRFRKCLTAGM
**Ar2DBD-NRA2b**	CQVCGEN-ASGWHCGSITC	EACKK	FFLRSVNGEYL-----YR	CIRDKKC	PITRTTRTQCQFCRYSKCITIGM	CAVCTDASSGLHFGVTTC	ESCKG	FFRRM-IK--ERIP-QHYK--	CLERNNC	EVNTSTRNLCRACRFQKCLSVGM
**Dc2DBD-NRA2a**	CQVCSEQ-SSGWHCGAVTC	EACKK	FFLRSVNEEHL-----YK	CIRDKRC	QVTRSTRTQCQYCRYQKCIEVGM	CACCNAPSSGIHFGATTC	EGCKG	FFRRT-IK--ERTP-QRYK--	CLESNNC	EINTSTRNMCRACRFQKCVNAGM
**Dc2DBD-NRA2b**	CQVCSEQ-SSGWHCGAVTC	EACKK	FFLRSINEEYL-----YK	CVRDKKC	LITRSTRTQCQYCRYQKCIQVGM	CSCCTAPSSGIHFGAMTC	EGCKG	FFRRT-MK--ERTS-QRYT--	CLESNNC	EINTSTRNICRACRFQKCINAGM

**Ar2DBD-NRA3b**	CQICGDL-SSGWHCGAITC	EACKK	FFLRSISTGDG-----YK	CQRDFSC	AITKRSRTQCQYCRFQKCLAVGM	CLVCGDPSSGIHFGAVTC	EACKG	FFRRS-MR--ENAV-ERYR--	CAENNNC	EIRSGSKVTCRACRFRKCVQKGM
**Ar2DBD-NRA3a**	CQICGDL-SSGWHCGAITC	EACKK	FFLRSISTGDG-----YK	CQRDFSC	AITKRSRTQCQYCRFQKCLAVGM	CLVCGGSASGIHFGAVTC	EACKG	FFRRS-IK--ENAP-DRYR--	CTENNNC	DIHATSKITCRACRFKKCVQAGM
**As2DBD-NRA3a**	CQICGDL-ASGWHCGAITC	EACKK	FFLRSISTGDG-----YK	CQKDFSC	SITKRSRTQCQYCRFQKCISMGM	CLVCSASASGIHFGAVTC	EACKG	FFRRS-IK--ENAP-ERYR--	CAENNNC	EILSTSKITCRACRFKKCVDAGM
**As2DBD-NRA3b**	CQICGDL-ASGWHCGAITC	EACKK	FFLRSISTGDG-----YK	CQKDFSC	SITKRSRTQCQYCRFQKCISMGM	CLVCSASASGIHFGAVTC	EACKG	FYRRS-IK--ENAA-ERYL--	CVENNSC	EILAPSKLSCRACRFKKCIHFGM
**Dc2DBD-NRA3a**	CQICCDT-ASGWHCGAITC	EACKK	FFLRSITTGDG-----YK	CQRDQCC	LINKRSRTQCQFCRFQKCLKAGM	CLVCGAPASGIHFGAVTC	EACKG	FFRRS-IK--ENAP-DRYR--	CAENNNC	EINSTSKITCRACRFRKCINAGM
**Rm2DBD-NRA3b**	CKICGDL-ASGWHCGAITC	EACKK	FFLRSISTCNG-----YK	CQRDLKC	LVTKRSRTQCQYCRFQKCISVGM	CLICNASASGIHFGAVTC	EACKG	FFRRS-IK--ENAP-ERYH--	CTENNNC	EIISTSKITCRACRFRKCIEAGM
**Rs2DBD-NRA3b**	CKICGDI-ASGWHCGAITC	EACKK	FFLRSISTCNG-----YK	CQRDLNC	LVTKRSRTQCQYCRFQKCISVGM	CLICNASASGIHFGAVTC	EACKG	FFRRS-IK--ENAP-ERYH--	CAENNNC	EIISTSKITCRACRFRKCIEAGM
**Rs1s2DBD-NRA3b**	CHICGDL-ASGWHCGAITC	EACKK	FFLRSISTGDG-----YK	CQRDFRC	SITKRSRTQCQYCRFQKCISVGM	CLICNASASGIHFGTVTC	EACKG	FFRRS-IK--ENAP-ERYH--	CVENNNC	EIISTSKITCRACRFRKCIEAGM
**Rs2s2DBD-NRA3c**	CQVCGDL-ASGWHCGAITC	EACKK	FFLRSISTCVG-----YK	CPRDFRC	SITKRSRTQCQYCRFQKCISAGM	CLICNASASGIHFGTVTC	EACKG	FFRRS-IK--ENAP-ERYH--	CTENNNC	EIISTSKITCRACRFRKCIEAGM
**Rso2DBD-NRA3b**	CQICGDL-ASGWHCGAITC	EACKK	FFLRSISTYDG-----YK	CQKELRC	SITKRSRTQCQYCRFQKCISVGM	CLICKASASGIHFGAVTC	EACKG	FFRRS-IK--ENAP-ERYH--	CTENNNC	EIISTSKITCRACRFRKCIEAGM
**Rs2s2DBD-NRA3b**	CQICGDL-ASGWHCGAITC	EACKK	FFLRSISTCDG-----YI	CQRNSTC	SITKRSRTQCQYCRLQKCISVGM	CLICNASASGIHFGVVTC	EACKG	FFRRS-IK--ENAP-ERYY--	CVENNNC	EIISTSKITCRACRFRKCIEAGM
**Rso2DBD-NRA3a**	CQICGDL-ASGWHCGAITC	EACKK	FFLRSISTNDE-----YK	CQKNLNC	SITKRSRTQCQYCRLQKCISVGM	CVICNASASGIHFGAVTC	EACKG	FFRRS-IK--ENAL-ERYH--	CIENNNC	EIISTSKITCRACRFRKCIQTGM
**Rs2s2DBD-NRA3a**	CQICGDL-ASGWHCGAITC	EACKK	FFLRSISTSDE-----YK	CQRNLNC	SITKRSRTQCQYCRLQKCISVGM	CLICNASASGIHFGAVTC	EACKG	FFRRS-IK--ENAP-ERYH--	CTENNNC	EISTASKITCRACRFRKCIEAGM
**Rs1s2DBD-NRA3a**	CQICGDL-ASGWHCGAITC	EACKK	FFLRSISTCDG-----YK	CQRDSRC	SITKRSRTQCQYCRFQKCITIGM	CVICNASASGLHFGAVTC	EACKG	FFRRS-IK--ENAT-ERYH--	CAENNNC	EIVSTSKIICRACRFRKCIEAGM
**Rm2DBD-NRA3a**	CQVCGDL-ASGWHYGAITC	EACKK	FFLRSISPCNG-----LK	CQRNLSC	SMTKRSRTQCQYCRLQKCISVGM	CVICNASASGIHFGAVTC	EACKG	FFRRS-IK--ENAP-DRYY--	CTENNNC	EIVSTSKTTCRACRFRKCIEAGM
**Rs2DBD-NRA3a**	CQVCGDL-ASGWHYGAITC	EACKK	FFLRSISPCNG-----LK	CQRNLSC	SMTKRSRTQCQYCRLQKCISVGM	CVICNASASGIHFGAVTC	EACKG	FFRRS-IK--ENAP-DRYY--	CTENNNC	EIVSTSKTTCRACRFRKCIEAGM
**Dc2DBD-NRA3b**	CQICTDK-ASGWHCGAITC	EACKK	FFLRTINAEN------YK	CQHDKCC	IINRHSRTQCQYCRYQKCLKFGM	CIICSSSSSGLHFGAITC	EACKC	FFRRS-IK--ENAI-EHYH--	CSANDCC	KIDSKLKMNCRACRFRKCIDGGM
**Ar2DBD-NRA1**	CQVCGEQ-SSGLHCGAITC	EACKK	FFLRSINGEDQ-----YK	CVKNKDC	MITRNTRTQCQYCRFQKCLMIGM	CYVCQGPSSGIHFGAITC	EGCKG	FFRRS-IK--ERAP-SRYK--	CMDNGTC	EMSVSTRNACRYCRFQRCIKVGM
**As2DBD-NRA1a**	CQVCGEQ-SSGLHCGAITC	EACKK	FFLRSINGEDQ-----YK	CVKNKDC	MITRNTRTQCQYCRFQKCRIIGM	CYVCQAPSSGIHFGAITC	EGCKG	FFRRS-IK--ERAP-SRYK--	CMDNGTC	EMTVATRNACRYCRFQRCIKVGM
**Rso2DBD-NRA1**	CQVCGEQ-SSGLHCGAITC	EACKK	FFLRSINGEDQ-----YK	CVRNKDC	IITRNNRTQCQYCRFQKCKIIGM	CYVCQAPSSGIHFGAITC	EGCKG	FFRRS-IK--ERAP-SRYK--	CMDNGTC	EINVATRNACRYCRFQRCIKVGM
**Rs2DBD-NRA1b**	CQVCGEQ-SSGLHCGAITC	EACKK	FFLRSINGEDQ-----YK	CVRNKDC	IITRNNRTQCQYCRFQKCKIIGM	CYVCQAPSSGIHFGAITC	EGCKG	FFRRS-IK--ERAP-SRYK--	CMDNGTC	EINVATRNACRYCRFQRCIKVGM
**Rs1s2DBD-NRA1a**	CQVCGEQ-SSGLHCGAITC	EACKK	FFLRSINGEDQ-----YK	CVRNKDC	IITRNNRTQCQYCRFQKCKTIGM	CYVCQAPSSGIHFGAITC	EGCKG	FFRRS-IK--ERAP-SRYK--	CMDNGTC	EINVATRNACRYCRFQRCIKVGM
**Rm2DBD-NRA1**	CQVCGEQ-SSGLHCGAITC	EACKK	FFLRSINGEDQ-----YK	CVRNKDC	IITRNTRTQCQYCRFQKCKIIGM	CYVCQAPSSGIHFGAITC	EGCKG	FFRRS-IK--ERAP-SRYK--	CMDNGTC	EINVATRNACRYCRFQRCIKVGM
**Rs2DBD-NRA1**	CQVCGEQ-SSGLHCGAITC	EACKK	FFLRSINGEDQ-----YK	CVRNKDC	IITRNTRTQCQYCRFQKCKIIGM	CYVCQAPSSGIHFGAITC	EGCKG	FFRRS-IK--ERAP-SRYK--	CMDNGTC	EINVTTRNACRYCRFQRCIKVGM
**Ar2DBD-NRA1b**	CQVCGEQ-SSGLHCGAITC	EACKK	FFLRSINGEDL-----YK	CVRSKDC	VITRNTRTQCQYCRFQKCKFVGM	CYVCQASSSGIHFGAITC	EGCKG	FFRRS-IK--ERAP-SRYK--	CMDNGTC	EISASTRNGCRYCRFQRCIKVGM
**As2DBD-NRA1b**	CQVCGEQ-SSGLHCAAITC	EACKK	FFLRSINGEDL-----YK	CVRNKEC	MITRNTRTQCQYCRFQKCKLIGM	CYVCQAPSSGIHFGAITC	EGCKG	FFRRS-IK--ERAP-SRYK--	CMDNGTC	EINASTRNGCRYCRFQRCIKVGM
**Bc2DBD-NRA1a**	CKVCGEP-SSGWHCGAVTC	EACKK	FFLRSINGEDA-----YK	CIRNQDC	VIVRTTRTQCQFCRFQKCKEVGM	CAVCKAPSSGIHFGAITC	EGCKG	FFRRS-IK--ERAP-ERYR--	CMENGNC	EISAATRNMCRFCRFQKCLKAKM
**Bp2DBD-NRA1**	CKVCGEP-SSGWHCGAVTC	EACKK	FFLRSINGEDA-----YK	CIRNQDC	VIVRTTRTQCQFCRFQKCKEVGM	CAVCKAPSSGIHFGAITC	EGCKG	FFRRS-IK--ERAP-ERYR--	CMENGTC	EISAATRNMCRYCRFQKCLKAKM
**Bc2DBD-NRA1b**	CKVCGEP-SSGWHCGAVTC	EACKK	FFLRSINGEDA-----YK	CIRNKDC	VIVRTTRTQCQFCRFQKCKEVGM	CYVCKSPSSGIHFGAITC	EGCKG	FFRRS-VK--ERAP-ERYR--	CLENGNC	EISASTRNMCRFCRFQKCISVGM
**Bc2DBD-NRA4a**	CRVCGETNSAGWHCGTITC	EACKK	FFLRNVKGDYL-----LK	CIRNSSC	VITKSTRTVCGYCRFQKCFQVGM	CSVCGDSSSGLHFGVITC	EGCKG	FFRRN-IK--L--G-HTFS--	CSSNGDC	EIGYKTRNACRSCRYKKCISAGM
**Bp2DBD-NRA4**	CRVCGETNSAGWHCGTITC	EACKK	FFLRNAKSEYL-----FK	CVRNSSC	VITKSTRTNCAFCRFKKCLQVGM	CSVCGDASSGLHFGAITC	EGCKG	FFRRN-VK--E--G-HKFV--	CTGDGNC	EISYKSRNACRSCRYNKCVTAGM
**Bc2DBD-NRA4b**	CRICGETGTTGWYCGTITC	EACKK	FFMRSVKSDYL-----LK	CVRNSNC	VITKSTRTNCGHCRFQKCLQVGM	CSVCGDASSGFHFGAFTC	EGCKG	FFLRYKNK--K--I-TNES--	CPNRNIC	QINFSSRNQCKSCRFHKCLTVGM
**Bc2DBD-NRA5**	CQVCGDD-NCNWIYGAMIC	EACKK	FFIRSIKEEKR-----YV	CIANKKC	SITKSTRANCQYCRYKKCLDVGM	CVICEQKPSGIHFGVMSC	EACKG	FFRRSCLN--NAS--KQYK--	CKFDGNGNC	Q---STSRSCRFCRLKKCIEVGM
**Bc2DBD-NRA5**	CKVCGDE-ACNWYYGAMVC	EACKK	FFIRSKKEQKR-----YI	CVANKQC	NITKSTRANCQYCRFKKCVQVGM	CAVCSQQPSGIHFGVMSC	EACKG	FFRRSALV--HSD--NPLK--	CKSNGNQNC	L---LTNRSCRYCRFKKCLEAGM
**Ap2DBD-NRB**	CAVCGDQ-ATGRYFGAQIC	EACKS	FFIRSTKKGMP-----FK	CQSSGTC	DVTPTSRLLCQYCRFQKCLVAGM	CKVCGDTSSGIHFGVFTC	EGCKG	FFRRS-LK--DG---ASYV--	CGDNKKC	IITPTSRNVCRYCRYQKCLQVGM
**Pmi2DBD-NRB**	CAVCEDQ-ATGRYFGAQIC	EACKS	FFIRSTKKGMP-----FK	CQSSSAC	PVTPTSRLLCQHCRFQKCLNAGM	CKVCNDMSSGIHFGVFTC	EGCKG	FFRRS-LK--DG---ASYM--	CGDEKKC	VITPTSRNVCRYCRYQKCLQVGM
**Aru2DBD-NRB**	CAVCGDQ-ATGRYFGAQIC	EACKS	FFIRSTKKGTP-----FK	CQSSGHC	KVTPTSRLLCQYCRFQKCLTAGM	CQVCGDTSSGIHFGVFTC	EGCKG	FFRRS-LK--DG---TSYF--	CIDDKKC	LITPTSRNMCRFCRYQKCLQVGM
**Lv2DBD-NRB**	CAVCGDQ-ATGRYFGAQIC	EACKS	FFIRSTRKGEP-----FR	CVNNQDC	HITPFSRLLCQYCRYQKCMKAGM	CKVCGDVSSGIHFGVYTC	EGCKG	FFRRS-LR--DR---NTYI--	CSGKEEC	IITSVTRNHCRYCRFKKCLSVGM
**Spur2DBD-NRB**	CAVCGDQ-ATGRYFGAQIC	EACKS	FFIRSTRKGEP-----FR	CVNNQSC	PITPYSRLLCQFCRYQNCMKAGM	CKVCGDVSSGIHFGVYTC	EGCKG	FFRRS-LR--DR---NTYS--	CSGKEEC	IITPVTRNHCRYCRFKKCLRVGM
**Aja2DBD-NRB**	CVVCGDQ-AAGKYFGALIC	EACKS	FFIRSTKKGEP-----FK	CSNNGLC	TITPTSRLLCQCCRYQKCLEVGM	CKVCNDVSSGIHFGVYTC	EGCKG	FYRRS-LR--DS---SNYT--	CVDNKQC	NITPSTRNVCRYCRFQKCLEVGM
**Cg2DBD-NRB1**	CVVCGER-ASGYYFGALVC	LPCKS	FYIRCTKDGEPT----FT	CQCNGNC	DIAKQGRIRCQYCRYQRCLMAGM	CKVCGDIANGIHFGVNTC	EGCKK	FFRRG-LV--EN---QSYL--	CKSEKKC	TINPRNRNNCRYCRYQKCISVGM
**Cv2DBD-NRB1**	CVVCGER-ASGYYFGALVC	LPCKS	FYIRCTKDGEPT----FT	CQCNGNC	DIAKQGRIRCQYCRYQRCLMAGM	CKVCGDIANGIHFGVNTC	EGCKK	FFRRG-LV--EN---QSYL--	CKSEKKC	TINPRNRNNCRYCRYQKCISVGM
**My2DBD-NRB1**	CVVCGEK-ASGYYFGALVC	LPCKS	FYIRCTKEGEPT----FT	CQCNGNC	DIAKQGRIRCQYCRYQRCLMAGM	CKVCGDIANGIHFGVNTC	EGCKK	FFRRG-LV--EN---QSYV--	CKGDKKC	TINPRNRNNCRFCRYQKCITVGM
**Pm2DBD-NRB1**	CVVCGEK-ASGYYFGALVC	LPCKS	FYIRCTKEGEPT----FT	CQCNGNC	DIAKQGRIRCQYCRYQRCLMAGM	CKVCGDIANGIHFGVNTC	EGCKK	FFRRG-LV--EN---QSYV--	CKGDKKC	TINPRNRNNCRFCRYQKCITVGM
**Mc2DBD-NRB1**	CVVCGEK-ASGFYFGALVC	LPCKS	FYIRCTKDGEPT----FT	CQCNGNC	DIAKQGRIRCQYCRYQRCLMAGM	CKVCGDIANGIHFGVNTC	EGCKK	FFRRG-LV--EN---QGYN--	CKGEKSC	QINPRNRNNCRYCRYQKCISAGM
**MgMR7B1**	CVVCGEK-ASGFYFGALVC	LPCKS	FYIRCTKDGEPT----FT	CQCNGNC	DIAKQGRIRCQYCRYQRCLMAGM	CKVCGDIANGIHFGVNTC	EGCKK	FFRRG-LV--EN---QGYN--	CKGEKSC	QINPRNRNNCRYCRYQKCISAGM
**Me2DBD-NRB1**	CVVCGEK-ASGFYFGALVC	LPCKS	FYIRCTKDGEPT----FT	CQCNGNC	DIAKQGRIRCQYCRYQRCLMAGM	CKVCGDIANGIHFGVNTC	EGCKK	FFRRG-LV--EN---QGYN--	CKGEKSC	QINPRNRNNCRYCRYQKCISAGM
**Mm2DBD-NRB1**	CVVCGER-ASGFYFGALVC	LPCKS	FYIRCTKDGEPT----FT	CQCSGNC	DIVKQGRIRCQYCRYQRCLMAGM	CKVCGDIANGIHFGVNTC	EGCKK	FFRRG-LV--EN---QSYI--	CKGEKEC	GINPRNRNNCRYCRYQKCLTVGM
**Po2DBD-NRB3**	CDVCGEQ-ASGHYFGALVC	LPCKS	FFIRCTKDGRPT--FSTQ	C--GGKC	DILKGGRVRCQHCRFQKCINAGM	CKVCGDIANGIHFGVTTC	EGCKK	FFRRG-LK--EN---RSYT--	CKGSMHC	SINPRMRNNCRYCRYQKCLLEGM
**Ema2DBD-NRB3**	CDVCGEQ-ASGHYFGALVC	LPCKS	FFIRCTKDGRPS--FSSQ	C--GGKC	DVLKGGRVRCQHCRFQRCISAGM	CKVCGDIANGIHFGVTTC	EGCKK	FFRRG-LK--EN---RSYT--	CKGSMHC	SINPRMRNNCRYCRYQKCLLEGM
**Ech2DBD-NRB3**	CDVCGEQ-ASGHYFGALVC	LPCKS	FFIRCTKDGRPS--FSSQ	C--GGKC	DVLKGGRVRCQHCRFQRCISAGM	CKVCGDIANGIHFGVTTC	EGCKK	FFRRG-LK--EN---RSYT--	CKGSMHC	SINPRMRNNCRYCRYQKCLLEGM
**Bg2DBD-NRB3**	CDVCGEQ-ASGHYFGALVC	LPCKS	FFIRCTKDGRPN--FSNQ	C--GGKC	DVLKGGRVRCQHCRFQKCIHAGM	CKVCGDIANGVHFGVTTC	EGCKK	FFRRG-LK--EN---KSYT--	CKANMHC	SINPRMRNNCRYCRYQKCLYEGM
**Bt2DBD-NRB3**	CDVCGEQ-ASGHYFGALVC	LPCKS	FFIRCTKDGRPN--FSNQ	C--GGKC	DVLKGGRVRCQHCRFQKCISAGM	CKVCGDIANGVHFGVTTC	EGCKK	FFRRG-LK--EN---RTYT--	CKASQMC	SINPRMRNNCRYCRYKKCITEGM
**Aca2DBD-NRB3**	CDVCGEQ-ASGHYFGALVC	LPCKS	FFIRCTKDGHPN--FSNQ	C--GGKC	DVLRGGRVKCQHCRFQKCISAGM	CKVCGDIANGVHFGVTTC	EGCKK	FFRRG-LK--EN---RSYT--	CKGSMRC	SINPRMRNNCRYCRYQKCLFEGM
**Pca2DBD-NRB3**	CDVCGEP-ASGHYFGALVC	LPCKS	FFIRCTKTGDPV--FSSQ	C--GGTC	DVHKLGRIRCQFCRFQRCLSAGM	CKVCCDIANGVHFGVTTC	EGCKK	FFRRG-LK--EH---QAYV--	CKVAKKC	TINPRMRNNCRYCRFQKCLNVGM
**Pvd2DBD-NRB3**	CDVCGEP-ASGHYFGALVC	LPCKS	FFIRCTKTGDPV--FSSQ	C--GGTC	DVHKLGRIRCQFCRFQRCLSAGM	CKVCCDIANGVHFGVTTC	EGCKK	FFRRG-LK--EH---QAYV--	CKVAKKC	TINPRMRNNCRYCRFQKCLNVGM
**Po2DBD-NRB2**	CVVCHEA-ASGNFFGAVVC	LPCKS	FFIRCTKDSESC--IVRQ	C--RGQC	DISKQLRNRCQFCRYQRCLAAGM	CRVCGDLSNGIHFGVFTC	EGCKK	FFRRG-LK--EH---TTYV--	CKEHGMC	RLTPRNRNSCRRCRYRKCIDVGM
**Aca2DBD-NRB2**	CVVCGEV-ANGNFFGAVVC	LPCKS	FFIRCTKDGEPC--ILQQ	C--GGNC	DIKKQLRNRCQYCRYQQCLAMGM	CKVCGDLANGIHFGVYTC	EGCKK	FFRRG-LK--EH---LSYV--	CKDAKRC	RLNPRNRNNCRYCRYQKCLLVGM
**Hruf2DBD-NRB2**	CQVCGEK-ASGNFFGALVC	LPCKS	FFIRCTKEGEPV--FVSQ	C--DSRC	DVSKQGRNRCQHCRYKKCLAAGM	CRVCGDIANGIHFGVYTC	EGCKK	FFRRG-LL--EY---QSYV--	CKGAMSC	LLNPRNRNNCRYCRYQKCLNVGM
**Hrub2DBD-NRB2**	CQVCGEK-ASGNFFGALVC	LPCKS	FFIRCTKDGEPV--FVSQ	C--DGRC	DVSKQGRNRCQNCRYKKCLAAGM	CRVCGDIANGIHFGVYTC	EGCKK	FFRRG-LL--EY---QSYV--	CKGAMLC	VLNPRNRNNCRYCRYQKCLNVGM
**Pca2DBD-NRB2**	CAVCGDR-ASGNFFGATVC	LPCKS	FFIRCTKDGEPA--VQKQ	C--NGCC	DVSKELRNRCQFCRFQRCLLIGM	CRVCGDLANGVHFGIFTC	EGCKK	FFRRG-LK--EH---KSYV--	CRGAHDC	QLNPRNRNNCRACRFWKCAAVGM
**Ga2DBD-NRB1**	CAVCGEE-ANGNFFGALVC	LPCKS	FFIRCNKANELTS-VQRP	C--DNRC	TATRQGRVRCQRCRYLKCVQVGM	CLVCGDIANGIHFGIKTC	EGCKK	FFRRG-LL--EN---KTYV--	CTKQQAC	IINPRNRNNCRLCRYRKCVSSGM
**Lg2DBD-NRB1**	CVVCKEP-AHGYNFGALVC	LPCKS	FYIRCSKEDVIY----SK	C--RGQC	DITGENRIRCQYCRYQQCLKVGM	CMVCGDLSNGIHFGVGTC	EGCKK	FFRRC-LL--ES---SKLI--	CVNERRC	KINPKTRNRCRLCRYLKCLQVGM
**Hruf2DBD-NRB1**	CVVCADN-ASGQYFGATVC	LPCKS	FYIRCTKEGEPV--FSSK	C--HGSC	DVTKQARIRCQSCRYQKCLKAGM	CLVCGDLANGIHFGVYTC	EGCKK	FFRRG-LV--EN---RTYQ--	CKGDNTC	VINPRSRNTCRACRYRKCLSVGM
**Pau2DBD-NRB**	CSVCGAD-ASGMYFGAIVC	LPCKS	FFIRCTKEGEPTR---LK	CQNDINC	GYPGPIRHRCQYCRFQRCLVAGM	CKVCGDIANGIHFGVNTC	EGCKK	FFRRG-LK--EA---DTYA--	CKEGQAC	PITPRSRNACRFCRYMKCVTVGM
**Ct2DBD-NRB1**	CVVCEDK-AEGRYFGATVC	LPCKS	FFIRCTKEGPKR----LV	CHATKNC	DMTGGNRMRCSFCRHQKCLSMGM	CRVCSDIANGVHFGVTTC	EGCKK	FFRRA-LK--EY---RTYR--	CKFNLMC	TVNPRNRNTCRSCRYQKCIRLGM
**Ls2DBD-NRB1**	CAVCSEK-ANGRYFGAIVC	LPCKS	FFIRCTKDGKPT----LS	CPENMAC	DITGVNRMRCQYCRYKKCLSVGM	CRVCLDIANGIHFGVTTC	EGCKK	FFRRA-LK--EY---LMYD--	CKFLKHC	FINPRNRNSCRYCRYKKCLGVGM
**La2DBD-NRB**	CTVCDEP-ASGIYFGALVC	LACKS	FYIRCTREGHKTD---YR	CAAVGRC	LLDKPYRVRCQQCRFQKCLSVGM	CKVCGYMANGVHFGVETC	EGCKK	FFRRG-LK--EC---ATYH--	CKALKDC	AINPKNRNDCRYCRYQKCLAVGM
**Hr2DBD-NRB2**	CVVCGDS-AHGNYFHAFVC	VPCKT	FFLRYADEHAQ-----FQ	CKFNGNC	EITLTTRTNCKHCRYQKCVKQGM	CVVCGDLANGIHFGANTC	EGCKK	FFRRG-LT--ES---GSYS--	CKNNHDC	KINPNTRNNCRFCRFKKCLDVGM
**Ls2DBD-NRB2a**	CKVCSDT-ATGMYFGALVC	VPCKT	FFLRYAGDNFA-----FR	CKRDKGC	EIVTATRTQCKYCRYQKCLHVGM	CVVCLDLANGVHFGAVTC	EGCKK	FFRRG-LT--EH---ESYQ--	CKLDKSC	PINPRTRNACRYCRYQKCINVGM
**Ls2DBD-NRB2b**	CQVCGEK-ASGNFFGALVC	LPCKT	FFIRCSSEEVST----FR	CRVDFNC	EVTGSRRSKCKYCRYRKCIAVGM	CVVCHDVANGVHFGIVSC	EGCKK	FFRRG-LQ--ES---ESYI--	CKQNGAC	VINPKTRNDCRYCRFQKCLMAGM
**Ct2DBD-NRB2**	CQVCNDH-AAGTYFGAKVC	IPCKA	FFIRSTSDKTSV----FE	CPGDQRC	KITVMTRTACKFCRFQNCLKAGM	CVICGDDANGVHFGALTC	EGCKK	FYRRG-LLVGEA---KSYI--	CKADKTC	EITARTRNNCRYCRFQKCLRMGM
**Of2DBD-NRB2**	CSVCGSR-AAGVFFGVLTC	WTCKT	FFIRHQKAGAGG----LK	CDNNGQC	KLDLKLRRRCAYCRYKKCIEVGM	CLVCGDIASGVHFGIVAC	LGCTK	FFRRT-IRMGTE---DSYM--	CYNAHRC	VINPQNRNTCPRCRLDKCKRLGM

```
Cb2DBD-NRC     CKVCEKP-SNGLHYGVRAC DSCKC FFARSLKRSTES----LV CSKDSNC NFGRQPKTICSYCRFQKCVKVGM CKVCEKPGHAIHYGVWTC DSCKW FFTNS-LR---RGT-ESLF-- CHKGSSC   DLSSNPFETCKYCRFQKCVKLGM
Cr2DBD-NRC1    CKICGGY-ARGVNYGVLSC DNCRA FFSHYLHRKK------LK CRKNNNC VIDKFTSNKCGKCRMEKCLRMGM CKVCGDVPVGIGYGVLSC GSCRM FFRRH------NG--TDVHLS CNKEGKC   DL-RLKFAKCPKCRMRKCLEVGM
Cr2DBD-NRC2    CKICGGY-AKGTNYGVLTC DGCRT FFIQYIHMKE------LK CRRNNNC VIDKFITKKCAKCKIEKCLIMGM CKVCGDTASKIFYGVLTC EGCRV FFRRY------AG--KDVKLM CYSRGNC   NL-LEKFTKCQKCRMRKCLEVGM
Cr2DBD-NRC3    CKVCGDK-PRGVHYGVLAC EGCRS FFRTNRDKKE------LK CRKSNKC VVDKYSRNKCGKCRMEKCLKLGM CRVCGDISFEHFFDIVAC EGCKG FFRTH------HE--KDIKLE CRVDGNC   DINIISRNTCKSCRMKKCLEAGM
Cr2DBD-NRC4    CNICGDV-ANGVHYGVPAC EGCKK FFSRMHNKQEY-----FL CRKGNNC FIDRESRNSCQSCRLAKCRQAGM CEVCGDERYSTNRVPAYC EACKT VYSSH------IK--EIKMFQ CEKDGKC   VINKWTRDKCQSCRMGKCLEAGM
Aa2DBD-NRC3    CVICADL-ANAYHYGVASC NGCKT FFRRTIVDGHAD----LR CQFEGHC EVSKEIRNACRRCRFDKCLQAGM CLICSDVATGYHYGVTSC HGCKA FFRRTIIA---G---RTF--V CDRDGTC   AVTKGDPTFCRGCRLTKCLEAGM
Aa2DBD-NRC4    CVICADL-ANAYHYGVASC NGCKT FFRRTIVDGHAG----LR CQFEGRC EVSKDGRNACRYCRFNKCLQAGM CLICSDVATGYHYGVASC HGCKA FFRRSIIA---G---RTF--V CDRGGAC   AVMKGDPTFCRGCRLTKCLEAGM
Aa2DBD-NRC5    CAICSDL-ANAYHYGVASC NGCKT FFRRTIVDGHAG----LR CQFEGRC EVSKEVRNACRRCRFDKCVLAGM CLICSDFATGYHFGASSC HGCKS FFRRTIVA---G---RNF--V CERDGTC   AVQKRDSTSCRGCRFNKCLEAGM
Aa2DBD-NRC2    CAICNDI-ASGYHFGAASC AGCRI FFRRNVLSGDSE----LR CHYEGNC EVSKDVRTACRHCRFNKCLLAGM CAICSDVATGYHYGVAAC FGCKT FFRRTVVA---G---HTY--I CQHGGTC   VVNKDQRMACRHCRLNKCIQAGM
Aa2DBD-NRC1    CAICDDT-ASGTHYGVTSC TGCKT FFRRTIVDGHAE----LR CQYEGHC VVSKDVRNACRQCRFDKCLLAGM CAICEDVATGYHYGVAAC LGCKA FFRRTIVA---G---HAY--T CQRDGNC   IVNVDGRMACRHCRFNKCLQAGM
Aa2DBD-NRC6    CAICDGT-ASGYHYDVASC TACWM FFRQAVVSGEAE----LR CEYQGNC IVSKDVRNACRHCRFNKCLLEGM CAICDDVANGYHYGVASC LGCKG FFRRTIVS---G---DAARIR CQHDGNC   DVNKRNRNACRRCRFDKCQLAGM
Aa2DBD-NRC7    CVICDAT-ASGYHYDVASC TACWM FFRQMVITGEAE----LR CQRQGNC VVSKDVRNACRHCRFNKCLLEGM CAICHDAATGYHYGVAAC HACKT FFRRVIVM---G---DSARIR CQKDGNC   DVSKRNRNACRRCRFDKCQQAGM
```
