# Supplementary material for: Updated knowledge and a proposed nomenclature for nuclear receptors with two DNA binding domains (2DBD-NRs)
Source: PLoS One. 2023 Sep 12;18(9):e0286107. doi: 10.1371/journal.pone.0286107 (PMC10497141; doi:10.1371/journal.pone.0286107)
Supplement: S3 File — (PDF) [file pone.0286107.s003.pdf]

Supplemental material 3. Ti sequence for sequence logo

(NCBI accession number of 2DBD-NRs see table 6, accession number of other NR is in the bracket after each NR name)

2DBD-NRA

```
Bf2DBD-NRA                 FAK-KIPGFRTCSID-DQISMIQ
Bf2DBD-NRA                 FAK-KIPGFRTCSID-DQISMIQ
OfuNR7A1                   YAK-KIPGFREFPVE-DQILLLQ
Sb2DBD-NRA2                FAK-LVPGFNQLGIT-ARSNLVR
Sm2DBD-NRA2                FAK-LVPGFNQLGIT-ARSNLVR
Sj2DBD-NRA2                FAK-LVPGFNQLGIT-ARSNLVR
Of2 BD-NRA2                FAK-LVPGFNQLGIT-ARSNLVR
Ov2DBD-NRA2                FAK-LVPGFNQLGIT-ARSNLVR
Cs2DBD-NRA2                FAK-LVPGFNQLGIT-ARSNLVR
Pw2DBD-NRA2                FAK-LVPGFNHLGIA-ARSNLVR
Pv2DBD-NRA2                FAK-LVPGFNHLGIA-ARSNLVR
Ph2DBD-NRA2                FAK-LVPGFNHLGIA-ARSNLVR
Psm2DBD-NRA2               FAK-LVPGFNHLGIA-ARSNLVR
Fh22DBD-NRA2               FAK-LVPGFNHLGIT-ARSNLVR
Eg2DBD-NRA2                FAK-LIPGFNQMSLS-ARGHLVR
Em2DBD-NRA2                FAK-LIPGFNQMSLS-ARGHLVR
Hm2DBD-NRA2                FAK-LIPGFNQMSLS-ARGHLVR
Eg2DBD-NRA2                FAK-LIPAFNQMSLS-ARGHLVR
Ta2DBD-NRA2                FAK-LIPGFNQMSLS-ARGHLVR
Hd2DBD-NRA2                FAK-LIPGFNQMSLS-ARGHLVR
Mc2DBD-NRA2                FAK-LIPGFNQLSLA-GRGNLVR
Se2DBD-NRA2                FAK-LIPGFNQLELT-ARGALVR
Sme2DBD-NRA3               FVK-AIPGFNKFELK-SRRSLVQ
Sj2DBD-NRA3                FVK-YIPGFCYLKIS-DQRQLVR
Sm2DBD-NRA3                FVK-YIPGFCYLKIS-DQRQLVR
Ph2DBD-NRA3                FVK-LIPGFNQLVLD-DRRQLVR
Pw2DBD-NRA3                FVK-LIPGFNQLVLD-DRRQLVR
Of2DBD-NRA3                FVK-LIPGFDQLELG-DKRQLVR
Ov2DBD-NRA3                FVK-LIPGFDQLELG-DKRQLIR
Em2DBD-NRA3                FSK-LIAGFNRLGIN-DRRQLIR
Eg2DBD-NRA3                FSK-LIAGFNRLGIN-DRRQLIR
Ht2DBD-NRA3                FSK-LIAGFNRLGIN-DRRQLIR
Ts2DBD-NRA3                FSK-LIAGFNRLGIN-DRRQLIR
Hs2DBD-NRA3                FSK-LIAGFNGLGIN-DRRQLIR
Hm2DBD-NRA3                FSK-LIAGFNGLGIN-DRRQLIR
Spr2DBD-NRA3               FTK-IIPGINRLELN-DKRQLVR
Sme2DBD-NRA3               FAK-AVPGFKELFHN-DMKVLVQ
Sj2DBD-NRA1                FAQ-TISSCQELSEF-DMKILIQ
Sj2DBD-NRA1                FAQ-TISSCQELSEF-DMKILIQ
Sma2DBD-NRA1               FAQ-SIPSFQELSQY-DMKILIQ
Sh2DBD-NRA1                FAQ-SIPSFQDLSEY-DMKILIQ
Sm2DBD-NRA1                FAQ-SIPNFQELSEF-DMKILIQ
Fb2DBD-NRA1                FAR-AVPGFRDLSRM-DMKTLVQ
Fh2DBD-NRA1                FAR-AVPGFRDLSRL-DMKTLVQ
Pw2DBD-NRA1                FAR-AVPGFRDLSRL-DMKTLVQ
Pw2DBD-NRA1                FAR-AVPGFRDLSRL-DMKTLVQ
Cs2DBD-NRA1                FAR-AVPGFRDLPSV-VMKKLVQ
Of2DBD-NRA1                FAR-AVPGFRELPSV-VMKKLVQ
Em2DBD-NRA1                FAR-AIPGFCDLPRA-DTKFLLQ
Eg2DBD-NRA1                FAR-AIPGFCDLPRA-DTKFLLQ
Hm2DBD-NRA1                FAR-AIPGFRDLPRA-DTKFLVQ
Hn2DBD-NRA1                FAR-AIPGFRDLPRA-DTKFLVQ
Hd2DBD-NRA1                FAR-AIPGFRDLPRA-DTKFLVQ
Mc2DBD-NRA1                FAR-AIPGFRDLPRA-DTKFLVQ
Ts2DBD-NRA1                FAR-AIPGFRDLPRA-DTKFLVQ
Sp2DBD-NRA1                FAR-AIPGFRDLPRS-DTKVLVQ
Cv2DBD-NRA                 ALK-MFPVFKILELD-DRITLVQ
Cg2DBD-NRA                 ALK-MFPVFKILELD-DRITLVQ
Pm2DBD-NRA                 FSK-KVPGFRALSLD-DQIKLVQ
Mc2DBD-NRA                 FAK-KCQPFRKLALE-DQVRMLQ
Mg2DBD-NRA                 FAK-KCQPFRKLALE-DQVRMLQ
Dp2DBD-NRA                 FAK-LVPGFKGLCLN-DQVKLIQ
Me2DBD-NRA                 FAK-KCQPFRKLALE-DQVRMLQ
Mm2DBD-NRA                 FAK-LVPGFKTLSLN-DQVKLIQ
Po2DBD-NRA                 YAK-KVPGFRDIKLD-DQVRLLS
Em2DBD-NRA                 FAK-KVPGFRDLKLD-DQVKLLR
Hruf2DBD-NRA               FSK-KLPNFQSLSLQ-DQVSLIQ
My2DBD-NRA                 FSK-KVPGFRAISLD-DQIKLIQ
Pca2DBD-NRA                FAK-RVPGFRKLPLD-DQVLLVQ
Ech2DBD-NRA                YAK-KTSGFRDLKLD-DQVKLLS
Bg2DBD-NRA                 FAK-KVPGFRLLKIE-DQVLLLR
Ga2DBD-NRA                 FAK-KVPGFKSLVIE-DQILLMQ
Hrub2DBD-NRA               FSK-KLPNFQSLLLE-DQVSLIQ
Acas2DBD-NRA               FAK-KICGFRSLDIN-DQVLLLR
Bt2DBD-NRA                 FAK-KVPGFRQLKIE-DQVLLLR
Bat2DBD-NRA                FAK-KVPDFRKLPID-DQVALVQ
Ng2DBD-NRA                 FAK-KIPGFR-KLSISDQITLVQ
La2DBD-NRA2                FGK-KIPGFR-AIPMEDQICLVR
Bc2DBD-NRA4a               FIS-NIPYITKFSDI-DKNIIYV
Bc2BD-NRA4b                FIK-QTKFLRKFNEN-DQNIILI
Bp2BD-NRA4                 -QE-HELVLERFSIE-DQKILLV
Ar2DBD-NRA2a               FVD-GIPNFNSLNVE-DKTSLVY
Ar2DBD-NRA2b               FIE-GIPNFNSLNVE-DKTSLVY
Rs2s2DBD-NRA2b             FID-RIPNFNSIHFI-EKTSLTH
```

Rso2DBD-NRA2b	FID-KIPNFNSISFI-EKTSLIH
Rs1s2DBD-NRA1a	FID-KIPNFNSINFI-EKSSLIH
Rm2DBD-NRA2b	FID-RIPNFNSINFI-EKTSIIH
Rso2DBD-NRA2a	FAL-RIPDFCSLNSE-DKNLLIH
Rs1s2DBD-NRA2d	FAL-RIPDFCTLNSE-DKSLLIH
Rm2DBD-NRA2a	FAL-RIPEFCSLTAD-DKNLLIH
Ar2DBD-NRA2a	FAL-TIPEFSSLNSD-DRTLLIH
As2DBD-NRA2	FTL-NITDFCILNSD-DKNLLID
Dc2DBD-NRA2b	FST-EIPGFSTFAHS-DHLLLIR
Dc2DBD-NRA2a	FAT-EIPGFSTFIHD-DNLLLIR
Rso2DBD-NRA3a	FCQRTIPGFTNMS---NKYEIIS
Rs2s2DBD-NRA3a	FCQSTIPDFTNMP---DKYEIIS
Rsp12DBD3	FCQKTIPGFTNMS---QKYEIIS
Rsp2_2D6	FCQRTIPGFINMS---QKYEIIS
Rm2DBD-NRA3b	FCQSIIPGFSNTS---DKYEIIS
Rm2DBD-NRA3a	FCQKIIPDFTSMP---DQWEIIS
Ar2DBD-NRA3a	FCQKTIPGFDSMY---EKYDVIS
As2DBD-NRA3a	FCQKTIPGFDNMY---EKYEVIS
Ar2DBD-NRA3b	FCQKTIPGFDNMY---EKYDVVS
As2DBD-NRA3b	FCQKTIPGFDNIY---EKYEIIS
Dc2DBD-NRA3a	FCQKTIYGFSELS---DPYEVLS
Dc2DBD-NRA3b	FCQKTITNFNNLS---DPYEIIS
Bc2DBD-NRA1b	FLS-DFKEFMNFNQM-DREELIK
Ar2DBD-NRA1b	FAT-RIPGFMNLQNVTDQVQLIK
As2DBD-NRA1b	FAT-RIPGFMNLQNVTDQVQLVK
Rso2DBD-NRA1	FAT-RIPGFMNFHNVNDQIQLIK
Ar2DBD-NRA1a	FAT-RIPNFMNFHTVTDQIQLIK
**2DBD-NRB**	
Pmi2DBD-NRB	FAK-NIPGFREIPVS-DQMNLIK
Aru2DBD-NRB	FAK-NIPGFCHISVS-DQMSLIK
Ema2DBD-NRB	FCR-NIPGYFKIDIQ-DRISLGK
Ap2DBD-NRB	FAK-NIPGFRHISVN-DQMNLIK
Aca2DBD-NRB	FCR-NIPGYFKLDIQ-DRICLGK
Lv2DBD-NRB	YIK-KIPGFASIDMN-DRINLIK
Cg2DBD-NRB	FVK-TIPGFDSINSE-DAAELCV
Spur2DBD-NRB	YIK-KIPGFPSIHMN-DRLYLIK
Cv2DBD-NRB	FVR-TIPGFESINQE-DAAELCI
Hruf2DBD-NRB2	FMS-KVPGFKDLNPE-DRHFVVQ
Mm2DBD-NRB1	FAS-QIPGFSNLHPE-DKAFLCK
Pca2DBD-NRB3	CLA-ALPGCEDLTSE-DRALLSQ
Aca2DBD-NRB2	FVL-KLPGMDKIHPK-DRVKLGN
Hrub2DBD-NRB2	FMS-KVPGFKDLNPE-DRNFVVQ
Me2DBD-NRB1	FVM-QIPGFMQLDHD-DKQDLCQ
Mc2DBD-NRB1	FCM-QIPGFMQLDHE-DKQDLCQ
Ech2DBD-NRB3	FCR-NIPGYFKIDIQ-DRISLGK
**2DBD-NRC**	
Cb2DBD-NRC	FLR-GIPRMDEIEME-ERLMLLK
Cr2DBD-NRC2	FVR-NLPHFNLFGMN-DKIILFK
Cr2DBD-NRC1	FVN-RLPSIDSFEIS-DKAILFK
Cr2DBD-NRC3	FVN-HLPFIKTFEIN-DKVILFK
Cr2DBD-NRC4	FAN-HLPFIKTFEIS-DKTILFK
**NRs from Subfamily 1**	
hTRa (NP_955366.1)	FAK-KLPMFSELPCE-DQIILLK
mTRa (CAA30577.1)	FAK-KLPMFSELPCE-DQIILLK
hTRb (NP_000452.2)	FAK-KLPMFCELPCE-DQIILLK
mTRb (NP_033406.1)	FAK-KLPMFCELPCE-DQIILLK
mRARa (CAA71177.1)	FAK-QLPGFTTLTIA-DQITLLK
hRARa (NP_000955.1)	FAK-QLPGFTTLTIA-DQITLLK
hRARb (NP_000956.2)	FAK-RLPGFTGLTIA-DQITLLK
hRARg (NP_000957.1)	FAK-RLPGFTGLSIA-DQITLLK
mRARg (NP_001398643.1)	FAK-RLPGFTGLSIA-DQITLLK
hRORa (NP_599023.1)	FAK-RIDGFMELCQN-DQIVLLK
hRORb (CAA69929.1)	FAK-RITGFMELCQN-DQILLLK
mRORg (NP_035411.2)	FAK-RLSGFMELCQN-DQIILLK
hRORg (AAA64751.1)	FAK-RLSGFMELCQN-DQIVLLK
dHR3 (NP_001246236.1)	FAK-LIPGFMRLSQD-DQILLLK
dECR (NP_001163061.1)	FAK-GLPAFTKIPQE-DQITLLK
mLXRb(NP_001272446.1)	FAK-QVPGFLQLGRE-DQIALLK
hLXRb (BAH02288.1)	FAK-QVPGFLQLGRE-DQIALLK
hLXRa (NP_001171201.1)	FAK-QLPGFLQLSRE-DQIALLK
hFXRa (NP_005114.1)	FTK-KLPGFQTLDHE-DQIALLK
mFXRa (NP_033134.2)	FTK-KLPGFQTLDHE-DQIALLK
hVDR (XP_024304946.1)	FAK-MIPGFRDLTSE-DQIVLLK
hPXR (BAH02292.1)	FAK-VISYFRDLPIE-DQISLLK
mPXR (NP_035066.1)	FAK-VISYFRDLPIE-DQISLLK
hCAR (NP_001070948.1)	FTK-DLPVFRSLPIE-DQISLLK
mCAR (AAC53349.1)	FTK-DLPLFRSLTME-DQISLLK
