# Supplementary material for: Updated knowledge and a proposed nomenclature for nuclear receptors with two DNA binding domains (2DBD-NRs)
Source: PLoS One. 2023 Sep 12;18(9):e0286107. doi: 10.1371/journal.pone.0286107 (PMC10497141; doi:10.1371/journal.pone.0286107)
Supplement: S4 File — (NCBI accession number of 2DBD-NRs see Table 6, accession number of other NR is in the bracket after each NR name). (PDF) [file pone.0286107.s004.pdf]

Supplemental material 4. AF2 sequence (yellow highlighted) for sequence logo

(NCBI accession number of 2DBD-NRs see table 6, accession number of other NR is in the bracket after each NR name)

**2DBD-NRA**	
Rso2DBD-NRA1	IEFPAFFSRVYLNEQDI
Ar2DBD-NRA1a	VELPAFFLRVYLNEQD-
Ar2DBD-NRA1b	ITYPAFFSRVYLNEHDT
As2DBD-NRA1b	IEYPAFFSRVYLNEHNI
Rso2DBD-NRA3a	RNLPSFFYRIFLP----
Rsp2DBD-NRA3a	GNLPSFFYRIFLP----
Rs2s2DBD-NRA3a	PNLPSFFYSIFLP----
Rm2DBD-NRA3a	QNLPSFFYSIFLP----
Rs1s2DBD-NRA3b	RNLPSFFYRIFVPKQIG
Rs2s2DBD-NRA3c	RNLPSFFYRIFVP----
Rm2DBD-NRA3b	RNLPSFFYRIYVP----
Rso2DBD-NRA3b	RNLPSFFYRIYVP----
Ar2DBD-NRA3b	QNVPSYFYRIYLP----
Ar2DBD-NRA3a	RNLPSFFYRIYVP----
As2DBD-NRA3a	RNLPSFFYRIYVP----
As2DBD-NRA3b	PNLPSFFYPIYLP----
Dc2DBD-NRA3a	RNLPSFFYRIYVPN---
Dc2DBD-NRA3b	KNLPFYFYRIYVP----
Sb2DBD-NRA2	LRFPELYVEMFQLADSA
Sm2DBD-NRA2	LRFPELYVEMFQLADSA
Sj2DBD-NRA2	LRFPELYVEMFQLADSA
Of2DBD-NRA2	LHFPELYIEMFQLADSA
Cs2DBD-NRA2	LHFPELYIEMFQLADSA
Ov2DBD-NRA2	LHFPELYIEMFQLADSA
Pw2DBD-NRA2	LHFPELYVEMFQLADSA
Phe2DBD-NRA2	LHFPELYVEMFQLADSA
Psm2DBD-NRA2	LHFPELYVEMFQLADSA
Fh2DBD-NRA2	LYFPELYVEMFRLADDA
Eg2DBD-NRA2	LRYPDLYVEMYEIGESA
Eg2DBD-NRA2	LRYPDLYVEMYEIGESA
Em2DBD-NRA2	LRYPDLYVEMYEIGESA
Ta2DBD-NRA2	LRYPDLYVEMYEIGESA
Hm2DBD-NRA2	LRYPDLYSEMYELPR
Hd2DBD-NRA2	LRYPDLYAEMYEINETS
Mc2DBD-NRA2	LGYPDLYVEMYQLTDPP
Se2DBD-NRA2	LHFPELFVEMFQLSVTP
Sj2DBD-NRA2	LEFPQLYLEMFQLTDEE
Sm2DBD-NRA3	LEFPQLYLEMFQLTDEE
Ph2DBD-NRA3	LVFPELYTEMFQLTEEA
Pw2DBD-NRA3	LVFPELYTEMFQLTEEA
Of2DBD-NRA3	LDFPELYTEMFQLTEAE
Ov2DBD-NRA3	LDFPELYTEMFQLTEAE
Em2DBD-NRA3	LVFPELYVEMFAIGADK
Eg2DBD-NRA3	LVFPELYVEMFAIGADK
Ts2DBD-NRA3	LVFPELYVEMFAIGADK
Hs2DBD-NRA3	LIFPELYLEMFAIGAGE
Hm2DBD-NRA3	LIFPELYLEMFAIGAGE
Ht2DBD-NRA3	LVFPELYVEMFAIGTDK
Spr2DBD-NRA3	LVFPALFTEMFALDEPI
Sj2DBD-NRA3	QVFPDFYMQLFQLDD--
Sma2DBD-NRA3	LPFSDLYIQLFQLNE--
Sh2DBD-NRA3	LPFSDLYIQLFQLNE--
Sm2DBD-NRA1	LPFSNLYIQLFQLNE--
Fb2DBD-NRA1	LTFPDLYVQMFRLDE--
Fh2DBD-NRA1	LTFPDLYVQMFRLDE--
Pw2DBD-NRA1	LVFPDLYVQMFQLND--
Pw2DBD-NRA1	LVFPDLYVQMFQLND--
Cs2DBD-NRA1	LKFPDLYVQMFEL-D--
Of2DBD-NRA1	LKFPDLYVQMFEL-D--
Em2DBD-NRA1	LHFPDLFVQMFQLED--
Eg2DBD-NRA1	LHFPDLFVQMFQLED--
Ts2DBD-NRA1	LHFPDLFVQMFQLGS--
Mc2DBD-NRA1	LHFPDLFVQMFQLDV--
Hm2DBD-NRA1	LHFLDLFVQMFQLDTG-
Rs2DBD-NRA1	LHFLDLFVQMFQLDTG-
Hd2DBD-NRA1	LHFPDLFVQMFQLDTG-
Sp2DBD-NRA1	LHFPDLYIQMFRLDDST
Sme2DBD-NRA3b	LKFPNLYTQMFLID
Bg2DBD-NRA	LHIPQLYAEMYNSVTTG
Ac2DBD-NRA	LAVPQLYAEMYAS
Pc2DBD-NRA	LEVCALYKEMFF-
My2DBD-NRA	LTLPQMYKEMFGEKVLE
Mm2DBD-NRA	IKVPQLFHELIIESIKE
Ga2DBD-NRA	LQVNILFKEIFAV
Cg2DBD-NRA	LEISPLMREVYHLNPK
Cv2DBD-NRA	LEISPLMREVYHLNPK
Of2DBD-NRA	LPLPQLFTEMFLND
Bf2DBD-NRA	LKIPQLFSELHKV
Bb2DBD-NRA	LKIPQLFSELHKV
**2DBD-NRB**	
Ap2DBD-NRB	VTLPALFAEINL
Pmi2DBD-NRB	VTLPALFAEINL
Aru2DBD-NRB	AVLPALFAEINL
Lv2DBD-NRB	FSMPALFAEINLSS
Spur2DBD-NRB	VSMPALFAEINLS
Cg2D2-NRB	QEMHQLFDKLDLENPMS
Cv2D2-NRB	QEVHQLFDKLDLENPLS
**2DBD-NRC**	
Cr2DBD-NRC4	LFKKNCFLMFLLRHVL
Cr2DBD-NRC1	LFKRSSFLMFILRNIT
Cr2DBD-NRC2	LFKRSSFLMFILRNIT
Cr2DBD-NRC3	LFKRSSFLMFVLRNIT
**NRs from Subfamily 1**	
hTRa (NP_955366.1)	ELFPPLFLEVFEDQEV
hTRb (NP_000452.2)	ELLPPLFLEVFED
xTRb (NP_001090533.1)	ELFPPLFLEVFED
mTRb (NP_033406.1)	ELFPPLFLEVFED
hRARa (NP_000955.1)	GSMPPLIQEMLENSEGL
mRARb (NP_035373.1)	GSMPPLIQEMLENSEGH
hRARb (NP_000956.2)	GSMPPLIQEMMENSEGH
hRARg (NP_000957.1)	GPMPPLIREMLENPEMF

mRARg (NP_001398643.1)	GPMPPLIREMLENPEMF
hPPARa (AAA36468.1)	-ALHPLLQEIYRDMY
mPPARa (CAA40856.1)	-ALHPLLQEIYRDMY
hPPARd (NP_001165289.1)	-SLHPLLQEIYKDMY
mPPARd (AAA19972.1)	-LLHPLLQEIYKDMY
mPPARg (AAA62110.1)	-SLHPLFQEIYKDLY
hPPARg (BAA18949.1)	-SLHPLLQEIYKDLY
dE78 (AAF69494.1)	--LPPLFAEIFDIPKADDEL
hRORa (NP_599023.1)	LHFPPLYKELFTSEFEPAMQIDG
mmRORa (AAB46801)	LHFPPLYKELFTSEFEPAMQIDG
mcRORa (NP_001276845.1)	LHFPPLYKELFTSEFEPAMQIDG
hRORb (CAA69929.1)	TLFPPLYKELFNPDCATACK
mRORg (NP_035411.2)	AAFPPLYKELFSTDVESPEGLSK
hRORg (AAA64751.1)	AAFPPLYKELFSTETESPVGCPS
dHR3 (NP_001246236.1)	VVFPALYKELFSIDSQQDLT
bECR (NP_001166846.1)	--LPPFLEEIWDVAEVATTH--P
dECR (NP_001163061.1)	--LPKFLEEIWDVHAIPPSVQSH
mLXRb (NP_001272446.1)	--LPPLLSEIWDVHE
hLXRb (BAH02288.1)	--LPPLLSEIWDVHE
mLXRa (NP_001171201.1)	--LPPLLSEIWDVHE
hLXRA (AAA85856.1)	--LPPLLSEIWDVHE
hFXRa (NP_005114.1)	--FTPLLCEIWDVQ
mFXRa (NP_033134.2)	--FTPLLCEIWDVQ
hVDR (XP_024304946.1)	MKLTPLVLEVFGNEIS
mVDR (BAA06737.1)	MKLTPLVLEVFGNEIS
hPXR (BAH02292.1)	-FATPLMQELFGITGS
mPXR (NP_035066.1)	-FATPLMQELFSSTDG
hCAR (NP_001070948.1)	-AMMPLLQEICS
mCAR (AAC53349.1)	-AMTPLLGEICS
