# Supplementary material for: Updated knowledge and a proposed nomenclature for nuclear receptors with two DNA binding domains (2DBD-NRs)
Source: PLoS One. 2023 Sep 12;18(9):e0286107. doi: 10.1371/journal.pone.0286107 (PMC10497141; doi:10.1371/journal.pone.0286107)
Supplement: S5 File — LBD sequence alignment of all 2DBD-NRs to identify the conserved amino acids in class I or class II NRs according to [50] (Signature of the oligomeric behavior of nuclear receptors at the sequence and structural level. Blocks indicate the variable inserts are deleted according to [50]). (PDF) [file pone.0286107.s005.pdf]

**Supplemental material 5. LBD sequence alignment to identify the conserved amino acids in class I or class II NRs**

A. LBD sequence alignment of all 2DBD-NRs to identify the conserved amino acids in class I or class II NRs according to: Brelivet, Y., et al. *2004 (Signature of the oligomeric behaviour of nuclear receptors at the sequence and structural level. EMBO Rep, 5(4): p. 423-9)*. Blocks indicate the variable inserts are deleted according to Brelivet et al.

```
                                                 40                   60                   80                   100
                                                   ED42     E50          R62                        HRK90           R105
Class II (hVDR)      IQKVIGFAKMIPGFRDLTSEDQIVLLKSSAIEVIML HEEEHVLLMAICIV DRPGVQDAALIEAIQDRLSNTLQTYIRCRHP LLYAKMIQKLADLRSL
Class I  (hRXRa)     LFTLVEWAKRIPHFSELPLDDQVILLRAGWNELLIA DKTELGCLRAIVLF DSKGLSNPAEVEALREKVYASLEAYCKHKYP GRFAKLLLRLPALRSI
                                                 W40        E50  KR55                                   RK93    P102 R105

2DBD-NRA
Platyhelminths
Pw2DBD-NRA2          AQQIVQFAKLVPGFNHLGIAARSNLVRGAMYPVVLL SLPELSLSSAVEIL YQLDEPTRSNTKELFILAHHSLRACMTREFD HRWAQLTALSKMIQAM
Psm 2DBD-NRA2        AQQIVQFAKLVPGFNHLGIAARSNLVRGAMYPVVLL SLPELSLSSAVEIL YQLDEPTRSNTKELFILAHHSLRACMTREAD HRWAQLTALSKMIQAM
Cs2DBD-NRA2          AQQIVQFAKLVPGFNQLGITARSNLVRGAMYPVMLL SLPELSLSAAVEIL HQLEEPTRTSTRELFVLAHHSLRVCMTREQE HRWAQLVALTKMLQAM
Of2DBD-NRA2          AQQIVQFAKLVPGFNQLGITARSNLVRGAMYPVMLL SLPELSLSAAVEIL HQLEEPTRTSTRELFVLAHHSLRVCMTREQE HRWAQLVALTKMLQAM
Ov2DBD-NRA2          AQQIVQFAKLVPGFNQLGITARSNLVRGAMYPVMLL SLPELSLSAAVEIL HQLEEPTRTSTRELFILAHHSLRVCMTREQE HRWAQLVALTKMLQAM
Fh2DBD-NRA2          AQQIVQFAKLVPGFNHLGITARSNLVRGAMYPVVLL SLPELSLLCAVEIV YQLDEPTRSSTKELYILAHHSLRMYMTNDSD HRWAQLVAMSKMIQAM
Sm2DBD-NRA2          AQQVVQFAKLVPGFNQLGITARSNLVREAMYSVLLL SLPELSLSCAAEIL YCLEEPTAKSTAELFVLAHHSLLNCMAKVAS QRRTQLFALRKMIRVM
Sj2DBD-NRA2          AQQIVQFAKLVPGFNQLGITARSNLVREAMYSVLLL SLPELSLSCAAEIL YCLEEPTASSTAELYVLAHHSLLNCMTKLVS QRRTQLFALRKMIRVM
Hd2DBD-NRA2          SQQVVQFAKLIPGFNQMSLSARGHLVRASLYSEVLL THTEFVYACAIGIL YYLANPAYTDARKLLLLARHSLLATMRSQEL HKWNRLEALSEMLSSM
Hm2DBD-NRA2          SQQVVQFAKLIPGFNQMSLSARGHLVRASLYSEVLL THTEFVYACAIGIL YFLADPAYTDARKLLLLARHSLLATMRSQEL HKWNRLEALNEMLSSM
Eg2DBD-NRA2          SQQVVQFAKLIPGFNQMSLSARGHLVRASLYSEVLL THTEFVYVCAIGIL YILANPAYMDARKLLLLARHSLLATMRSQEL HKWSRLEAMSEMLSSM
Em2DBD-NRA2          SQQVVQFAKLIPGFNQMSLSARGHLVRASLYSEVLL THTEFVYVCAIGIL YYLANPAYTDARKLLLLARHSLLATMRSQEL HKWSRLEAMSEMLSSM
Se2DBD-NRA2          SQQIVQFAKLIPGFNQLELTARGALVREGMYAAMLL TLPEFTLVCAIEIL FALSGASCTHARRFFLLAKHSLLATMRNREP KRCAQLESFSQVLHDL
Sma2DBD-NRA2         AQQVVQFAKLVPGFNQLGITARSNLVREAMYSVLLL SLPELSLSCAAEIL YCLEEPTAKSTAELFVLAHHSLLNCMAKVAS QRRTQLFALRKMIRVM
Sh2DBD-NRA1          IYEIIQFAQSIPSFQDLSEYDMKILIQQSIYPIILI NETEIGLLCCVELF DGKCLNEPIKVYETYQTILQLLKEYETNQFN KRFYKIMSIKHNLDRM
Sb2DBD-NRA1          IYEIIQFAQSIPSFQELSEYDMKILIQQSIYPIILI NETEIGLLCCVELF DGKCLNEPIKVYETYQTILQLLKEYETNQFN KRFYKIMSIKHNLDRM
Sma2DBD-NRA1         IYEIIQFAQSIPSFQELSQYDMKILIQQNIYPIILI NETEIGLLCCVELF DGKCLNEPIKVNETYQTILQLLKEYETNQFN KRFYKIMSIKHNLDRM
Sm2DBD-NRA1          IYEIIQFAQSIPNFQELSEFDMKILIQQSIYPIILI NETEIGLLCCVELF DGKYLNEPIKINEAYQTILQLLKEYETIQFN KRFYKIISIKHNLDRM
Sj2DBD-NRA1          IYEITQFAQTISSCQELSEFDMKILIQQSVYPIILI NETEIGLLCCIELF GGGFLNEPIKVNETYQTILQLLKEYETNRFN LRYYEIMSIKNNLDKM
Ov2DBD-NRA1          AHQVIQFARAVPGFRELPSVVMKKLVQEGMYPITVI DETETALLCCIQLF GGERFGETSKVETMYQRTVSALRTYEETRQM VRTQNILAVTSLLTDM
Of2DBD-NRA1          AHQVIQFARAVPGFRELPSVVMKKLVQEGMYPITVI DETETALLCCIQLF GGERFGETSKVETMYQRTVSAIRTYEETRQM VRTQNILAVTSLLTDM
Cs2DBD-NRA1          AHQVIQFARAVPGFRDLPSVVMKKLVQEGMYPITMI DETETALLCCIQLF GGERFGETSKVETMYQRTVSALRTYEETRQM VRTQNILAVTSLLTDM
Phe2DBD-NRA1         AHQVIHFARAVPGFRDLSRLDMKTLVQESMYPIVLI DDTETALYCTVQLF GGEHFDEPAKIETMYKQAVAALRKYEQINFG DRAQSILTVTSILAEM
Psm2DBD-NRA1         AHQVIHFARAVPGFRDLSRLDMKTLVQESMYPIVLI DDTETALYCTVELF GGEHFDEPVKIETMYKQAVAALRKYEQINFG DRAQSILTVTSILAEM
Pw2DBD-NRA1          AHQVIHFARAVPGFRDLSRLDMKTLVQESMYPIVLI DETETALFCTVELF GGEHFDEPARIETMYKQAVAALRKYEQINFG DRAQSILTVTSILAEM
Spr2DBD-NRA1         AHQVIRFARAIPGFRDLPRSDTKVLVQESMYPIVLI DENEIALLCCIVFL FNRNLIESDRIYEMRAHAVSALRTYITVQYS ERFYSLMEVLPILTNM
Eg2DBD-NRA1          AHQIIRFARAIPGFCDLPRADTKFLLQASMYPIVLV DNTEAALLCSIIFL SNQKLLEAEKIYEIYNHAASALQQYITVRYS ERFTQLIKLLPSLSSM
Em2DBD-NRA1          AHQIIRFARAIPGFCDLPRADTKFLLQASMYPIVLV DNTEAALLCSIIFL SNQKLLEAEKIYEIYNHAASALQQYITVRYS ERFTQLIKLLPSLSSM
Ta2DBD-NRA1          AHQIIRFARAIPGFRDLPRADTKFLVQASMYPIVLV DNTEAALFCSIIFL SNQKLLEAGKIYEIYNHAASALQQYITVRYS ERFTHLIKLLPSLSSM
Ht2DBD-NRA1          AHQIIRFARAIPGFRDLPREDTKFLVQASMYPIVLI DNTEAALLCSIIFL SNQKLFEAGKIYEIYNHAASALQQYITVRYS ERFTHLIKLLPSLSSM
Hm2DBD-NRA1          AHQVIRFARAIPGFRDLPRADTKFLVQASMYPIVLV DNTEAALFCSIIFL SNQKLQEAEKIYEIYNHAALALQQYITVRYS ERFTRLIKLLPSLSSM
Hd2DBD-NRA1          AHQVIRFARAIPGFRDLPRADTKFLVQASMYPIVLV DNTEAALFCSIIFL SNQKLQEAEKIYEIYNHAALALQQYITVRYS ERFTRLIKLLPSLSSM
Hn2DBD-NRA1          AHQVIRFARAIPGFRDLPRADTKFLVQASMYPIVLV DNTEAALFCSIIFL SNQKLQEAEKIYEIYNHAALALQQYITVRYS ERFTRLIKLLPSLSSM
Fg2DBD-NRA1          THQIIQFARAVPGFRDLSRLDMKTLVQSGTYPILLL DQSEVGLLFCLALM DAQELEEPNKVHAIYQQALLALEKHEQPRTN QRVRDLLAVIPVLIEM
Fbu2DBD-NRA1         EHQIIQFARAVPGFRDLSRMDMKTLVQSGTYPILLL DQRETGLLCCLTLL DAEQFDEPNKVQAIYQQALLALEKHEQTRKD QRVRDILAVIPVLMEM
Eca2DBD-NRA1         AHQITRFAREVPGFRDLSRVDMITLVQSGTFPIILL DESEMGLLCCLVLL DDQQFEDPNRVQTTYQHALMVLEKYEQARAN QRIRDLLAVIPILDEM
Fh2DBD-NRA1          THQIIQFARAVPGFRDLSRLDMKTLVQSGTYPILLL DQSEVGLLFCLALM DAQELEEPNKVHAIYQQALLALEKHEQPRTN QRVRDLLAVIPVLIEM
Phe2DBD-NRA3         SRFIVHFVKLIPGFNQLVLDDRRQLVRGAMYPLMLL DNVELTLICAQEVF NYHGLIDPPATEHLYYLAGRVLVDHIVTSGQ ERSNRLRDLSPMLEQL
Pw2DBD-NRA3          SRFIVHFVKLIPGFNQLVLDDRRQLVRGAMYPLMLL DNVELTLVCAQEVF NYHGLIDPPATEHLYYLAGRVLVDHIVTSGQ ERSNRLRDLSPLLEQL
Of2DBD-NRA3          ARFVVHFVKLIPGFDQLELGDKRQLVRGAMYPLMLL DNVELALVCAQEVF HYHGLIDPPATQHLYNLASRALTDYIITTGQ ERSTLLRHLSSLLEEL
Fh2DBD-NRA3          SRFIVHFVKLIPGFNQLELSDKRQLVRKAMYPLMLL DNIELVLVCAQEVF SQPGLIDSASADHLFSLASRALTNHIIDSGQ ERSSMLRLLSPLLEEL
Spr2DBD-NRA3         SKMVVNFTKIIPGINRLELNDKRQLVRSAMYPLMLV DDIEFTLICTQELL SQNDLSDPTSCQHLLLLSMQVLVNHEQSIAK QRLTAISQLLPMLNHF
Se2DBD-NRA3          SKMVVNFTKIIPGINRLELNDKRQLVRSAMYPLMLV DDIEFTLICTQELL SQNDLSDPTSCQHLLLLSMQVLVNHEQSIAK QRLTAISQLLPMLNHF
Eg2DBD-NRA3          TRMVVDFSKLIAGFNRLGINDRRQLIRAAMYPIMLI DDIESTIICIQELL HKNELEDPASCEHLFLLSMQALVNHEHQKSK ERLTAFTQLLPILNQL
Ta2DBD-NRA3          TRMVVDFSKLIAGFNRLGINDRRQLIRAAMYPIMLI DDIESTIICIQELL HKNELEDPASCEHLFLLSMQALVNHEHLKSK ERLTTFTQLLPMLNQL
Hm2DBD-NRA3          TRMVVDFSKLIAGFNGLGINDRRQLIRAAMYPIMLI DDIECTIICIQELL HKNELEDPASCEHLFLLSMQALVYHEQCKSK ERLTTFTQLLPMLNQL
Sm2DBD-NRA3          SRFIVQFVKYIPGFCYLKISDQRQLVRSAMYPIMLL DNIELTLMCAQEVF DRQGLDDPVTPAYLFNLVGLALTEHIISVGY GRYAALSLISPMLEEL
Sh2DBD-NRA3          SRFIVQFVKYIPGFCYLKISDQRQLVRSAMYPIMLL DNIELTLMCAQEVF DRQGLDDPVTPAYLFNLVGQALTEHIISVGY ERCAALSLISPMLEEL
Sj2DBD-NRA3          SRFIVQFVKYIPGFCYLKISDQRQLVRSAMYPIMLL DNIELTLICAQEVF DRQSLDDPVTPAYLHSLVRQVLADHIVSVGY ERCSSLSLISPMLEEL

Mollusca
Ga2DBD-NRA           AKCTITFAKKVPGFKSLVIEDQILLMQLATYPISLL DPTELSIMCALILF ECEILKENWKVKEWHDYYIECLQEYTMSAYT TRFGALLLRLGELTMN
Aca2DBD-NRA          AKCIVRFAKKICGFRSLDINDQVLLLRMATYSLVLL TDMEYAYLSCLLLL ELPDLAKAEEVKELRVRFMGSFQDYEIDNFP LRFGEMMLRLTEYYQF
Cu2DBD-NRA           SQRIVPFAKSRC-FRGLKIDDQVLLLRVATYSLVIL TEVEYAYMSCILLL EYPGLENTVMTQKLKERYVNAFLDYEIEHFP LRFAEMLLHLSEFSQL
Ac2DBD-NRA           AKCIVRFAKKICGFRSLDINDQVLLLRMATYSLVLL TDMEYAYLSCLLLL ELPDLAKAEEVKELRVRFMGSFQDYEIDNFP LRFGEMMLRLTEYYQF
Ech2DBD-NRA          AKIMVRYAKKTSGFRDLKLDDQVKLLSGATYNLVLL TEKEYAYMSSMILL ECEGIEEIHKVRELKDIFLSAFQHYESENFP LRFGQMLLRLSEFSQF
Cg2DBD-NRA           SGELVAALKMFPVFKILELDDRITLVQDSIYSMAIL DDIELTCLTGLLCF DACNLKKRAKVEESQKYIMSFLEMYEEKKYN LRFGMLVLRIGDLMGL
Cv2DBD-NRA           SGELVAALKMFPVFKILELDDRITLVQDSIYSMAIL DDIELTCLTGLLCF DACNLKKRAKVEESQKYIMSFLEMYEEKKYN LRFGMLVLRIGDLMGL
My2DBD-NRA           ARKLIKFSKKVPGFRAISLDDQIKLIQASIYPIVVL SSMEYTFLSILLLL DVENLEDSERVRKLLLDTTFALQYHEETTYT TRFGMLLVRLAELHFI
Pm2DBD-NRA           ARTLIKFSKKVPGFRALSLDDQIKLVQGSIYPIVVL STMEYTFLSILLLL DVEDLDDSESVRKLQMDTTFALQYHEESNYP TRFGMLLVRLAELHFI
Mc2DBD-NRA           SSVIIKFAKKCQPFRKLALEDQVRMLQQALYPISIL DDDEFTILSTLVLL DVPGLIDKEKVRQLQAELLDFLEYYLSITYP SRYGLVLVRLSELNFY
Mg2DBD-NRA           SSVIIKFAKKCQPFRKLALEDQVRMLQQALYPISIL DDDEFTILSTLVLL DVPGLIDKEKVRQLQAELLDFLEYYLSITYP SRYGLVLVRLSELNFY
Me2DBD-NRA           SSVIIKFAKKCQPFRKLALEDQVRMLQQALYP---- DD-EFTILSTLVLL DVPGLIDKEKVRQLQAELLDFLEYYLSITYP SRYGLVLVRLSELNFY
Mm2DBD-NRA           TYITIRFAKLVPGFKTLSLNDQVKLIQSSIYPIELL TDEEFAILSALLLF EANGLENPKAVEQLQNQISAALQAYEEREFP TRYGILLVRVAELVQC
Dp2DBD-NRA           TYITIRFAKLVPGFKGLCLNDQVKLIQSSIYPIELL TDEEFAMLSALLLF EIHGLEDPKAVEAMQTEVSAALQYYEEVTFP TRYGILLVRVAELVQC
Pc2DBD-NRA           AQCIIRFAKRVPGFRKLPLDDQVLLVQLAIYPIVIL DNVEYTLLSCMLLV ECPGIVEKEKALELQTKLLKVFQLYE-ER-- CRFGELMLLISELSLA
Pca2DBD-NRA          AQCIIRFAKRVPGFRKLPLDDQVLLVQLAIYPIVIL DNVEYTLLSCMLLV ECPGIVEKEKALELQTKLLKVFQLYE-ER-- CRFGELMLLISELSLA
Bg2DBD-NRA           AKCIVRFAKKVPGFRLLKIEDQVLLLRTATYGLVIL TDLEYAYLSCMLLL EYQGLIDTKKVKELKEKYMEAFHEYEFHNFP LRFGEMLLRLSEFSQF
Bt2DBD-NRA           AKCIVRFAKKVPGFRQLKIEDQVLLLRMATYSLVIL SDLEYAYLSCMLLL EHQGLIDTKKVKELKEKYVEAFHNYEIESFP LRFGEMLLRLSEFSHF
Po2DBD-NRA           AKAMVRYAKKVPGFRDIKLDDQVRLLSKATYNLVLL TEIEYAYMSCIMLL ECEGLESVPQVKELKSIILAAFQQHQLENFP LRFGQLLLRLSEFSQF
Dg2DBD-NRA           AQGVLRFAKKVPGFKDIHVEDQIVMVQFATYQIVMI DDIEVAVLCAIVLI ENEFKTGKQIIETVSAIQHGFLRRYCESKFR SRQNDMLSLLTDLKET
Ema2DBD-NRA          AKVLVRFAKKVPGFRDLKLDDQVKLLRSASYSCVLL TEKEYGYMSCMLLV ECEGLDDPDRVRQLKDIIMAAFQQYEFDNFP LRFGQTLLRLSEFSQF
```

```
Annelida
Ls2DBD-NRA          ARSVLKFTKKVPGVRQLPIDTQIMMVQQAMYPIVLL DEIECAFLCGLCLL ------QSDGTYGYQEALVSAMAVYCERKAL ERVGLLLLRLCEMLRC

Lingulata
La2DBD-NRA          VHCLIKFGKKIPGFRAIPMEDQICLVRHTNYIMSLAD DIEYPMLCALILL SDLELKEPGKIDQLSAKVSNAFKAYMTNKYG PRYFSIIQILQDLHQA

Rotifera
Rso2DBD-NRA4        ANRSVKFATRIPGFMNFHNVDQIQLIKSSIHSIIIL DEKEFSLLLSLLII SNIHLNDFLLIDSTQTELTCALCDYMDS-KR GRFYTLMLLLPSLRKL
Rss2DBD-NRA1b       ANRSVKFATRIPGFMNFHNVDQIQLIKSSIHSIIIL DEKEFSLLLSLLII SNIHLNDFLLIDSTQTELTCALCDYMDS-KR GRFYTLMLLLPSLRKL
Rs2DBD-NRA1         ANRCVKFATRIPGFMNFHNVDQIQLIKSSIHSIIIL DEKEFSLLLSLLII SNVHLNDFLLIDSTQTELTCALCDYMDN-KR GRFYTLMLLLPSLRKL
Ar2DBD-NRA1a        ANRAVKFATRIPNFMNFHTVDQIQLIKSSIHSIVIL DEKELSLFLSLLII SNIRLNDFLLIDSTQTELTCALCDYMDC-KR GRFYTLMLLLPSLKKL
Ar2DBD-NRA1b        ANRSVKFATRIPGFMNLQNVDQVQLIKSSIHSLIIL DEKEFTLFLSLLII SNIYLSEFLLIDSTQTELTCALCDYMDC-KR GRFYTLMFLLPTLRKL
As2DBD-NRA1b        ANRTVKFATRIPGFMNLQNVDQVQLVKSSIHSLIIV DEKEFSLLLSLLLV GNTYLNDFLLIDSTQTQLTILLSDYMDC-KR GRFYTVMFLLPTLRKL
Ar2DBD-NRA2b        SICVRNFIEGIPNFNSLNVEDKTSLVYQSIHSVMLM NDKEIALVLILLIT GDYKSTPI---EHLEEEFFSILFDYMTV-QR GKDYFILTIIPFVHRL
Rso2DBD-NRA2b       SNRIRDFIDKIPNFNSISFIEKTSLIHLSNHSVILM DDKEISLMIILLIT GNNKLKEI---ENIEEEFFSILHDYMAV-KR GKDYFILTIIPFLHRI
Rm2DBD-NRA2b        SNRVRAFIDRIPNFNSINFIEKTSIIHLSTHSVILM DDKEIALMLALLIT GNNKSKEI---EYLEEELFTILYDYMAV-KR GKDYFILTIIPFLHRI
As2DBD-NRA2         TARVRDFTLNITDFCILNSDDKNLLIDLSTHSVIVL DDKEIALFIILLIT PNSKFKEM---EKIQEDFFCILYDYMSA-KR GTDYFILTVIPFLHRI
Rs2DBD-NRA2b        SNRVRAFIDRIPNFNSINFIEKTSIIHLSTHSVILM DDKEIALMLTLLIT GNNKSKEI---EYLEEELFTILYDYMAV-KR GKDYFILTIIPFLHRI
Rm2DBD-NRA2a        TARVGDFALRIPEFCSLTADDKNLLIHSSTHSVIVA DDREVALMIVLLIT ANSKLNETEKIEKIQEDFFCALYDYMSA-KR GKDYFILTIIPLIHRI
Rs2DBD-NRA2a        TARVGDFALRIPEFCSLTADDKTLLIHSSTHSVIVA DDREIALMIVLLIA ANSKLNETEKIEKIQEDFFCALYDYMSA-KR GKDYFILTIIPLIHRI
Rss1NR7A2a_         TTRVGDFALRIPDFCTLNSEDKSLLIHISTHSVILL DDKEIALMIVLLIT TNSKLKDIEKIDKLQENYFCALYDYMSA-KR GKDYFILTIIPFIHRI
Rso2DBD-NRA2a       TIRVGDFALRIPDFCSLNSEDKNLLIHSSTHSVILL DDKEIALMIVLLIT ANSKLKEIEKIEKIQEDYFCALYEYMAA-KR GKDYFILTIIPFIHRI
Ar2DBD-NRA2a        TKRVGDFALTIPEFSSLNSDDRTLLIHSSTHSVIIL DDRENALFLALLLV ANSKFIEIDKLEKIQENIFCTLYDYMSA-KR GNDYFILTVIPFIHRI
Bc2DBD-NRA4b        LEQCFYFIKQTKFLRKFNENDQNIILINSIHSLRLL DVNEFAIYSLFLVF SCKNLIGFREKFECNLELCNLLCKYMYLRRN EKLINIAPRFERINLQ
Rso2DBD-NRA3a       AQAIMNFCQN----FTNMS-NKYEIISSSINSVIMI DEKEFSLLLLMIIT NLNVINESNKWSKYQYECIQAFCEYTQARRS NINREFSIEIYDIIFL
Rss2DBD-NRA3a       ARAVMNFCQSIPDFTNMP--DKYEIISSSINSVIMV DEKELSLLLLMIIT NLNVINETNKWPKYQYECLQAFCEYTQARRS NINREFSIEVYDVMFL
Rss2DBD-NRA3b       AQAMMNFCHS----FINMS-NKYDIISSSINSVIMV DEKEFSLLLLMIIT KFNSVNENNRWSKCQYECIETFSEYYQARRS NINRELPIEFYDIIFL
Rm2DBD-NRA3a        AQAVMNFCQKIPDFTSMP--DQWEIISSSINSVIII DEKELSLLLLMLIT NCNLMTENHKWLKLQYECVEALAEYTQARRS IEFCDIIFLVSQLRSL
Rs2DBD-NRA3a        AQAVMNFCQKIPDFTSMP--DQWEIISSSINSVIII DEKELSLLLLMLIT NCNLMTENHKWLKLQYECVEALAEYTQARRS IEFCDIIFLVSQLRSL
Ar2DBD-NRA3a        AQAVMNFCQKIPGFDSMY--EKYDVISSCIHSVIMV DEKEFALLLLMIIT NINVMNEETKWPKCQYECVQAFSEYAQARRS FEFYDIAFLVSQLRTL
As2DBD-NRA3a        AQAVMSFCQKIPGFDNMY--EKYEVISSCINSVIMV DEKELSLLLLMIIT NLNVINEENKWPKCQYECVQAFSEYTQARRL FEFYDIAFLVSQLRTL
As2DBD-NRA3b        SEAVINFCQKIPGFDNIY--EKYEIISSSINSVIMI DEKELSLLLLMIIT NLNVINEENNWPKCQYECVQAFSEYTQARRS LEFYDIIFLVSQLRSL
Rss2DBD-NRA3b       AQALMNFCQKIPGFTNMS--QKYEIISSSINSVIMV DEKEFALFLLMIIT NLNLINEDNKWSKCQYECVQAFSEYTQARRL IEFYDIVFLVSQLRSL
Rss2DBD-NRA3c       AQALMNFCQRIPGFINMS--QKYEIISSSINSVIMV DEREFSLLLLMIIT NLNLINEDNKWSKCQYECVEAFSEYTQAHRL SEFYDIVFLVSQLRSL
Rso2DBD-NRA3b       AQAMMNFCQRIPGFTNMS--NKYEIISSSINSVIMV DEKEFSLLLLMIIT NLSLINDDDKWSKCQYECVHAFSEYIQARRS IEFYDIVFLVSQLRSL
Rm2DBD-NRA3b        AQAIMNFCQSIPGFSNTS--DKYEIISSSINSVIMV DEKEFSLLLLMIIT QLNVINGDNKWSTYQYECVQAFSEYSQARRS IEFYDIAFLVSQIRSL
Dc2DBD-NRA3b        AECLIHFCQKITNFNNLS--DPYEIISSSIHTVIII DENEISLVILICLT NF-LNSDKNSWSKCQFDCVEALSEYMQARRG FEFYDVSFLITQIRSL
Bc2DBD-NRA4a        SDKCYSFISNIPYITKFSDIDKNIIYVNSFHPIRIL DSKELALYSAFLAF SCKGLTGIREKFDCNLELCNALSKYMYLRRN EKLINIAPRFERLNLQ

Chordata
Bb2DBD-NR7A         VHVTTKFAKKIPGFRTCSIDDQISMIQGAAFPINSC NKAEMTLFCGILLM DTAGLKDPKGVEMLQGKLYSILEKYTLAQGV VRFQAMLQAIMALKKL
Bf2DBD-NR7A         VHVTTKFAKKIPGFRTCSIDDQISMIQGAAFPINSC NKAEMTLFCGILLM DTAGLKDPKGVEMLQGKLYSILEKYTLAQGV VRFQAMLQAIMALKKL
Bl2DBD-NR7A         VHVTTKFAKKIPGFRTCSIDDQISMIQGAAFPINSC NKAEMTLFCGILLM DTAGLKDPKSVEMLQGKLYSILEKYTLAQGV VRFQAMLQAIMALKKL

2DBD-NRB
Mollusca
Hur2DBD-NR7B2       VKEELVFMSKVPGFKDLNPEDRNFVVQNMSFANILI DRTEAAMVSALNVI DCIGLKEPEKIEESRAFLISALRAHISYKGV SRMKEIFSLMPTFRLC
Mm2DBD-NRB1         TSAAVKFASQIPGFSNLHPEDKAFLCKSVTFMSTVL DLYEVSVMAALIFL DFLDLAYPEAIEEARAKVIGALKSYEMSKGV SRLTHLFSLIPEIRHV
Cg2DBD-NRB1         ITGFMNFVKTIPGFDSINSEDAAELCVMNSFHIAII TPMEETFLSVITFL DITNLRDPATLESLRQKMIHIFQAQLIAQGV QRIQELFRVMPDCRHC
Me2DBD-NRB1         NKAYVDFVMQIPGFMQLDHDDKQDLCQLASFQAALI SPVELSLTLALSCF DLMFLKDPESVESVRRHIIYLLQQHIAEQGI ERLAAIFNIMPMTRHI
Cg2DBD-NRB1         ITGFMNFVKTIPGFDSINSEDAAELCVMNSFHIAII TPMEETFLSVITFL DITNLRDPATLESLRQKMIHIFQAQLIAQGV QRIQELFRVMPDCRHC
Cv2DBD-NRB1         VSAFMNFVRTIPGFESINQEDAAELCIMNSFHIAII TPLEETFLSIITFL DLLYLRDPATLESLRQKMIHILQAQLYAQGI KRMQELFRAMPDCRHC
Mc2DBD-NRB1         NKAYVDFCMQIPGFMQLDHEDKQDLCQLASFQAALI SPVELSLTLALSCF DLMFLKDPESIESVRRHLIYLLQQHIAEQGI ERLAAIFNIMPMTRHI

Phoronida
Pa2DBD-NRB          NEAQLWFATRIPGFCNLCREDQMILLKYGSFQIGMV DDTEASMLCSLILL DYLELTDTDKIHKLYSMMLKALDAYVYRKIG NRLSDLVSFIPKLRHL

Echinodermata
Ap2DBD-NRB          IIGSVTFAKNIPGFRHISVNDQMNLIKAGTFPCILC DQEEIALFTALIFI DNPGLDDVEFILGLHGKLRQVFKHYCLEKYG LKFISFLPRLYQIELL
Pmi2DBD-NRB         IIGSVNFAKNIPGFREIPVSDQMNLIKLGTFPSILC DEHEIALIMALIFI DPSGLESVDFILNLHGKLRLIFKHYCLEKYG LKFISFLPRLYKIELL
Aru2DBD-NRB         IISSVKFAKNIPGFCHISVSDQMSLIKMGTFPCILS DQHEIALFTTLIFI DPPGLENSDYILKLHKKLRLILKHYCLEKYG YKYLSFLPRLYEIELV
Lv2DBD-NRB          IIASVQYIKKIPGFASIDMNDRINLIKHSVFPIILV DSIEVALLMILVTL DLEGLENKEAVLKLHKDYRTIMQNYCLETHG LIMLQFVPYLSKIDIV
Spur2DBD-NRB        IIASVQYIKKIPGFPSIHMNDRLYLIKHSVFPIILV DSIEVALLMILVVL DLEGLENRQAVLKLHKDYRTIFQKYCHETHG LIMLQFVPYLAKIDLV

2DBD-NRC
Cr2DBD-NRC3         NHQTGLFVNHLPFIKTFEINDKVILFKRSSFLMFVL SHHELSLFTALVFF FTADFKSHKTLELVYHCYKTFLNRMFSKYEN SELDEMTFKLEDLNKL
Cr2DBD-NRC1         NHQTGLFVNRLPSIDSFEISDKAILFKRSSFLMFIL NHQELSLFTAFIFL ITRGFIGDSTLKGVRDYYRTLLYQLMAEREN SDLATMTLKLYDLNKI
Cr2DBD-NRC4         AKQTLQFVRNLPHFNLFGMNDKIILFKKNCFLMFLL KIQEIAVFTALVFF DGS-IKDKERLEVVCEYYKLLLDQEMSERS- EKLVDLMPTLQKMNKD
CbNR7C              IRSLLPFIDELQ--DSFNSHDKAILLKRSAFQIYLL CTDEDIAMFITIVY PGR-LQNPENLSPAQQKFKNLFVKHAESRSV QDLINLWPELERLSEF
```

B. LBD sequence alignment of all 2DBD-NRs. The blue region was deleted for identification of the conserved amino acids in class I or class II NRs according to: Brelivet, Y., et al. 2004. (Signature of the oligomeric behaviour of nuclear receptors at the sequence and structural level. EMBO Rep, 5(4): p. 423-9)

```
2DBD-NRA
Platyhelminths
Of2DBD-NRA1     AHQVIQFARAVPGFRELPSVVMKKLVQEGMYPITVIQLSKDFQPSNMEYNYFNFTAQEREIILRHFPDFLPVADQLRVAGTVLHPFNLDETETALLCCIQLFHGGG-ERFGETSKVETMYQRTVSAIRTYEET------RQMA---EVRTQNILAVTSLLTDM
Cs2DBD-NRA1     AHQVIQFARAVPGFRDLPSVVMKKLVQEGMYPITMIQLSKDFQPSTMEYNYFNFTAQEREIILRHFPDFLPVADQLRVAGTVLHPFNLDETETALLCCIQLFHGGG-ERFGETSKVETMYQRTVSALRTYEET------RQMA---EVRTQNILAVTSLLTDM
Phe2DBD-NRA1    AHQVIHFARAVPGFRDLSRLDMKTLVQESMYPIVLIQLSRDFQFSTMEYNYFNFTPHEREIILEAFPAFRPIADHVRLAGNVLKPLALDDTETALYCTVQLFHGGG-EHFDEPAKIETMYKQAVAALRKYEQI------NFGS---DDRAQSILTVTSILAEM
Psm2DBD-NRA1    AHQVIHFARAVPGFRDLSRLDMKTLVQESMYPIVLIQLSRDFQFSTMEYNYFNFTPHEREIILEAFPAFRPIADHVRLAGNVLKPLALDDTETALYCTVELFHGGG-EHFDEPVKIETMYKQAVAALRKYEQI------NFGS---DDRAQSILTVTSILAEM
Pw2DBD-NRA1     AHQVIHFARAVPGFRDLSRLDMKTLVQESMYPIVLIQLSRDFQFSTMEYNYFNFTPHEREIILEAFPAFRPIADHVRLAGNVLKPLTLDETETALFCTVELFHGGG-EHFDEPARIETMYKQAVAALRKYEQI------NFGS---DDRAQSILTVTSILAEM
Eg2DBD-NRA1     AHQIIRFARAIPGFCDLPRADTKFLLQASMYPIVLVQLSREA-LPGGDFNFYNFSPHERRHLLAEFPQINPMVDHFYELTGFLGPLSLDNTEAALLCSIIFLRGSN-QKLLEAEKIYEIYNHAASALQQYITV------RYSS---DERFTQLIKLLPSLSSM
Em2DBD-NRA1     AHQIIRFARAIPGFCDLPRADTKFLLQASMYPIVLVQLSREA-LPGGDFNFYNFSPHERRHLLAEFPQINPMVDHFYELTGFLGPLNLDNTEAALLCSIIFLRGSN-QKLLEAEKIYEIYNHAASALQQYITV------RYSS---DERFTQLIKLLPSLSSM
Ta2DBD-NRA1     AHQIIRFARAIPGFRDLPRADTKFLVQASMYPIVLVQLSREP-LPGGDFNFYNFSPRERRHLLAEFPQINPLVDHFYELTGFLGPLNLDNTEAALFCSIIFLRGSN-QKLLEAGKIYEIYNHAASALQQYITV------RYSS---DERFTHLIKLLPSLSSM
Ht2DBD-NRA1     AHQIIRFARAIPGFRDLPREDTKFLVQASMYPIVLIQLSREP-LPGGDFNFYNFSPCERRHLLAEFPQINPLAEHFYELTNFLGPLNLDNTEAALLCSIIFLRGSN-QKLFEAGKIYEIYNHAASALQQYITV------RYSS---DERFTHLIKLLPSLSSM
Hm2DBD-NRA1     AHQVIRFARAIPGFRDLPRADTKFLVQASMYPIVLVQLAREP-LQGGDFNFYNFTPRERQHLLDEFPQLNPLAEHFYDLTNFLGPLNLDNTEAALFCSIIFLRGSN-QKLQEAEKIYEIYNHAALALQQYITV------RYSS---DERFTRLIKLLPSLSSM
Hd2DBD-NRA1     AHQVIRFARAIPGFRDLPRADTKFLVQASMYPIVLVQLAREP-LQGGDFNFYNFTPRERRHLLDEFPQLNPLAEHFYDLTNFLSPLNLDNTEAALFCSIIFLRGSN-QKLQEAEKIYEIYNHAALALQQYITV------RYST---DERFTRLIKLLPSLSSM
Hn2DBD-NRA1     AHQVIRFARAIPGFRDLPRADTKFLVQASMYPIVLVQLAREP-LQGGDFNFYNFTPRERQHLLDEFPQLNPLAEHFYDLTNFLGPLSLDNTEAALFCSIIFLRGSN-QKLQEAEKIYEIYNHAALALQQYITV------RYSS---DERFTRLIKLLPSLSSM
```

```
Spr2DBD-NRA1     AHQVIRFARAIPGFRDLPRSDTKVLVQESMYPIVLIQMSRDFNATSMEYNFFNFTPRERRYILTEFPIMRKVAAQLRDAGTFLRPKNMDENEIALLCCIVFLQHFN-RNLIESDRIYEMRAHAVSALRTYITV------QYST---EERFYSLMEVLPILTNM
Pw2DBD-NRA2      AQQIVQFAKLVPGFNHLGIAARSNLVRGAMYPVVLLLLSRDYQSETDEYNYFDFTLEERAIIVHHFPSFVRFTEHLRMAGRLLHHLGLSLPELSLSSAVEILRNYQLLDEPTRSNTKELFILAHHSLRACMTRRSDVSLEFDE---RHRWAQLTALSKMIQAM
Psm2DBD-NRA2     AQQIVQFAKLVPGFNHLGIAARSNLVRGAMYPVVLLLLSRDYQSETDEYNYFDFTPEERAIIMHHFPSFVRFTEHLRMAGRLLHHLGLSLPELSLSSAVEILRNYQLLDEPTRSNTKELFILAHHSLRACMTRRSDVSLEADE---RHRWAQLTALSKMIQAM
Cs2DBD-NRA2      AQQIVQFAKLVPGFNQLGITARSNLVRGAMYPVMLLLLSRDYHPDTDEYNYFDFTDEERAVIMHHFPTFQRFTEHLRMAGRLLHHLGLSLPELSLSAAVEILRNHQLLEEPTRTSTRELFVLAHHSLRVCMTRCSDCPPEQEG---RHRWAQLVALTKMLQAM
Of2DBD-NRA2      AQQIVQFAKLVPGFNQLGITARSNLVRGAMYPVMLLLLSRDYHPETDEYNYFDFTDEERAVIMHHFPTFQRFTEHLRMAGRLLHHLGLSLPELSLSAAVEILRNHQLLEEPTRTSTRELFVLAHHSLRVCMTRCSDCPPEQEG---RHRWAQLVALTKMLQAM
Ov2DBD-NRA2      AQQIVQFAKLVPGFNQLGITARSNLVRGAMYPVMLLLLSRDYHPETDEYNYFDFTDEERAVIMHHFPTFQRFTEHLRMAGRLLHHLCLSLPELSLSAAVEILRNHQLLEEPTRTSTRELFILAHHSLRVCMTRCSDCPPEQEG---RHRWAQLVALTKMLQAM
Fh2DBD-NRA2      AQQIVQFAKLVPGFNHLGITARSNLVRGAMYPVVLLLLSRDYRPDTDEFNYFDFTPDERATIMHHFPSYIRFTEHLRMAGRLLHHLGLSLPELSLLCAVEIVRNYQVLDEPTRSSTKELYILAHHSLRMYMTNNSDIGCDSDN---QHRWAQLVAMSKMIQAM
Fg2DBD-NRA1      THQIIQFARAVPGFRDLSRLDMKTLVQSGTYPILLLQLSRDQQTENMDCNYFDFSAVERPVVLRAFPILQRLADQLIGIGHMVNSLRLDQSEVGLLFCLALMHGDAHQELEEPNKVHAIYQQALLALEKHEQP------RTNP---DQRVRDLLAVIPVLIEM
Fbu2DBD-NRA1     EHQIIQFARAVPGFRDLSRMDMKTLVQSGTYPILLLQLSRDSLTENIDFNYFDFSAAERPVIMGAFPTLKRLADQLNGVGNMMSSLRLDQRETGLLCCLTLLHGDAHEQFDEPNKVQAIYQQALLALEKHEQT------RKDS---DQRVRDILAVIPVLMEM
Eca2DBD-NRA1     AHQITRFAREVPGFRDLSRVDMITLVQSGTFPIILLQLSREREGGSGEYNYFDFHSTERRIIVREFPTLQRLADQLCEIGHVMRTLRLDESEMGLLCCLVLLHADDHQQFEDPNRVQTTYQHALMVLEKYEQA------RANS---EQRIRDLLAVIPILDEM
Ov2DBD-NRA1      AHQVIQFARAVPGFRELPSVVMKKLVQEGMYPITVIQLSKDFQPSTMEYNYFNFTAQEREIILRHFPDFLPVADQLRVAGTVLHPFNLDETETALLCCIQLFHGGG-ERFGETSKVETMYQRTVSALRTYEET------RQMA---EVRTQNILAVTSLLTDM
Sma2DBD-NRA2     AQQVVQFAKLVPGFNQLGITARSNLVREAMYSVLLLLLSRDYCPETDEYNYFDFPAKEREVIMRHFPTFKRITEHLRVSGRIMHHLNLSLPELSLSCAAEILRNYCILEEPTAKSTAELFVLAHHSLLNCMAKQS-VPTVAST---QQRRTQLFALRKMIRVM
Sm2DBD-NRA2      AQQVVQFAKLVPGFNQLGITARSNLVREAMYSVLLLLLSRDYCPETDEYNYFDFPAKEREVIMRHFPTFKRITEHLRVSGRIMHHLNLSLPELSLSCAAEILRNYCILEEPTAKSTRELFVLAHHSLLNCMAKHS-VPTVAST---QQRRTQLFALRKMIRVM
Sj2DBD-NRA2      AQQIVQFAKLVPGFNQLGITARSNLVREAMYSVLLLLLSRDYCPETDEYNYFDFPPQEREVIMRHFPTFKRITEHLRVSGRVMYHLNLSLPELSLSCAAEILRNYCILEEPTASSTAELYVLAHHSLLNCMTKHS-IQTLVSI---QQRRTQLFALRKMIRVM
Hd2DBD-NRA2      SQQVVQFAKLIPGFNQMSLSARGHLVRASLYSEVLLMLSRDYDVTEDRYNFLDFSPAERDIILRHFPIYNRVVEHLKISGRFFQQLNLTHTEFVYACAIGILRQYYILANPAYTDARKLLLLARHSLLATMRSQT--ESQELV---DHKWNRLEALSEMLSSM
Hm2DBD-NRA2      SQQVVQFAKLIPGFNQMSLSARGHLVRASLYSEVLLMLSRDYDAAEDRYNFLDFSPAERDIILRHFPIYNRVVEHLKISGRFFQQLNLTHTEFVYACAIGILRQYFILADPAYTDARKLLLLARHSLLATMRSQT--ESQELV---DHKWNRLEALNEMLSSM
Eg2DBD-NRA2      SQQVVQFAKLIPGFNQMSLSARGHLVRASLYSEVLLMLSRDYDAAEDRYNFLDFSPTERDIVLRHFPIYNRVVEHLKISGRFFQQLNLTHTEFVYVCAIGILRYYYILANPAYMDARKLLLLARHSLLATMRSQN--ESQELV---DHKWSRLEAMSEMLSSM
Em2DBD-NRA2      SQQVVQFAKLIPGFNQMSLSARGHLVRASLYSEVLLMLSRDYDAAEDRYNFLDFSPTERDIVLRHFPIYNRVVEHLRISGRFFQQLNLTHTEFVYVCAIGILRYYYILANPAYTDARKLLLLARHSLLATMRSQN--ESQELV---DHKWSRLEAMSEMLSSM
Se2DBD-NRA2      SQQIVQFAKLIPGFNQLELTARGALVREGMYAAMLLLLARDYEPATGKYNYFDFSAEERDVILSYFPSFTRINDHLSITGRLYQELALTLPEFTLVCAIEILKNFAVLSGASCTHARRFFLLAKHSLLATMRNQA--SSREPV---EKRCAQLESFSQVLHDL
Sh2DBD-NRA1      IYEIIQFAQSIPSFQDLSEYDMKILIQQSIYPIILIQLSQDFSNN-KTKEYFYLNIQSQTSLINQFSVCKILFEQINLTNKLLKSLNLNETEIGLLCCVELFHGDG-KCLNEPIKVYETYQTILQLLKEYETN------QFNS---EKRFYKIMSIKHNLDRM
Sb2DBD-NRA1      IYEIIQFAQSIPSFQELSEYDMKILIQQSIYPIILIQLSQDFSNN-KTKESFYLNIQSQTSLINQFSVCKILFEQINLTNKLLKSLNLNETEIGLLCCVELFHGDG-KCLNEPIKVYETYQTILQLLKEYETN------QFNS---EKRFYKIMSIKHNLDRM
Sma2DBD-NRA1     IYEIIQFAQSIPSFQELSQYDMKILIQQNIYPIILIQLSQDFSNN-KTKEYFYLNIQNQTNLINQFSMCKILFEQINLTNKLLKSLNLNETEIGLLCCVELFHEDG-KCLNEPIKVNETYQTILQLLKEYETN------QFNS---EKRFYKIMSIKHNLDRM
Sm2DBD-NRA1      IYEIIQFAQSIPNFQELSEFDMKILIQQSIYPIILIQLSQDFNNN-KTKEYYYFNIQSQTSLINQFSVCKILFEQINLTNKLLKSLDLNETEIGLLCCVELFHTDG-KYLNEPIKINEAYQTILQLLKEYETISLSILDQFNS---EKRFYKIISIKHNLDRM
Sj2DBD-NRA1      IYEITQFAQTISSCQELSEFDMKILIQQSVYPIILIQLSHDFSN--KIKEYF--NIHSQICLLEQFPMCKILIEQIHLTEKLLKSIDLNETEIGLLCCIELFQSGG-GFLNEPIKVNETYQTILQLLKEYETN------RFNS---ELRYYEIMSIKNNLDKM
Fh2DBD-NRA1      THQIIQFARAVPGFRDLSRLDMKTLVQSGTYPILLLQLSRDQQTENMDCNYFDFSAVERPVVLRAFPILQRLADQLIGIGHMVNSLRLDQSEVGLLFCLALMHGDAHQELEEPNKVHAIYQQALLALEKHEQP------RTNP---DQRVRDLLAVIPVLIEM
hVDR             IQKVIGFAKMIPGFRDLTSEDQIVLLKSSAIEVIMLRSNESFTMDDMSWTCGNQDYKYRVSDVTKAGHSLELIEPLIKFQVGLKKLNLHEEEHVLLMAICIVSPDR-PGVQDAALIEAIQDRLSNTLQTYIRC------RHPPPGSHLLYAKMIQKLADLRSL
hVDR_cut         IQKVIGFAKMIPGFRDLTSEDQIVLLKSSAIEVIML------------------------------------------------------HEEEHVLLMAICIV--DR-PGVQDAALIEAIQDRLSNTLQTYIRC------RHP-----LLYAKMIQKLADLRSL
hRXRa            LFTLVEWAKRIPHFSELPLDDQVILLRAGWNELLIASFSHR-SIAVKDGILLATGLHVHRNSAHSAGVGAIFDRVLTELVSKMRDMQMDKTELGCLRAIVLFNPDS-KGLSNPAEVEALREKVYASLEAYCKH------KYPEQ--PGRFAKLLLRLPALRSI
hRXRa1_Cut       LFTLVEWAKRIPHFSELPLDDQVILLRAGWNELLIA------------------------------------------------------DKTELGCLRAIVLF--DS-KGLSNPAEVEALREKVYASLEAYCKH------KYP-----GRFAKLLLRLPALRSI

Phe2DBD-NRA3.1   SRFIVHFVKLIPGFNQLVLDDRRQLVRGAMYPLMLLELSRDYAKNEEFQFNYFDFTETERGVIFKHFPTFHTITRHLIQSGEFLDQVKLDNVELTLICAQEVFK-NYHGLIDPPATEHLYYLAGRVLVDHIVTSGQP--LLERSNRLRDLSPMLEQL
Pw2DBD-NRA3      SRFIVHFVKLIPGFNQLVLDDRRQLVRGAMYPLMLLELSRDYVKNAELQFNYFDFTETERGVIFKHFPTFHTITRHLIQSGEFLDQVKLDNVELTLVCAQEVFK-NYHGLIDPPATEHLYYLAGRVLVDHIVTSGQP--LLERSNRLRDLSPLLEQL
Of2DBD-NRA3      ARFVVHFVKLIPGFDQLELGDKRQLVRGAMYPLMLLELSRDFVNGEVVHFNYFDFTESERDVIHKHFPTFRAMVVHLIRSGKLLDDIRLDNVELALVCAQEVFK-HYHGLIDPPATQHLYNLASRALTDYIITTGQP--VEERSTLLRHLSSLLEEL
Fh2DBD-NRA3      SRFIVHFVKLIPGFNQLELSDKRQLVRKAMYPLMLLELSRDYVNGDSTQYNYFDFTEDEREVIFKHFPTFHTIIGHLIRSGEFLHRIRLDNIELVLVCAQEVFK-SQPGLIDSASADHLFSLASRALTNHIIDSGQP--LEERSSMLRLLSPLLEEL
Spr2DBD-NRA3     SKMVVNFTKIIPGINRLELNDKRQLVRSAMYPLMLVTLSRDFENGRYPRYNYFNFSEKEREVILQNFPAFKRVVNHLIQSGEMLHHLMLDDIEFTLICTQELLR-SQNDLSDPTSCQHLLLLSMQVLVNHEQSIAKPEIASQRLTAISQLLPMLNHF
Se2DBD-NRA3      SKMVVNFTKIIPGINRLELNDKRQLVRSAMYPLMLVTLSRDFENGRYPRYNYFNFSEKEREVILQNFPAFKRVVNHLIQSGEMLHHLMLDDIEFTLICTQELLR-SQNDLSDPTSCQHLLLLSMQVLVNHEQSIAKPEIASQRLTAISQLLPMLNHF
Eg2DBD-NRA3      TRMVVDFSKLIAGFNRLGINDRRQLIRAAMYPIMLIELSRDFQNNSSLSYNYFDFPEREKEIIIQRFPPLAKIISHLVQSGKVLRRLKLDDIESTIICIQELLR-HKNELEDPASCEHLFLLSMQALVNHEHQKSKSDAASERLTAFTQLLPILNQL
Ta2DBD-NRA3      TRMVVDFSKLIAGFNRLGINDRRQLIRAAMYPIMLIELSRDFQNNGSLSYNYFDFLEREKEIIIQHFPPLTKIISHLVQSGKVLQHLKLDDIESTIICIQELLR-HKNELEDPASCEHLFLLSMQALVNHEHLKSKSDAASERLTTFTQLLPMLNQL
Hm2DBD-NRA3      TRMVVDFSKLIAGFNGLGINDRRQLIRAAMYPIMLIELSRDFQNNENASYNYFDFMDHEKDVIIQHFPPLSKIIAHLVQSGRVLQQLKLDDIECTIICIQELLR-HKNELEDPASCEHLFLLSMQALVYHEQCKSKSDAANERLTTFTQLLPMLNQL
Sm2DBD-NRA3      SRFIVQFVKYIPGFCYLKISDQRQLVRSAMYPIMLLELSRDYVNEDRTRYNYFDFTPEEHAIILSHFPTFHKISGHLIRSGEFLTRLNLDNIELTLMCAQEVFK-DRQGLDDPVTPAYLFNLVGLALTEHIISVGYS--LEGRYAALSLISPMLEEL
Sh2DBD-NRA3      SRFIVQFVKYIPGFCYLKISDQRQLVRSAMYPIMLLELSRDYVNEDRTRYNYFDFTPEEHAIILSHFPTFHKISGHLIRSGEFLTRLNLDNIELTLMCAQEVFK-DRQGLDDPVTPAYLFNLVGQALTEHIISVGYS--LEERCAALSLISPMLEEL
Sj2DBD-NRA3      SRFIVQFVKYIPGFCYLKISDQRQLVRSAMYPIMLLELSLDYCHEDNSRYNYFDFTSEEHAIILSHFPTFRKICCHLIRSGEFLSRLNLDNIELTLICAQEVFK-DRQSLDDPVTPAYLHSLVRQVLADHIVSVGYS--LDERCSSLSLISPMLEEL
hVDR             IQKVIGFAKMIPGFRDLTSEDQIVLLKSSAIEVIMLRSNESF-TMDDMSWTCGNQDYKYRVSDVTKAGHSLELIEPLIKFQVGLKKLNLHEEEHVLLMAICIVSPDRPGVQDAALIEAIQDRLSNTLQTYIRCRHPPPGSHLLYAKMIQKLADLRSL
hVDR_cut         IQKVIGFAKMIPGFRDLTSEDQIVLLKSSAIEVIML-------------------------------------------------------HEEEHVLLMAICIV--DRPGVQDAALIEAIQDRLSNTLQTYIRCRHP-----LLYAKMIQKLADLRSL
hRXRa            LFTLVEWAKRIPHFSELPLDDQVILLRAGWNELLIASFSHR--SIAVKDGILLATGLHVHRNSAHSAGVGAIFDRVLTELVSKMRDMQMDKTELGCLRAIVLFNPDSKGLSNPAEVEALREKVYASLEAYCKHKYPEQ--PGRFAKLLLRLPALRSI
hRXRa1_Cut       LFTLVEWAKRIPHFSELPLDDQVILLRAGWNELLIA-------------------------------------------------------DKTELGCLRAIVLF--DSKGLSNPAEVEALREKVYASLEAYCKHKYP-----GRFAKLLLRLPALRSI
```

**Rotifera**

```
Rso2DBD-NRA4  ANRSVKFATRIPGFMNFHNVNDQIQLIKSSIHSIIILCLQRLNNSFIWNYFNIIDQKLYEQFQQIFPFIECIRNDGEKIQTYIYSLKLDEKEFSLLLSLLIISTSNIHLNDFLLIDSTQTELTCALCDYMDSKRGTKSRDFYTLMLLLPSLRKL
Rss2DBD-NRA1b ANRSVKFATRIPGFMNFHNVNDQIQLIKSSIHSIIILCLHRVNNSFIWNYFNVIDQKLHEQFQQIFPFIEHIRSDGEKIQAYIYSLKLDEKEFSLLLSLLIISTSNIHLNDFLLIDSTQTELTCALCDYMDSKRGSKSRDFYTLMLLLPSLRKL
Rs2DBD-NRA1   ANRCVKFATRIPGFMNFHNVNDQIQLIKSSIHSIIILCLQHLNNPSIWNYFNVIDQKMNEEFQQIFPFIEHIRSDGEKIQSYIYSLKLDEKEFSLLLSLLIISTSNVHLNDFLLIDSTQTELTCALCDYMDNKRGSKSRDFYTLMLLLPSLRKL
Ar2DBD-NRA1a  ANRAVKFATRIPNFMNFHTVTDQIQLIKSSIHSIVILCLQHTYPSSLWNYFNTIDEKFHDECQKLFPFIEYIRTDVEKLQTYMYALRLDEKELSLFLSLLIISTSNIRLNDFLLIDSTQTELTCALCDYMDCKRGSKSRDFYTLMLLLPSLKKL
Ar2DBD-NRA1b  ANRSVKFATRIPGFMNLQNVTDQVQLIKSSIHSLIILCSHRMLKSSSWNYFNVTETESPEAFQNFFPFIENIRFDVEKVHTYISALKLDEKEFTLFLSLLIISTSNIYLSEFLLIDSTQTELTCALCDYMDCKRGRKNRDFYTLMFLLPTLRKL
As2DBD-NRA1b  ANRTVKFATRIPGFMNLQNVTDQVQLVKSSIHSLIIVCLQRLNNCFLFNYFNTIDEKLNNEFEKLFPFIKTNYSDIEKIQIYISSLKLDEKEFSLLLSLLLVSTGNTYLNDFLLIDSTQTQLTILLSDYMDCKRGEKNRDFYTVMFLLPTLRKL
hVDR          IQKVIGFAKMIPGFRDLT-SEDQIVLLKSSAIEVIMLRSNESFTMDDMSWTCGNQDYKYRVSDVTKAGHSLELIEPLIKFQVGLKKLNHEEEHVLLMAICIVSPDRPGVQDAALIEAIQDRLSNTLQTYIRCRHPPPGSHLLYAKMIQKLADLRSL
hVDR_cut      IQKVIGFAKMIPGFRDLT-SEDQIVLLKSSAIEVIML------------------------------------------------------HEEEHVLLMAICIV--DRPGVQDAALIEAIQDRLSNTLQTYIRCRHP---LLYAKMIQKLADLRSL
hRXRa         LFTLVEWAKRIPHFSELP-LDDQVILLRAGWNELLIASFSHRSIAVKDGILLATGLHVHRNSAHSAGVGAIFDRVLTELVSKMRDMQMDKTELGCLRAIVLFNPDSKGLSNPAEVEALREKVYASLEAYCKHKYPEQPGRFAKLLLRLPALRSI
hRXRa1_Cut    LFTLVEWAKRIPHFSELP-LDDQVILLRAGWNELLIA------------------------------------------------------DKTELGCLRAIVLF--DSKGLSNPAEVEALREKVYASLEAYCKHKYP---GRFAKLLLRLPALRSI

Ar2DBD        SICVRNFVDGIPNFNSLNVEDKTSLVYQSIHSVMLMCFCIQSVRSE--TSNSTWNYLNLSSNSP-FYQYIQQQFPFFFELFSSTRAFENDLQRLDLDDKEIALILTLLITSIGDYKSTPI---EHLEEEFFSILLDYMTAHRGIN--SKDYFILTIRIPFVHRL
Ar2DBD-NRA2b  SICVRNFIEGIPNFNSLNVEDKTSLVYQSIHSVMLMCFCIQSVRSE--TSNSTWNYLNLSSNSP-FYQYIQQQFPFFFELFSLTRAFESDLQRLNLNDKEIALVLILLITSIGDYKSTPI---EHLEEEFFSILFDYMTVQRGIN--SKDYFILTIRIPFVHRL
Av2DBD        SNCVRNFTDEISSFNSLNFEDKTSLIHSSAYSVILMCFSLQSTRLG--TLQNSWNYLNVPSDFS-YYQYIQQQFPFFFKLSDLTNRFEKDLQILELNDKEIAIILALLIISIGNDKSKYI---EMIAEEFFAMLYDYMSAKHGIN--SKDYFILTIQIPFLHRL
Rsp2_2D3      SNRVRDFIDRIPNFNSIHFIEKTSLTHLSTHSVILMCLCIHSLRLR--TINTNWNYFNISSNSS-INQYVQERFPFFFELNRLTYSFQKELQILELDDKEISLMLTLLITSIGNDKLKEI---ENIEEEFFSILYDYMAVKRGID--NKDYFILTIQIPLLHRI
Rso2DBD-NRA2b SNRIRDFIDKIPNFNSISFIEKTSLIHLSNHSVILMCLCIQSLRLR--TINRNWNYFNISLNSSSFSQYLQEHFPFFFELNHLTYSFEKELQILELDDKEISLMIILLITSIGNNKLKEI---ENIEEEFFSILHDYMAVKRGID--NKDYFILTIQIPFLHRI
Rm2DBD-NRA2b  SNRVRAFIDRIPNFNSINFIEKTSIIHLSTHSVILMCLCIQSLRSG--IVNSNWNYLNISFNSS-HSQYLQDPYPFFFELNRLTYSFEKELQIFELDDKEIALMLALLITSIGNNKSKEI---EYLEEELFTILYDYMAVKRGMN--NKDYFILTIQIPFLHRI
Rs2DBD-NRA2b  SNRVRAFIDRIPNFNSINFIEKTSIIHLSTHSVILMCLCIQSLRSG--IVNSNWNYLNISFDSS-HSQYLQDQYPFFFELNRLTYSFEKELQIFELDDKEIALMLTLLITSIGNNKSKEI---EYLEEELFTILYDYMAVKRGMN--NKDYFILTIQIPFLHRI
Rm2DBD-NRA2a  TARVGDFALRIPEFCSLTADDKNLLIHSSTHSVIVACLCFQASRFRSLTNDSQWNYLNILTNCS-FGQSLQQNFPFFFDLNQITYSFEREIQLLELDDREVALMIVLLITSIANSKLNETEKIEKIQEDFFCALYDYMSAKRGID--SKDYFILTIQIPLIHRI
Rs2DBD-NRA2a  TARVGDFALRIPEFCSLTADDKTLLIHSSTHSVIVACLCFQASRFRSLTNDSQWNYLNILTNCS-FGQSLQQNFPFFFDLNQITYSFEREIQLLELDDREIALMIVLLIASIANSKLNETEKIEKIQEDFFCALYDYMSAKRGID--SKDYFILTIQIPLIHRI
Rss1NR7A2a_   TTRVGDFALRIPDFCTLNSEDKSLLIHISTHSVILLCLGFQSSRYRTLTNDSNWNYLNILTNSS-FGQSLQQNFPFFFDLNHLTYSIERELQILELDDKEIALMIVLLITSITNSKLKDIEKIDKLQENYFCALYDYMSAKRGID--SKDYFILTIQIPFIHRI
Rso2DBD-NRA2a TIRVGDFALRIPDFCSLNSEDKNLLIHSSTHSVILLCLCFQSVRFRTLTNDTNWNYLNISINSS-FGQNLQQNFPFFFDLNQLTYSIEHELQILELDDKEIALMIVLLITSIANSKLKEIEKIEKIQEDYFCALYEYMAAKRGID--NKDYFILTIQIPFIHRI
Ar2DBD-NRA2a  TKRVGDFALTIPEFSSLNSDDRTLLIHSSTHSVIILCLCLQSPRFRTLTNDLNWNYLNISVNSP-CGQYLQQMFPFLFDLEQFTYSFEKELQLLELDDRENALFLALLLVSVANSKFIEIDKLEKIQENIFCTLYDYMSAKRGVN--SNDYFILTVQIPFIHRI
As2DBD-NRA2   TARVRDFTLNITDFCILNSDDKNLLIDLSTHSVIVLCLCLQSSRFQ----NLNWNYLNISINST-YGQYLQQTFPFLFDLNQLTYSMEKEFQLLELDDKEIALFIILLITSIPNSKFKEM---EKIQEDFFCILYDYMSAKRGIH--NTDYFILTVQIPFLHRI
hVDR          IQKVIGFAKMIPGFRDLTSEDQIVLLKSSAIEVIMLRSNESFTMDD-----MSWTCGNQDYKYR---VSDVTKAGHSLELIEPLIKFQVGLKKLNLHEEEHVLLMAICIVSPDRPGVQDAALIEAIQDRLSNTLQTYIRCRHPPPGSHLLYAKMIQKLADLRSL
hVDR_cut      IQKVIGFAKMIPGFRDLTSEDQIVLLKSSAIEVIML-----------------------------------------------------------HEEEHVLLMAICIV--DRPGVQDAALIEAIQDRLSNTLQTYIRCRHP-----LLYAKMIQKLADLRSL
hRXRa         LFTLVEWAKRIPHFSELPLDDQVILLRAGWNELLIASFSHR-SIAV-----KDGILLATGLHVH---RNSAHSAGVGAIFDRVLTELVSKMRDMQMDKTELGCLRAIVLFNPDSKGLSNPAEVEALREKVYASLEAYCKHKYPEQ--PGRFAKLLLRLPALRSI
hRXRa1_Cut    LFTLVEWAKRIPHFSELPLDDQVILLRAGWNELLIA-----------------------------------------------------------DKTELGCLRAIVLF--DSKGLSNPAEVEALREKVYASLEAYCKHKYP-----GRFAKLLLRLPALRSI

Rso2DBD-NRA3a -QAIMNFCQN----FTNMS--NKYEIISSSINSVIMITLLPDKTLMKSSYEI--IQSTWNYWQAESTLSMELNAYVPQLTQAEHLFRSYESKLRNLQLDEKEFSLLLLMIITRNNLNLVINESNKSWSKYQYECIQAFCEYTQARRSNINREFSIEIYDIIFLVSQLRSL
Rss2DBD-NRA3a -RAVMNFCQSTIPDFTNMP--DKYEIISSSINSVIMVTLLLGKTLMKSSYEI--IQSTWNYWQAESTLSMELNTHVPQFIQTEDLFRSFESKLRDLHFDEKELSLLLLMIITRNNLNLVINETNKSWPKYQYECLQAFCEYTQARRSNINREFSIEVYDVMFLVSQLRSL
Rss2DBD-NRA3b -QAMMNFCHS----FINMS--NKYDIISSSINSVIMVTLLPGKASMKYSYEN--IQSTWNYWQAESTVSIELNALVPQLTQAEDLFRSFESKLRDLHLDEKEFSLLLLMIITRSKFNSVNENNRSWSKCQYECIETFSEYYQARRSNINRELPIEFYDIIFLVSQLRSL
Rm2DBD-NRA3a  -QAVMNFCQKIIPDFTSMP--DQWEIISSSINSVIIIKLLYDKILLNSLHEN--NQSTWNYWHAEATLSMELSAHAPQLMQAEDVFRSFELQFKDLLLDEKELSLLLLMLITRKNCNLMTENHKSWLKLQYECVEALAEYTQARRSNINPRRSIEFCDIIFLVSQLRSL
Rs2DBD-NRA3a  -QAVMNFCQKIIPDFTSMP--DQWEIISSSINSVIIIKLLYDKILLNSLHEN--NQSTWNYWHAEATLSMELSAHAPQLMQAEDAFRSFELQFKDLLLDEKELSLLLLMLITRKNCNLMTENHKSWLKLQYECVEALAEYTQARRSNINARRSIEFCDIIFLVSQLRSL
Ar2DBD-NRA3a  -QAVMNFCQKTIPGFDSMY--EKYDVISSCIHSVIMVALLPRILSIKSSYN--DLQPIWNYWQAESTLSIELNAHVPQLTEAENSFRSFETKIRDLHLDEKEFALLLLMIITRTNINVMNEETKFWPKCQYECVQAFSEYAQARRSNSIRGLSFEFYDIAFLVSQLRTL
As2DBD-NRA3a  -QAVMSFCQKTIPGFDNMY--EKYEVISSCINSVIMVALLPKTVSMRSSYG--DIQSTWNYWQAESTLSIELNAHVPQLTEAENSFRSFETKLRDFYLDEKELSLLLLMIITRTNLNVINEENKSWPKCQYECVQAFSEYTQARRLSNGCELPFEFYDIAFLVSQLRTL
As2DBD-NRA3b  -EAVINFCQKTIPGFDNIY--EKYEIISSSINSVIMITLLSKMTSTKYSYNNNNIQPIWNYWQAESTLSAELNAHVPQLTEAENEFRSFESKIKDLHLDEKELSLLLLMIITRTNLNVINEENNSWPKCQYECVQAFSEYTQARRSTTNEHLPLEFYDIIFLVSQLRSL
Rss2DBD-NRA3b -QALMNFCQKTIPGFTNMS--QKYEIISSSINSVIMVTLLMGKMFIKSSYEN--IQSTWNYWQAESTLSMELNAYIPQLTQAENLFRSFESTLRDLRLDEKEFALFLLMIITRHNLNLINEDNKLWSKCQYECVQAFSEYTQARRLNINGELSIEFYDIVFLVSQLRSL
Rss2DBD-NRA3c -QALMNFCQRTIPGFINMS--QKYEIISSSINSVIMVTLLSGKTLMKSSYEN--IQPTWNYWQAESTLSMELNAHVPHLTQAEYLFRSFESTLRGLRLDEREFSLLLLMIITRHNLNLINEDNKSWSKCQYECVEAFSEYTQAHRLDINGEISSEFYDIVFLVSQLRSL
Rso2DBD-NRA3b -QAMMNFCQRTIPGFTNMS--NKYEIISSSINSVIMVTLLAEKTFLKSSYEN--IQSIWNYWQADSTLSMELNTHIPQLTQAENLFRSFESTLRDLHFDEKEFSLLLLMIITRHNLSLINDDDKSWSKCQYECVHAFSEYIQARRSNINGELSIEFYDIVFLVSQLRSL
Rm2DBD-NRA3b   QAIMNFCQSIIPGFSNTS--DKYEIISSSINSVIMVTLLRSKILTKSSHEI--IHSIWNYWQAEPTLSMELNAHVPRLAQAENLFLSFESKLRHLRLDEKEFSLLLLMIITRNQLNVINGDNKLWSTYQYECVQAFSEYSQARRSKINREFSIEFYDIAFLVSQIRSL
Dc2DBD-NRA3b_ -ECLIHFCQKTITNFNNLS--DPYEIISSSIHTVIIITLMLTSRPKNKLIIG------WNYWKTDKTLADVLYSYIPYMSEAENFYRSNETELRNFQFDENEISLVILICLTRINF-LNSDKNSTWSKCQFDCVEALSEYMQARRG-ICDQLPFEFYDVSFLITQIRSL
hVDR          IQKVIGFAKM-IPGFRDLTSEDQIVLLKSSAIEVIMLRSNESFTMDDMSWT---------CGNQDYKYRVSDVTKAGHSLELIEPLIKFQVGLKKLNLHEEEHVLLMAICIVSPDRPGVQDAAL-IEAIQDRLSNTLQTYIRCRHP--PPGSHLLYAKMIQKLADLRSL
hVDR_cut      IQKVIGFAKM-IPGFRDLTSEDQIVLLKSSAIEVIML--------------------------------------------------------------HEEEHVLLMAICIV--DRPGVQDAAL-IEAIQDRLSNTLQTYIRCRHP-------LLYAKMIQKLADLRSL
hRXRa         LFTLVEWAKR-IPHFSELPLDDQVILLRAGWNELLIASFSHR-SIAVKDGI---------LLATGLHVHRNSAHSAGVGAIFDRVLTELVSKMRDMQMDKTELGCLRAIVLFNPDSKGLSNPAE-VEALREKVYASLEAYCKHKYP--EQ--PGRFAKLLLRLPALRSI
hRXRa1_Cut    LFTLVEWAKR-IPHFSELPLDDQVILLRAGWNELLIA--------------------------------------------------------------DKTELGCLRAIVLF--DSKGLSNPAE-VEALREKVYASLEAYCKHKYP-------GRFAKLLLRLPALRSI

Bc2DBD-NRA4a   SDKCYSFISNIPYITKFSDIDKNIIYVNSFHPIRILQLISLQQKLEQIETQPNLNSNINYFNCDTATYEKIIKHFPVFEKIMTYFRPLLDVVRELKLDSKELALYSAFLAFSASCKGLTGIREKFDCNLELCNALSKYMYLRRNENESSEKLINIAPRFERLNLQ
Bc2DBD-NRA4b   LEQCFYFIKQTKFLRKFNENDQNIILINSIHSLRLLQLISLEQNSSELEFQ------LNYFNCCKITYEKITKFWPIFEQIMVYFKPLVEYVRELKLDVNEFAIYSLFLVFSSSCKNLIGFREKFECNLELCNLLCKYMYLRRNENESSEKLINIAPRFERINLQ
hVDR           IQKVIGFAKMIPGFRDLTSEDQIVLLKSSAIEVIMLRSNESFTMDDMSWTC---------GNQDYKYRVSDVTKAGHSLELIEPLIKFQVGLKKLNLHEEEHVLLMAICIVSPDRPGVQDAALIEAIQDRLSNTLQTYIRCRHPPPGSHLLYAKMIQKLADLRSL
hVDR_cut       IQKVIGFAKMIPGFRDLTSEDQIVLLKSSAIEVIML---------------------------------------------------------------HEEEHVLLMAICIV--DRPGVQDAALIEAIQDRLSNTLQTYIRCRHP-----LLYAKMIQKLADLRSL
hRXRa          LFTLVEWAKRIPHFSELPLDDQVILLRAGWNELLIASFSHR-SIAVKDGIL---------LATGLHVHRNSAHSAGVGAIFDRVLTELVSKMRDMQMDKTELGCLRAIVLFNPDSKGLSNPAEVEALREKVYASLEAYCKHKYPEQ--PGRFAKLLLRLPALRSI
hRXRa1_Cut     LFTLVEWAKRIPHFSELPLDDQVILLRAGWNELLIA---------------------------------------------------------------DKTELGCLRAIVLF--DSKGLSNPAEVEALREKVYASLEAYCKHKYP-----GRFAKLLLRLPALRSI
```

**Mollusca and other**

```
Ls2DBD-NRA    ARSVLKFTKKVPGVRQLPIDTQIMMVQQAMYPIVLLNYSLEYDIEQQDYNYFSLSHEEETYIQ-----SYFPPFKMLIPTFHMLGHIVQTLKVDEIECAFLCGLCLLS---------QSDGTYGYQEALVSAMAVYCERKAL---TGSERVGLLLLRLCEMLRC
hVDR          IQKVIGFAKMIPGFRDLTSEDQIVLLKSSAIEVIMLRSNESFTMDDMSWTCGNQDYKYRVSDV-----TKAGHSLELIEPLIKFQVGLKKLNLHEEEHVLLMAICIVS--PDRPGVQDAALIEAIQDRLSNTLQTYIRCRHP-PPGSHLLYAKMIQKLADLRSL
Ga2DBD-NRA    AKCTITFAKKVPGFKSLVIEDQILLMQLATYPISLLNFACQYDVNKGDNASLCFTRAEEKMLV-----KFFPMLNILPAHHNHIGFMMKHLGIDPTELSIMCALILFC--DECEILKENWKVKEWHDYYIECLQEYTMSAYT---DGETRFGALLLRLGELTMN
Aca2DBD-NRA   AKCIVRFAKKICGFRSLDINDQVLLLRMATYSLVLLNHSRGYDVATGFYTYFNFTKKEQTRLR-----ELFPDFEVLHSHFAHAGIVAKCLGITDMEYAYLSCLLLLN--DELPDLAKAEEVKELRVRFMGSFQDYEIDNFP---NGSLRFGEMMLRLTEYYQF
Cu2DBD-NRA    SQRIVPFAKSRC-FRGLKIDDQVLLLRVATYSLVILNHSRAYEPETGFYNYFNLTQRETHMIS-----NLM--LS--HAHYKHIGSLLQKQGLTEVEYAYMSCILLLN--NEYPGLENTVMTQKLKERYVNAFLDYEIEHFP---KGRLRFAEMLLHLSEFSQL
Ac2DBD-NRA    AKCIVRFAKKICGFRSLDINDQVLLLRMATYSLVLLNHSRGYDVATGFYTYFNFTKKEQTRLR-----ELFPDFEVLHSHFAHAGIVAKCLGITDMEYAYLSCLLLLN--DELPDLAKAEEVKELRVRFMGSFQDYEIDNFP---NGSLRFGEMMLRLTEYYQF
Ech2DBD-NRA   AKIMVRYAKKTSGFRDLKLDDQVKLLSGATYNLVLLNHTRAYEPDTGFYNYFNMTKRNWMKVL-----EYFPEFAVLHTHCRHCGIMAKEIGLTEKEYAYMSSMILLD--DECEGIEEIHKVRELKDIFLSAFQHYESENFP---KGSLRFGQMLLRLSEFSQF
Cg2DBD-NRA    SGELVAALKMFPVFKILELDDRITLVQDSIYSMAILNFSRLFNVETKEYSFFGWSKEEEKKII-----EFYPELKTVQEDLLYVGDLIKTVAFDDIELTCLTGLLCFN--ADACNLKKRAKVEESQKYIMSFLEMYEEKKYN---NWELRFGMLVLRIGDLMGL
Cv2DBD-NRA    SGELVAALKMFPVFKILELDDRITLVQDSIYSMAILNFSRLFNVETKEYSFFGWSKEEEKKII-----EFYPELKTVQEDLLYVGDLIKTVAFDDIELTCLTGLLCFN--ADACNLKKRAKVEESQKYIMSFLEMYEEKKYN---NWELRFGMLVLRIGDLMGL
Cv2D1         SGELVAALKMFPVFKILELDDRITLVQDSIYSMAILNFSRLFNVETKEYSFFGWSKEEEKKII-----EFYPELKTVQEDLLYVGDLIKTVAFDDIELTCLTGLLCFN--ADACNLKKRAKVEESQKYIMSFLEMYEEKKYN---NWELRFGMLVLRIGDLMGL
My2DBD-NRA    ARKLIKFSKKVPGFRAISLDDQIKLIQASIYPIVVLNCSRSFDNETKQFSYFNYTPRQRETIQ-----SFFPMLKILSSHFIHTGTMVKLMNFSSMEYTFLSILLLLN--ADVENLEDSERVRKLLLDTTFALQYHEETTYT---DGSTRFGMLLVRLAELHFI
My2D1         ARKLIKFSKKVPGFRAISLDDQIKLIQASIYPIVVLNCSRSFDNETKQFSYFNYTPRQRETIQ-----SFFPMLKILSSHFIHTGTMVKLMNFSSMEYTFLSILLLLN--ADVENLEDSERVRKLLLDTTFALQYHEETTYT---DGSTRFGMLLVRLAELHFI
Pm2DBD-NRA    ARTLIKFSKKVPGFRALSLDDQIKLVQGSIYPIVVLNCSRSFDNETKQYSYFNFTPRQREAIH-----SFFPMLRILSSHFIHTGSMVKLMNLSTMEYTFLSILLLLN--ADVEDLDDSESVRKLQMDTTFALQYHEESNYP---DGMTRFGMLLVRLAELHFI
Mc2DBD-NRA    SSVIIKFAKKCQPFRKLALEDQVRMLQQALYPISILNHCRYYILETKQYSYFGWSVVEREHIW-----KYFPFYRILGEHFENTGDNVKLLGLDDDEFTILSTLVLLN--PDVPGLIDKEKVRQLQAELLDFLEYYLSITYP---DQPSRYGLVLVRLSELNFY
Mg2DBD-NRA    SSVIIKFAKKCQPFRKLALEDQVRMLQQALYPISILNHCRYYILETKQYSYFGWSVIEREHIW-----KYFPFYRILGEHFENTGDNVKLLGLDDDEFTILSTLVLLN--PDVPGLIDKEKVRQLQAELLDFLEYYLSITYP---DQPSRYGLVLVRLSELNFY
Me2DBD-NRA    SSVIIKFAKKCQPFRKLALEDQVRMLQQALYP-------------------------------------------------I--N--------DD-EFTILSTLVLLN--PDVPGLIDKEKVRQLQAELLDFLEYYLSITYP---EQPSRYGLVLVRLSELNFY
Mm2DBD-NRA    TYITIRFAKLVPGFKTLSLNDQVKLIQSSIYPIELLNMSKVYDPTTKKYNYFTYTKEEENIMM-----EQFPMLRVFQEHFLHVGEMVTSFNFTDEEFAILSALLLFP--AEANGLENPKAVEQLQNQISAALQAYEEREFP---EGLTRYGILLVRVAELVQC
Mm2D1         TYITIRFAKLVPGFKTLSLNDQVKLIQSSIYPIELLNMSKVYDPTTKKYNYFTYTKEEENIMM-----EQFPMLRVFQEHFLHVGEMVTSFNFTDEEFAILSALLLFP--AEANGLENPKAVEQLQNQISAALQAYEEREFP---EGLTRYGILLVRVAELVQC
Dp2DBD-NRA    TYITIRFAKLVPGFKGLCLNDQVKLIQSSIYPIELLNMSKVFDPDTKKYNYFTYTKEEENIMM-----DHFPMLRVFQEHFLHIGEMVTEFHFTDEEFAMLSALLLFP--SEIHGLEDPKAVEAMQTEVSAALQYYEEVTFP---DGLTRYGILLVRVAELVQC
Pc2DBD-NRA    AQCIIRFAKRVPGFRKLPLDDQVLLVQLAIYPIVILNHSRTYNVKTGFYNYFNFTKSEEDAIF-----KVCPGLNILRDHFKHMGVMTQMMKLDNVEYTLLSCMLLVD--SECPGIVEKEKALELQTKLLKVFQLYE-ER-----SGKCRFGELMLLISELSLA
Pca2DBD-NRA   AQCIIRFAKRVPGFRKLPLDDQVLLVQLAIYPIVILNHSRTYNVKTGFYNYFNFTKSEEDAIF-----KVCPGLNILRDHFKHMGVMTQMMKLDNVEYTLLSCMLLVD--SECPGIVEKEKALELQTKLLKVFQLYE-ER-----SGKCRFGELMLLISELSLA
Bg2DBD-NRA    AKCIVRFAKKVPGFRLLKIEDQVLLLRTATYGLVILIHSREYEPETGFYNYFNFCCVEQDKIM-----ELFPEFQILLSHFKHTGVMAKRLVLTDLEYAYLSCMLLLN--DEYQGLIDTKKVKELKEKYMEAFHEYEFHNFP---NGDLRFGEMLLRLSEFSQF
Bt2DBD-NRA    AKCIVRFAKKVPGFRQLKIEDQVLLLRMATYSLVILTHSREYEPDTGFYNYFNFTGNEMERIL-----ELFPEFDVIPGHFKHTGVMAKRLLLSDLEYAYLSCMLLLN--DEHQGLIDTKKVKELKEKYVEAFHNYEIESFP---SGDLRFGEMLLRLSEFSHF
Po2DBD-NRA    AKAMVRYAKKVPGFRDIKLDDQVRLLSKATYNLVLLNHTRAYEPATGFYNYFNMPRRNWLHCLVSSAWEYFPEFDVLHNHFKHCGILAKELGLTEIEYAYMSCIMLLD--DECEGLESVPQVKELKSIILAAFQQHQLENFP---SGSLRFGQLLLRLSEFSQF
Dg2DBD-NRA.   AQGVLRFAKKVPGFKDIHVEDQIVMVQFATYQIVMIMLAMDYNIERREYNYFNLSPSEKETML-----TTFTPLRSLLMHFHKIGFDVKRLQLDDIEVAVLCAIVLITNVPENEFKTGKQIIETVSAIQHGFLRRYCESKFRNAELGLSRQNDMLSLLTDLKET
La2DBD-NRA    VHCLIKFGKKIPGFRAIPMEDQICLVRHTNYIMSLAMMSQMFDLQTKAPNFYNLSVPMQKRIL----EAFSQPFAHVQEHMVSFGERLKSMDMDDIEYPMLCALILLQ--TSDLELKEPGKIDQLSAKVSNAFKAYMTNKYG---PNSPRYFSIIQILQDLHQA
Ema2DBD-NRA   AKVLVRFAKKVPGFRDLKLDDQVKLLRSASYSCVLLNHTREYEPATGFYNYFNIPQKACIKLQ-----ELYPEFDVLHTHHKHCGILTKELNLTEKEYGYMSCMLLVA--ECECEGLDDPDRVRQLKDIIMAAFQQYEFDNFP---NGALRFGQTLLRLSEFSQF
hVDR_cut      IQKVIGFAKMIPGFRDLTSEDQIVLLKSSAIEVIML--------------------------------------------------------HEEEHVLLMAICIV----DRPGVQDAALIEAIQDRLSNTLQTYIRCRHP------LLYAKMIQKLADLRSL
hRXRa         LFTLVEWAKRIPHFSELPLDDQVILLRAGWNELLIASFSHR-SIAVKDGILLATGLHVHRNSA-----HSAGVGAIFDRVLTELVSKMRDMQMDKTELGCLRAIVLFN--PDSKGLSNPAEVEALREKVYASLEAYCKHKYP-EQ--PGRFAKLLLRLPALRSI
hRXRa1_Cut    LFTLVEWAKRIPHFSELPLDDQVILLRAGWNELLIA--------------------------------------------------------DKTELGCLRAIVLF----DSKGLSNPAEVEALREKVYASLEAYCKHKYP------GRFAKLLLRLPALRSI
```

**Chordata**

```
Bb2DBD-NR7A   VHVTTKFAKKIPGFRTCSIDDQISMIQGAAFPINSCILALDYNPVTNEMNYFNMTEMERTAMP-----RLFPPFAQLPVKLPDIWKKILALRMNKAEMTLFCGILLMS--PDTAGLKDPKGVEMLQGKLYSILEKYTLAQGVIGGV--SGVVRFQAMLQAIMALKKL
Bf2DBD-NR7A   VHVTTKFAKKIPGFRTCSIDDQISMIQGAAFPINSCILALDYNPVTNEMNYFNMTEMERTAMP-----RLFPPFAQLPVKLPDIWKKILALRMNKAEMTLFCGILLMS--PDTAGLKDPKGVEMLQGKLYSILEKYTLAQGVIGGV--SGVVRFQAMLQAIMALKKL
Bl2DBD-NR7A   VHVTTKFAKKIPGFRTCSIDDQISMIQGAAFPINSCILALDYNPVTNEMNYFNMTEMERTAMP-----RLFPPFAQLPVKLPDIWKKILALRMNKAEMTLFCGILLMS--PDTAGLKDPKSVEMLQGKLYSILEKYTLAQGVIGGV--SGVVRFQAMLQAIMALKKL
hRXRa         LFTLVEWAKRIPHFSELPLDDQVILLRAGWNELLIASFSHR-SIAVKDGILLATGLHVHRNSA-----HSAGVGAIFDRVLTELVSKMRDMQMDKTELGCLRAIVLFN--PDSKGLSNPAEVEALREKVYASLEAYCKHKYP----EQ--PGRFAKLLLRLPALRSI
hRXRa1_Cut    LFTLVEWAKRIPHFSELPLDDQVILLRAGWNELLIA--------------------------------------------------------DKTELGCLRAIVLF----DSKGLSNPAEVEALREKVYASLEAYCKHKYP---------GRFAKLLLRLPALRSI
```

**2DBD-NRB**
**Phoronida**

```
Pa2DBD-NRB    NEAQLWFATRIPGFCNLCREDQMILLKYGSFQIGMVWASLRMFDPNTGKFHSFDCTKLP-----AGHPMGLFQTQLCSLSWKLNTMAMDDTEASMLCSLILLSEDYLELTDTDKIHKLYSMMLKALDAYVYRKIGKK--SNRLSDLVSFIPKLRHL
hVDR_cut      IQKVIGFAKMIPGFRDLTSEDQIVLLKSSAIEVIML----------------------------------------------------HEEEHVLLMAICIV--DRPGVQDAALIEAIQDRLSNTLQTYIRCRHP-----LLYAKMIQKLADLRSL
hVDR          IQKVIGFAKMIPGFRDLTSEDQIVLLKSSAIEVIMLRSNESFTMDDMSWTCGNQDYKYRVSDVTKAGHSLELIEPLIKFQVGLKKLNLHEEEHVLLMAICIVSPDRPGVQDAALIEAIQDRLSNTLQTYIRCRHPPPGSHLLYAKMIQKLADLRSL
hRXRa         LFTLVEWAKRIPHFSELPLDDQVILLRAGWNELLIASFSHR-SIAVKDGILLATGLHVHRNSAHSAGVGAIFDRVLTELVSKMRDMQMDKTELGCLRAIVLFNPDSKGLSNPAEVEALREKVYASLEAYCKHKYPEQ--PGRFAKLLLRLPALRSI
hRXRa1_Cut    LFTLVEWAKRIPHFSELPLDDQVILLRAGWNELLIA----------------------------------------------------DKTELGCLRAIVLF--DSKGLSNPAEVEALREKVYASLEAYCKHKYP-----GRFAKLLLRLPALRSI
```

**Mollusca**

```
Ap2DBD-NRB    IIGSVTFAKNIPGFRHISVNDQMNLIKAGTFPCILCQLCWQGC-----MFSWFDDTFFTVPSLIKLVCG--MKTSCYSFREDFTKLTFDQEEIALFTALIFINPDNPGLDDVEFILGLHGKLRQVFKHYCLEKYGELKQYLKFISFLPRLYQIELL
Pmi2DBD-NRB   IIGSVNFAKNIPGFREIPVSDQMNLIKLGTFPSILCHLCWQGE-----EYSWLEHTFFTVPSLEALVCG--MKTSCYSFREDFSKVTFDEHEIALIMALIFINPDPSGLESVDFILNLHGKLRLIFKHYCLEKYGEMKQYLKFISFLPRLYKIELL
Aru2DBD-NRB   IISSVKFAKNIPGFCHISVSDQMSLIKMGTFPCILSHICWRAEL----EFSWFQDTFFTVPSLDALVFG--MKSKVYKFKEDFIQVKFDQHEIALFTTLIFINPDPPGLENSDYILKLHNKLRLILKHYCLEKYGELKQYYKYLSFLPRLYEIELV
Lv2DBD-NRB    IIASVQYIKKIPGFASIDMNDRINLIKHSVFPIILVHKAWKMGE----EWKWMEDTFFTNSSLRKMSDG--FKDMTYSFINQFNLGSFDSIEVALLMILVTLNPDLEGLENKEAVLKLHKDYRTIMQNYCLETHGKLDNYLIMLQFVPYLSKIDIV
Spur2DBD-NRB  IIASVQYIKKIPGFPSIHMNDRLYLIKHSVFPIILVHKAWKMGQ----EWKWMEDTFYTNIFLRRIADG--FRDMTYSFVNQFNLASFDSIEVALLMILVVLNPDLEGLENRQAVLKLHKDYRTIFQKYCHETHGKLDNYLIMLQFVPYLAKIDLV
Hur2DBD-NR7B2 VKEELVFMSKVPGFKDLNPEDRNFVVQNMSFANILIIATKEWYNAKTKTFFQMFHLELP-----EWHPIYFYRKLAFQLAEEVHELKMDRTEAAMVSALNVIAGDCIGLKEPEKIEESRAFLISALRAHISYKGVDP--VSRMKEIFSLMPTFRLC
Mm2DBD-NRB1   TSAAVKFASQIPGFSNLHPEDKAFLCKSVTFMSTVLMAGQHLYSPSDKNFHDFWNWQIT-----PQNPFFPFKERLLEVGEKIHSANMDLYEVSVMAALIFLASDFLDLAYPEAIEEARAKVIGALKSYEMSKGVDV--HSRLTHLFSLIPEIRHV
Me2DBD-NRB1   NKAYVDFVMQIPGFMQLDHDDKQDLCQLASFQAALILCGTEWYNKQQKKFQNFWNFTVS-----PENPMYLFKLHLLQAGEVINKLELSPVELSLTLALSCFSADLMFLKDPESVESVRRHIIYLLQQHIAEQGINP--DERLAAIFNIMPMTRHI
Cg2DBD-NRB1   ITGFMNFVKTIPGFDSINSEDAAELCVMNSFHIAIILSANNYYDKEQKRFQYFWNWTLS-----PNNPMYFFKLRLLQCGEHLNRLNLTPMEETFLSVITFLSTDITNLRDPATLESLRQKMIHIFQAQLIAQGVDP--IQRIQELFRVMPDCRHC
Cv2DBD-NRB1   VSAFMNFVRTIPGFESINQEDAAELCIMNSFHIAIIVQANNYFDKEQKRYQYFWNWTLS-----PNNPLYFFKLRLLQCGEQLNQLNMTPLEETFLSIITFLSTDLLYLRDPATLESLRQKMIHILQAQLYAQGIDP--IKRMQELFRAMPDCRHC
Mc2DBD-NRB1   NKAYVDFCMQIPGFMQLDHEDKQDLCQLASFQAALILCSTEWYNKQQKKFQNFWNFTVS-----PENPMYLFKLHLLQAGEVINKLELSPVELSLTLALSCFSADLMFLKDPESIESVRRHLIYLLQQHIAEQGINP--DERLAAIFNIMPMTRHI
hVDR_cut      IQKVIGFAKMIPGFRDLTSEDQIVLLKSSAIEVIML----------------------------------------------------HEEEHVLLMAICIV--DRPGVQDAALIEAIQDRLSNTLQTYIRCRHP-----LLYAKMIQKLADLRSL
hVDR          IQKVIGFAKMIPGFRDLTSEDQIVLLKSSAIEVIMLRSNESFTMDDMSWTCGNQDYKYRVSDVTKAGHSLELIEPLIKFQVGLKKLNLHEEEHVLLMAICIVSPDRPGVQDAALIEAIQDRLSNTLQTYIRCRHPPPGSHLLYAKMIQKLADLRSL
hRXRa         LFTLVEWAKRIPHFSELPLDDQVILLRAGWNELLIASFSHR-SIAVKDGILLATGLHVHRNSAHSAGVGAIFDRVLTELVSKMRDMQMDKTELGCLRAIVLFNPDSKGLSNPAEVEALREKVYASLEAYCKHKYPEQ--PGRFAKLLLRLPALRSI
hRXRa1_Cut    LFTLVEWAKRIPHFSELPLDDQVILLRAGWNELLIA----------------------------------------------------DKTELGCLRAIVLF--DSKGLSNPAEVEALREKVYASLEAYCKHKYP-----GRFAKLLLRLPALRSI
```

**Echinodermata**

```
Ap2DBD-NRB   IIGSVTFAKNIPGFRHISVNDQMNLIKAGTFPCILCQLCWQGC-----MFSWFDDTFFTVPSLIKLVCG--MKTSCYSFREDFTKLTFDQEEIALFTALIFINPDNPGLDDVEFILGLHGKLRQVFKHYCLEKYGELKQYLKFISFLPRLYQIELL
Pmi2DBD-NRB  IIGSVNFAKNIPGFREIPVSDQMNLIKLGTFPSILCHLCWQGE-----EYSWLEHTFFTVPSLEALVCG--MKTSCYSFREDFSKVTFDEHEIALIMALIFINPDPSGLESVDFILNLHGKLRLIFKHYCLEKYGEMKQYLKFISFLPRLYKIELL
Aru2DBD-NRB  IISSVKFAKNIPGFCHISVSDQMSLIKMGTFPCILSHICWRAEL----EFSWFQDTFFTVPSLDALVFG--MKSKVYKFKEDFIQVKFDQHEIALFTTLIFINPDPPGLENSDYILKLHNKLRLILKHYCLEKYGELKQYYKYLSFLPRLYEIELV
Lv2DBD-NRB   IIASVQYIKKIPGFASIDMNDRINLIKHSVFPIILVHKAWKMGE----EWKWMEDTFFTNSSLRKMSDG--FKDMTYSFINQFNLGSFDSIEVALLMILVTLNPDLEGLENKEAVLKLHKDYRTIMQNYCLETHGKLDNYLIMLQFVPYLSKIDIV
Spur2DBD-NRB IIASVQYIKKIPGFPSIHMNDRLYLIKHSVFPIILVHKAWKMGQ----EWKWMEDTFYTNIFLRRIADG--FRDMTYSFVNQFNLASFDSIEVALLMILVVLNPDLEGLENRQAVLKLHKDYRTIFQKYCHETHGKLDNYLIMLQFVPYLAKIDLV
hRXRa        LFTLVEWAKRIPHFSELPLDDQVILLRAGWNELLIASFSHR-SIAVKDGILLATGLHVHRNSAHSAGVGAIFDRVLTELVSKMRDMQMDKTELGCLRAIVLFNPDSKGLSNPAEVEALREKVYASLEAYCKHKYPEQ--PGRFAKLLLRLPALRSI
hRXRa1_Cut   LFTLVEWAKRIPHFSELPLDDQVILLRAGWNELLIA----------------------------------------------------DKTELGCLRAIVLF--DSKGLSNPAEVEALREKVYASLEAYCKHKYP-----GRFAKLLLRLPALRSI
hVDR         IQKVIGFAKMIPGFRDLTSEDQIVLLKSSAIEVIMLRSNESFTMDDMSWTCGNQDYKYRVSDVTKAGHSLELIEPLIKFQVGLKKLNLHEEEHVLLMAICIVSPDRPGVQDAALIEAIQDRLSNTLQTYIRCRHPPPGSHLLYAKMIQKLADLRSL
hVDR_cut     IQKVIGFAKMIPGFRDLTSEDQIVLLKSSAIEVIML----------------------------------------------------HEEEHVLLMAICIV--DRPGVQDAALIEAIQDRLSNTLQTYIRCRHP-----LLYAKMIQKLADLRSL
```

**2DBD-NRC Nematode**

```
Cr2DBD-NRC3   NHQTGLFVNHLPFIKTFEINDKVILFKRSSFLMFVLRNITKFSSDGFMLPSKLNSKIKIPYETLKIVYGD-LLINEIISIA-SKLESMDLSHHELSLFTALVFFLPFTADAPERTRNKEKFKSHKTLELVYHCYKTFLNRMFSKYENGHDTISELDEMTFKLEDLNKL
Cr2DBD-NRC1   NHQTGLFVNRLPSIDSFEISDKAILFKRSSFLMFILRNITKFSSDGFMLPSKR-H---IPFRTMKTVYGD--LMNEIILVS-SDFKSMQLNHQELSLFTAFIFLRPITRGS----QDWQIFIGDSTLKGVRDYYRTLLYQLMAERENTQKIISDLATMTLKLYDLNKI
Cr2DBD-NRC4   AKQTLQFVRNLPHFNLFGMNDKIILFKKNCFLMFLLRHVLSFSKNGLQLPI-L-----IPYDTLAAVYGR--LITEIISLA-ENIKRMELKIQEIAVFTALVFFQPDGS---------TEIKDKERLEVVCEYYKLLLDQEMSERS--KK-LEKLVDLMPTLQKMNKD
CbNR7C        IRSLLPFIDELQ--DSFNSHDKAILLKRSAFQIYLLRRIRALNPYGLLLSDGR----VIKLISLQFLYGTP-LINEMLQVV--SRILQLGCTDEDIAMFITIVYYKPGRIKDLAS---LNLQNPENLSPAQQKFKNLFVKHAESRSVDQKVFQDLINLWPELERLSEF
hVDR          IQKVIGFAKMIPGFRDLTSEDQIVLLKSSAIEVIMLRSNESFTMDDMSWTCGN-QDYKYRVSDVTKAGHSLELIEPLIKFQ-VGLKKLNLHEEEHVLLMAICIVSPDRPG----------VQDAALIEAIQDRLSNTLQTYIRCRHPPPGSHLLYAKMIQKLADLRSL
hVDR_cut      IQKVIGFAKMIPGFRDLTSEDQIVLLKSSAIEVIML------------------------------------------------------HEEEHVLLMAICIV--DRPG----------VQDAALIEAIQDRLSNTLQTYIRCRHP-----LLYAKMIQKLADLRSL
hRXRa         LFTLVEWAKRIPHFSELPLDDQVILLRAGWNELLIASFSHR-SIAVKDGILLA-TGLHVHRNSAHSAGVGAIFDRVLTELV-SKMRDMQMDKTELGCLRAIVLFNPDSKG----------LSNPAEVEALREKVYASLEAYCKHKYPEQ--PGRFAKLLLRLPALRSI
hRXRa1_Cut    LFTLVEWAKRIPHFSELPLDDQVILLRAGWNELLIA------------------------------------------------------DKTELGCLRAIVLF--DSKG----------LSNPAEVEALREKVYASLEAYCKHKYP-----GRFAKLLLRLPALRSI
```
